# Eplerenone, a mineralocorticoid receptor inhibitor, reduces cirrhosis associated changes of hepatocyte glucose and lipid metabolism

**DOI:** 10.1186/s12964-024-01991-2

**Published:** 2024-12-20

**Authors:** Mohammad Mohabbulla Mohib, Sindy Rabe, Alexander Nolze, Michael Rooney, Quratul Ain, Alexander Zipprich, Michael Gekle, Barbara Schreier

**Affiliations:** 1 Julius-Bernstein-Institute of Physiology, Martin Luther University Halle-Wittenberg, Magdeburger Strasse 6, 06112 Halle (Saale), Germany; 2 Department of Internal Medicine IV, Jena University Hospital, Friedrich-Schiller-University Jena, Am Klinikum 1, 07747 Jena, Germany; 3 Julius-Bernstein-Institut für Physiologie, Universität Halle-Wittenberg, Magdeburger Strasse 6, 06112 Halle (Saale), Germany

**Keywords:** Cirrhosis, Mineralocorticoid receptor, Hypoxia, Metabolism

## Abstract

**Background:**

Recent studies suggest a contribution of intrahepatic mineralocorticoid receptor (MR) activation to the development of cirrhosis. As MR blockade abrogates the development of cirrhosis and hypoxia, common during the development of cirrhosis, can activate MR in hepatocytes. But, the impact of non-physiological hepatic MR activation is unknown. In this study, we investigate the impact of hypoxia-induced hepatocyte MR activation as a relevant factor in cirrhosis.

**Methods:**

RNA sequencing followed by gene ontology term enrichment analysis was performed on liver samples from rats treated for 12 weeks with or without CCl_4_ and for the last four weeks with or without eplerenone (MR antagonist). We investigated if these changes can be mimicked by hypoxia in a human hepatocyte cell line (HepG2 cells) and in primary rat hepatocytes (pRH). In order to evaluate the functional cellular importance, hepatocyte lipid accumulation, glucose consumption, lactate production and mitochondrial function were analyzed.

**Results:**

In cirrhotic liver tissue genes annotated to the GOterm “Monocarboxylic acid metabolic process” (PPARα, PDK4, AMACR, ABCC2, Lipin1) are downregulated. This effect is reversed by the MR antagonist eplerenone in vivo. The alterations are partially mimicked by hypoxia in rat and human hepatocytes in tissue culture. Furthermore, the reduction of mRNA and protein expression of PPARα, PDK4, AMACR, ABCC2 and Lipin1 during hypoxia is prevented by eplerenone in rat and human hepatocytes. Aldosterone, the endogenous MR agonist, did not affect the expression of those proteins in hepatocytes. As those proteins are key regulators of hepatocyte energy homeostasis, we analyzed if hypoxia affected glucose consumption, lactate production and lipid accumulation in HepG2 cells in a MR-mediated manner. All three parameters were affected by hypoxia and were partially normalized by eplerenone.

**Conclusion:**

Our findings suggest that non-physiological MR activation plays a role in the dysregulation of glucose and lipid metabolism in hepatocytes. This leads to an increase in apoptosis, probably resulting in a proinflammatory micromilieu of the hepatic tissue. The enhanced deposition of extracellular matrix contributes to the development of cirrhosis. Therefore, MR antagonists may have therapeutic potential in the treatment of early stages of liver disease due to their direct action in the liver.

**Supplementary Information:**

The online version contains supplementary material available at 10.1186/s12964-024-01991-2.

## Background

About 2 million people per year die worldwide due to liver diseases [1, 2]. Cirrhosis is the end-stage of chronic liver diseases and the ninth most common cause of death with rising numbers in Europe [2]. Cirrhosis leads to disturbed liver architecture due to fibrosis [3] and to the development of portal hypertension as the main driver for other complications like variceal bleeding and ascites [4]. The reversibility of fibrosis has been demonstrated in nearly all chronic liver diseases after cessation of the causative agent [5], leading to a significant increase in 10-year survival. Therefore, termination of progression of fibrosis most likely has important implications for the patient outcome [6]. This rational lead to the recommendation of the Baveno conferences to explore antifibrotic strategies to prevent decompensation in cirrhosis [7, 8]. Amongst others, the renin–angiotensin–aldosterone system (RAAS) becomes dysregulated during cirrhosis [9] and is considered as an area for further investigations [7, 8].

One of the effectors of the RAAS is aldosterone [10]. Under physiological conditions this steroid hormone binds to its canonical receptor - the mineralocorticoid receptor (MR) - thereby increasing renal and enteric sodium as well as water reabsorption. Thus, aldosterone contributes to extracellular volume and blood pressure balance [10–12]. In non-epithelial cells MR mediates fibrosis, hypertrophy and remodelling initiated by inflammation [13]. MR increases reactive oxygen species formation itself but is also activated by oxidative stress [10]. These pathological effects of MR are at least partially independent of its classical ligand aldosterone [14]. In the healthy liver the MR is mainly expressed in hepatocytes [15, 16].

At present, RAAS inhibitors are widely used in patients with predisposing conditions to prevent the development of cardiac and renal fibrosis, but no antifibrotic treatment in liver fibrosis is established [17]. Animal studies showed, that administration of MR blockers prevent the progression of fibrosis while administration of aldosterone supported the development of liver fibrosis [18, 19]. One antagonist of the MR is eplerenone [20]. In CCl_4_ treated rats with liver fibrosis we were able to demonstrate that treatment with eplerenone, although continuing the CCl_4_ application, was able to reduce fibrosis development, spleen weight and increase liver weight [16]. We identified the hepatocytes as the main cell type expressing the MR under physiological conditions. This is of interest as hepatocytes are at the center of initial damage and vice versa liver regeneration [21]. Analyzing the MR expression in rats with or without liver cirrhosis revealed that MR mRNA as well as MR protein are reduced. Further analysis disclosed that MR protein reduction in hepatocytes was more pronounced than mRNA reduction, indicating a transcriptional effect on MR expression as well as effects affecting protein abundance [16]. As hypoxia induces a translocation of the MR from the cytosol to the nucleus, we concluded that the reduction in MR protein amount is at least partially a consequence of the previous activation [16]. We also demonstrated that hypoxia leads to an increase in NF-κB-response element activity in the presence of aldosterone and reduces GRE-response element activation in the absence of aldosterone by the MR [16]. For humans two different promoters of the MR [22] have been described and different promoters are at least assumed in the rat [23]. Therefore, we focused in this study on the effects of the MR protein in hepatocytes. Identifying factors altering the transcription of the MR is beyond the scope of this study.

Several micromileu factors change during the development of liver fibrosis including the oxygen content of the tissue due to structural remodeling. The aim of this study was to exclude tissue acidosis or inflammation [3] as MR activators which coincide with hypoxia during cirrhosis [3, 24, 25]. Additionally, we aimed to characterize the impact of MR activation in hepatocytes under hypoxic conditions as they occur in liver cirrhosis. Herein we demonstrate that hepatocyte MR is activavted by hypoxia but not by lipopolysaccharide or acidosis. Using a CCl_4_ model of cirrhosis development, we performed RNAseq on rat livers from control, cirrhosis and cirrhosis & eplerenone treated groups. The results indicate that MR activity influences carbohydrate metabolism and lipid accumulation. We confirmed these findings in a human hepatocyte cell line (HepG2) on protein level and demonstrate that under hypoxic conditions eplerenone can partially normalize glucose metabolism as well as lipid accumulation in these cells, which can not be explained by a change in mitochondrial function. Trying to mimick the in vivo situation, we found that in the presence of free fatty acids the inhibition of the MR in reoxygenated hepatocytes leads to a reduction in apoptosis. In conclusion, the MR in hepatocytes aggravates metabolic imbalance in liver cirrhosis and promotes thereby the progression of cirrhosis.

## Methods

If not stated otherwise all chemicals were purchased by Merck, Darmstadt, Germany.

### Induction of cirrhosis

All animal experiments were conducted in accordance with the german law (Tierschutzgesetz) and the directive 2010/63/EU. They were ethically and legally approved by the governmental, local animal committees (42502-2-1123 MLU, Landesverwaltungsamt Sachsen-Anhalt or TWZ 16-2021, Jena University hospital). All animal studies were conducted in compliance with the ARRIVE guidelines [26] and the National Institutes of Health (NIH) Guide for the Care and Use of Laboratory Animals. The rats were kept in a specific pathogen-free environment, with *ad libitum* access to water and standard rodent diet at constant temperature of 22 ± 2 °C with a 12/12 h light-dark cycle. Animals were housed in groups of three if applicable and randomly assigned to the experimental groups. Rats were exposed to carbon tetrachloride (CCl_4_) via inhalation for 3 to 4 min, three times a week. According to Sakata et al. [27] this will result in a concentration of about 180 ppm CCl_4_. Phenobarbital (0.35 g/L) was added to their drinking water (*ad libitum*) to induce cirrhosis. The treatment was administered for 12 weeks in age-matched, male Wistar rats (purchased from Charles River) with an initial weight of 75–100 g as described before [16, 28–31]. The treatment combination of CCl_4_ and phenobarbital will be abbreviated as CCl_4_. Eplerenone treatment (Inspra, Pfizer Pharma PFE GmbH, Berlin, Germany, 100 mg kg^-1^ BW) per os (hazelnut spread, 5 g/day) was initiated after eight weeks of CCl_4_ inhalation, which marks a time point of established cirrhosis without ascites, and continued until 12 weeks of CCl4 inhalation, at which point the rats not receiving eplerenone had developed cirrhosis with ascites. Livers were isolated 7–10 days after the last dose of CCl_4_.

At the end of the experiment, the animals were anesthetized with ketamine/xylazine (100/5 mg/kg i.p.; Ketaset 100 mg/ml, Zoetis, 2% Rompun, Bayer Healthcare) and euthanized by exsanguination while in deep narcosis. Cells or organs were harvested as described previously [16, 32, 33].

### RNA sequencing

RNA sequencing was performed as described before [34]. The quality of the RNA samples was assessed using a 2100 Bioanalyzer (Agilent Technologies). All samples had an RNA Integrity Number (RIN) above 7 (with 10 as the maximal possible value). Novogene Co., Ltd. (Cambridge, UK) carried out the sequencing, libraries preparation (poly(A) enrichment), and the paired-end sequencing (2 × 150 bp) runs on a NovaSeq6000 Illumina system. Adaptor clipping and data quality control were provided by the service company as well.

Read mapping to the rat genome rn6 was performed with HISAT2 (v. 2.1.0) [35], and featureCounts (2.0.0, –M –t exon) [36] was used to count the mapped reads. Gene annotation was performed using BiomaRt (v.2.44.4) [37] to access the Ensembl archive v101.

### Differential expression analysis and functional analysis

Differential expression analysis was performed as described before [34] using edgeR (3.30.3) [38] and DESeq2 (1.28.1) [39]. Significantly “differentially expressed genes” (DEG) were defined as genes with a false discovery rate (FDR) below 0.05 in both DESeq2 and edgeR outputs (overlap of the respective results). Only protein coding genes were considered for further analysis.

Gene ontology (GO) term enrichment analysis was performed with g:Profiler [40] using either all or only down or up-regulated eplerenone sensitive genes against the whole genome first. GO terms were defined as significantly enriched if the adjusted p-value ≤ 10^− 6^. AmiGO (2.5) [41] was used to visualize the gene ontology trees for up- or downregulated genes. Further analysis are based on the datasets available on the 25th of May, 2022. Additionally, we performed GO term enrichment analysis with g:Profiler for the all eplerenone sensitive, protein coding genes as well as separately for up- and downregulated genes. The results are given in supplementary excel file 1.

In the results section the detailed strategy to identify eplerenone sensitive genes in cirrhosis is described. Additionally, we employed the following strategy, to identify eplerenone-sensitive genes in cirrhosis: (1) At least in one group the mean abundance has to be > 3 FPM. (2) |log2FC| > 1 for the comparison control vs. cirrhosis. (3) 95% confidence intervals of the control samples and the cirrhosis samples do not overlap. (4) The confidence intervals for cirrhosis and cirrhosis & eplerenone do not overlap. (5) The confidence intervals for control and cirrhosis & eplerenone do overlap. The strategy is depicted in the flow chart in supplementary figure S1. By this strategy we identified 631 genes to be eplerenone-sensitve. Of those genes 337 were downregulated and 294 were upregulated. About 80% of those genes have been identfied before (Supplementary figure S2). We included the respective results in the supplementary ecxel file 2 and added a supplementary figure S3 showing the confidence intervalls for investigated genes.

### Cell culture

Cells were maintained in a standard cell incubator at 37 °C, 5% CO_2_, 70% humidity. The isolation of rat primary hepatocytes (pRH) was performed as described by Graupera et al. (2005) [33]. Cells were kept in hepatocyte growth medium (HGM), supplemented with fetal calf serum (FCS, PAN-Biotech and 1% P/S [42]. HepG2 cells (ATCC, Manassas, Virginia, USA) were grown in RPMI 1640 (PAN-Biotech) medium supplemented with 10% FCS.

Prior to experiments, cells were incubated for 24 h in serum free conditions buffered with NaHCO_3_, 10mM morpholinoethanesulfonic acid and 10mM HEPES with a pH adjusted to 7.4. For acidosis the pH of serum free medium was adjusted to 6.6 with 1 N HCl. Hypoxia was induced in a hypoxia chamber (HypoxyLab) at 0.2% O_2_ (pO_2_ = 1.5 mmHg) for HepG2 and 1% O_2_ (pO_2_ = 7.4 mmHg) for pRH as reported before [16]. To mimick bacterial translocation hepatocytes were incubated with 1 µg/mL or 10 µg/mL lipopolysaccharides from *Escherichia coli* (Sigma, O111:B4) dissolved in water. Experimental incubation was carried out for 24 h if not stated otherwise.

HepG2 cells were treated with free fatty acids, either under normoxic or hypoxic conditions with or without eplerenone. Sodium oleate and sodium palmitate (each 100 µM) were added to fatty acid free bovine serum albumin (Capricorn Scientific GmbH) before introducing the solution into media. Glucose consumption, lactate production, oxygen consumption rate (OCR) and lipid accumulation were measured after 4-hour. Lipid accumulation, lactate dehydrogenase activity and caspase activity were measured after 24-hours.

### Quantitative PCR

Total RNA was isolated as described before [16] using Trizol (Invitrogen) or BlueZol (SERVA Electrophoresis GmbH) according to the manufacturer’s instructions. Genomic DNA was eliminated by digestion of one µg RNA with 2 units of DNase I (RNase-free; New England Biolabs, Frankfurt, Germany) at 37 °C for 10 min, followed by enzyme inactivation at 75 °C for 10 min. The DNase I-treated total RNA was subjected to reverse transcription (RT) reaction with random primers, using SuperScript II reverse transcriptase (Invitrogen, Massachusetts, USA). The RT reaction was carried out with the following program: 5 min at 25 °C, 30 min at 42 °C, and 5 min at 95 °C.

qRT-PCR was performed using the AriaMx real-time PCR system (Agilent Technologies). To determine the differential expression of genes, the expression levels were normalized to the levels of eukaryotic 18 S rRNA and HPRT and to the control group. Data is given as percent of control. The primer sequence, annealing temperature and RefSeq accession number/id are given in supplementary table S1 (rats) or in supplementary table S2 (human).

### Western blot analysis

Western blot analysis was performed as described before [16]. The following antibodies were utilized in this study: anti-MR (1:500; rMR 1–18 6G1) [43]; anti-PPAR alpha (1:2000, ab126285; Abcam); anti-PDK4 picoband (1:2000, PB9773, Boster Bio); anti-MRP2/ABCC2 (1:1000; #4446; Cell Signaling Technology); anti-Lipin 1 (1:1000; #5195; Cell Signaling Technology); anti-AMACR (1:2000; #3207, Cell Signaling Technology) ); anti-HSP90 (1:2000; #4874; Cell Signalling Technology). Horseradish peroxidase conjugated secondary IgG (anti-mouse, 1:1000, 7076P2, horse origin; Cell Signaling Technology; anti-rabbit, 1:2000, 7074P2, goat origin; Cell Signaling Technology) and the ECL system (Biorad) were used for visualisation. Density of protein bands was quantified using ImageLab 6.1 (Biorad) and normalized to HSP 90 expression or whole protein stained by Ponceau staining.

### Determination of glucose consumption, lactate production and lipid accumulation and oil red O staining

Analysis of glucose consumption and lactate production were performed as described before [44].

After 24 h of pretreatment with hypoxia or normoxia treatment with free fatty acid containing media (100µM sodium oleate & sodium palmitate each and 16.5 µM fatty acid free bovine serum albumin; Capricorn Scientific GmbH, Ebsdorfergrund, Hessen, Germany) was performed. The supernatant from HepG2 cells was removed and Oil red O staining was performed as described by Kraus et al. (2016) [45]. Briefly, 0.5% w/v Oil red O stock solution in 2-propanol was prepared. For use stock solution was diluted 2:3 with distilled water, resulting in a concentration of 0.2% oil red O in 40% 2-propanol. For each replicate 2 × 200 µl were transferred to a 96-well plate. Absorption was measured at 510 nm in an Infinite M200 (Tecan). The values were normalized to the total protein amount in the sample. Protein content was determined described by Sadick et al. [46] using the BCA assay (Thermo Fisher Scientific) according to the manufacturer’s instructions.

### Caspase activity

For the caspase-3 activity assay, HepG2 cells were lysed with MOPS-Triton lysis buffer (30 µl/cm2, 30 min, 4 °C) followed by centrifugation at 13,000×g for 5 min at 4 °C. Cleared lysates (40 µl) were then incubated with DEVD-AFC (40 µM) in caspase reaction buffer (50 mM PIPES, 10 mM EDTA, 0.5% CHAPS, pH 7.4; 120 min, 37 °C). AFC fluorescence (excitation: 400 nm; emission: 505 nm) was measured using a plate reader (Infinite M200, Tecan, Crailsheim, Germany). Cleaved AFC levels were quantified via a calibration curve and normalized to the protein content of each sample.

### Lactate dehydrogenase (LDH) activity

The LDH assay was conducted to measure the enzyme activity in both media and cell lysates using established procedures [47]. Supernatants and lysates from HepG2 cells were treated with LDH substrate buffer (Hepes-Ringer buffer + NADH + Pyruvate), and the turnover of LDH substrate was monitored at 334 nm (NADH) for 30 min at 37 °C. Relative LDH release was determined by correlating the measured activity with the total cellular LDH content.

### Oxygen consumption rate (OCR)

The Oxygen Consumption Rate (OCR) was determined in HepG2 cells cultured in 96-well Seahorse XF96 V3 PS Cell Culture Microplates (Agilent Technologies, Waldbronn, Germany). Real-time OCR was assessed using the Seahorse XF Cell Mito Stress Test Kit and Seahorse XFe96 Analyzer (Agilent Technologies, Waldbronn, Germany), following the manufacturer’s instructions.

The mitochondrial stress test involved measuring OCR under basal conditions, followed by the sequential addition of inhibitors. Oligomycin (2 µM) was first added to inhibit complex V (ATP Synthase) of the electron transport chain (ETC). Subsequently, FCCP (4 µM) was introduced to collapse the proton gradient and disrupt the mitochondrial membrane potential. Finally, rotenone and antimycin A (0.5 µM each) were injected to inhibit complexes I and III, respectively. Hoechst 33,342 (6 µM) was added during the final injection to normalize OCR to the cell number, and its induced nuclear staining was detected using digital fluorescence microscopy (Cytation3, BioTek, Bad Friedrichshall, Germany).

From these measurements, the following parameters were calculated: non-mitochondrial respiration (NMOC, OCRmin_Rotenone/AntimycinA), basal respiration rate (OCRinitial value - OCRmin_Rotenone/AntimycinA), mitochondrial ATP production-linked oxygen consumption rate (OCRinitial value - OCRmin_Rotenone/AntimycinA), proton leak (OCRmin Oligomycin- OCRmin_Rotenone/AntimycinA) and coupling efficiency (((basal respiration + NMOC)-(proton leak + NMOC))/(basal respiration + NMOC)).

### Statistical analysis

Data are given in boxplots depicting median and standard deviation. Either Grubbs outlier tests (cut off criterion *P* < 0.05) or Iglewicz and Hoaglin’s robust test for multiple outliers with a modified Z-score ≥ 3.5 were performed. Data analysis was conducted by SigmaPlot 12.5. Shapiro-Wilks test was performed for testing of normal distribution of the data. According to those results differences between groups were assessed by Two-way ANOVA, two-tailed Students T-test or Mann-Whitney Rank Sum Test as applicable. A p-value < 0.05 was considered significant. N = number of animals or independent experiments, n = number of biological replicates.

## Results

### Hypoxia but not LPS or acidosis lead to enhanced MR activation in hepatocytes

**Fig. 1:**
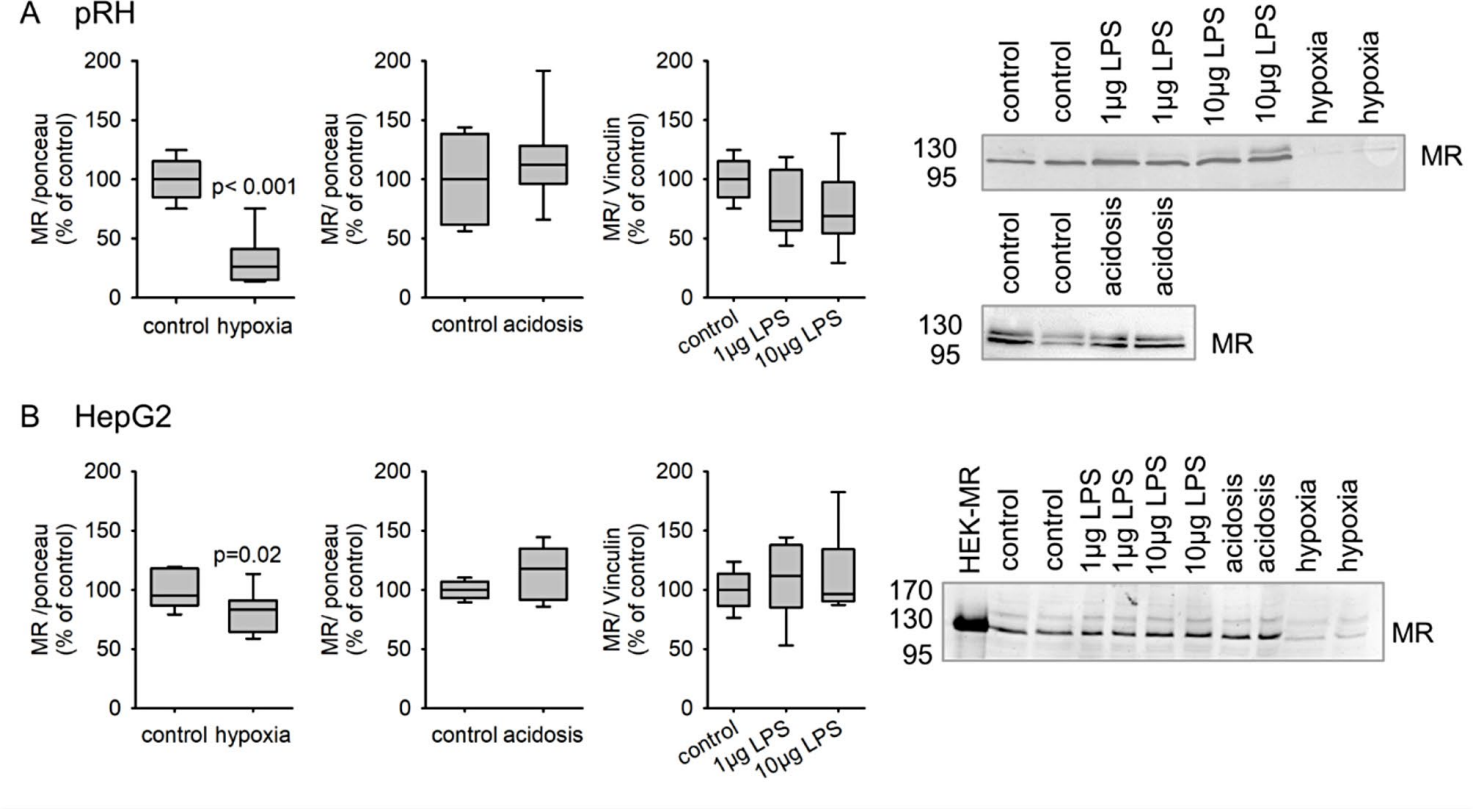
Protein expression of the mineralocorticoid receptor in primary rat hepatocytes (**A**; *N* = 3–5 experiments, *n* = 8–10 petri dishes) and a human hepatocyte cell line (HepG2, **B**; *N* = 4 experiments, *n* = 8–10 petri dishes) 24 h after incubation either with hypoxia (0.2% oxygen), acidosis (pH 6.4) or lipopolysaccharides (LPS, 1–10 µg/L). p-values are given for the comparison vs. control if the p-value was below 0.05.On the right side representative Western blots for the MR from primary rat hepatocytes or HepG2 cells are presented

Among the micromilieu factors changing during the development of liver fibrosis are the oxygen content of the tissue due to structural remodeling, the translocation of bacteria and with this inflammation [3], resulting in tissue acidosis. We already demonstrated that lack of oxygen induces MR activation without further addition of its ligand aldosterone [16]. To exclude bacterial proinflammatory substances (LPS) or tissue acidosis as factors increasing MR activation we incubated pRH as well as HepG2 cells with either LPS (1–10 µg), acidosis (pH 6.6) or hypoxia (0.2% or 1% oxygen) for 24 h. We demonstrated that MR activation leads to nuclear shuttling of the MR and transcriptional changes followed by degradation of the MR, as demonstrated before [16]. Therefore, in a steady state activation of the MR will lead to a reduced protein content, which would be pronounced by a reduced transcription. Under these disease associated conditions we again found that hypoxia induces a down regulation in MR protein, whereas LPS treatment or acidosis showed no effect on MR expression in HepG2 cells or pRH (Fig. 1). This would indicate a steady state condition of MR protein. Under normoxic conditions activation of the MR by aldosterone enhanced the expression of SGK-1, PAI-1, MCP-1, Fn-1 and α-SMA in pRH (Supplementary table S3) as well as SGK-1, PAI-1 and TGF-β in human HepG2 cells (Supplementary table S4). Those effects could be inhibited by simultaneous incubation with aldosterone and eplerenone under normoxic conditions. Hypoxia alone altered the expression of SGK-1, Fn-1, TGF-β and α-SMA in pRH as well as SGK-1, PAI-1 and TGF-β in HepG2 cells in comparison to normoxia. Activation of MR by aldosterone under hypoxic conditions had no major effect on gene expression compared to hypoxia alone (pRH: Supplementary table S3, HepG2: Supplementary table S4). Under hypoxic conditions eplerenone had only an inhibitory effect on the expression of α-SMA in pRH, while it had no effect on the other aforementioned genes. In HepG2 cells, eplerenone reduced the expression of SGK-1, PAI-1 and TGF-β under hypoxic conditions. In summary, these findings indicate, that the genotropic effect of the mineralocorticoid receptor is altered under hypoxic conditions.

### RNAseq reveals that under cirrhotic conditions eplerenone mainly impacts downregulated genes

**Fig. 2:**
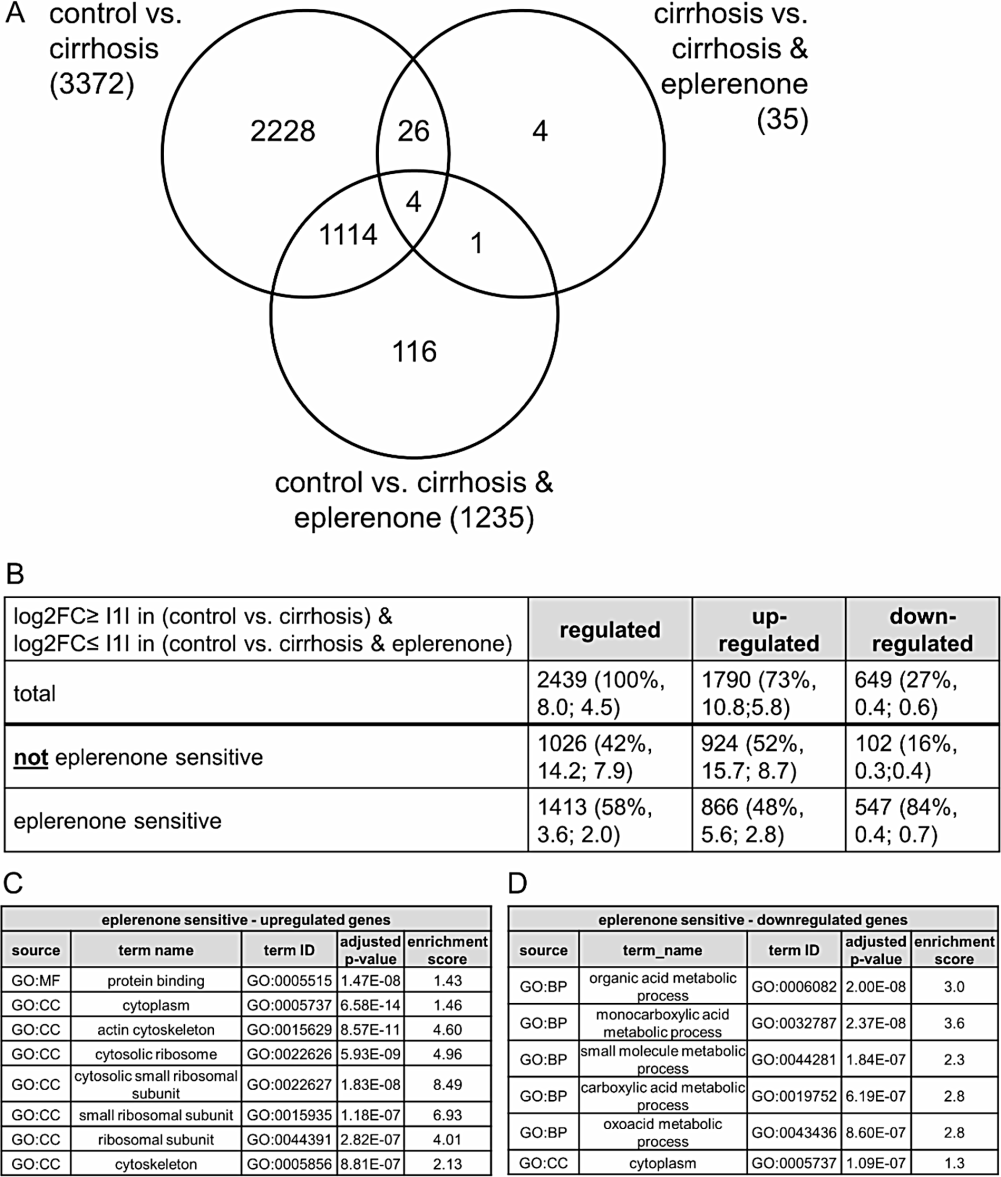
Bioinformatic analysis of RNA-seq data. (**A**) Venn diagram showing the distribution of the differentially expressed genes detected. Table (**B**) showing the distribution of eplerenone sensitve and not eplerenone sensitve genes in cirrhosis. In brackets the percentage of regulated genes is given as well as the mean fold-change of the respective genes for the comparison control vs. cirrhosis and control vs. cirrhosis & eplerenone. Differentially expressed genes were used for GO term enrichment analysis. The results are depicted in the lower tables separately for up- (**C**) and downregulated (**D**), eplerenone sensitive genes in liver cirrhosis. Only GO: terms with an adjusted p-value ≤ E-07 were taken into account. Enrichment scores are given

To identify MR responsive genes in the cirrhotic liver, we performed RNA sequencing on whole RNA from livers of either control treated or CCl_4_ treated rats for 12 weeks to induce cirrhosis. The CCl_4_ treated rats were divided in two subgroups with or without oral eplerenone supplementation starting after 8 weeks of CCl_4_ treatment (schematic treatment plan see supplementary figure S4) as described before [16]. Five samples of each group were sequenced. Principal component analysis indicated that one sample of the control group was different from the four others and was excluded from further analysis. Of 14,417 genes detected in total, 13,628 were protein coding genes. In the following we performed DESeq2 and edgeR analysis. The results for the differential expression analysis are given in supplementary figure S5. Only genes differentially expressed in both DESeq2 and edgeR analysis were further considered and the distribution is depicted in the Venn diagram (Fig. 2A). In summary, 3493 genes were differentially expressed in all three comparisons (Fig. 2A). For further analysis we considered only genes with an absolute Ilog2FCI ≥ 1 in the comparison between control and CCl_4_-treated animals (*cirrhosis sensitive genes*) as relevant. Cirrhosis induced the regulation of 2439 genes (Fig. 2B), the majority being upregulated (1790 genes, 73%). As reported before, eplerenone reduces the clinical signs of cirrhosis in this model [16]. Therefore, we aimed to identify eplerenone sensitive genes in cirrhosis. Accordingly, we identified genes that were differentially expressed when comparing the control and cirrhosis group (Ilog2FCI ≥ 1 in control vs. cirrhosis, prerequisite 1), but that were no longer differentially expressed (i.e. “normalized”) comparing control and cirrhosis & eplerenone animals (not identified as differential expressed by DESeq2 & edgeR & Ilog2FCI ≤ 1 in the comparison control vs. cirrhosis & eplerenone, prerequisite 2). Genes that met both criteria were termed “*eplerenone sensitive genes”*. Of the cirrhosis sensitive genes, a slightly bigger fraction were eplerenone sensitive (58%). Interestingly, from the genes that were downregulated by cirrhosis, 84% (547 genes) were eplerenone sensitive and only 16% were not eplerenone sensitive. Indicating a higher impact of eplerenone under cirrhotic conditions on downregulated genes.

We performed gene ontology enrichment analysis separately for up- and downregulated eplerenone sensitive genes. GO terms were filtered for the adjusted p-value and only GO terms with an adjusted p-value ≤ 1E^− 7^ were considered. The list of the respective GO terms is given in Fig. 2C for the genes upregulated and in Fig. 2D for the genes downregulated by cirrhosis. Based on the tree-like organization of gene ontology, the common child term significantly enriched was identified for up- and downregulated genes. For the upregulated genes this was cytosolic small ribosomal subunit (GO:0022627) and actin cytoskeleton (GO:0015629, the respective tree is given in supplementary figure S6). For the downregulated genes this was monocarboxylic acid metabolic process (GO:0032787, tree is given in supplementary figure S7). We repeated the analysis with the expressed genes as background (Supplementary excel file 1). Although the adjusted p-values were reasonably higher still virtually the same Go terms were significantly enriched. For further analysis we concentrated on genes annotated in the GO terms cytosolic small ribosomal subunit and monocarboxylic acid metabolic process. The RNA sequencing results for a selection of genes included in the respective GO terms are given in supplementary figure S8A for upregulated genes and in supplementary figure S8B for downregulated genes.

### Eplerenone treatment inhibited downregulation of monocarboxylic acid metabolic process related genes in cirrhotic rat livers

As a subgroup of the animal samples were selected for RNA sequencing by phenotypical success of cirrhosis induction and/or eplerenone treatment, we analyzed in a next step the expression of selected genes in livers from all experimental rats.

**Fig. 3:**
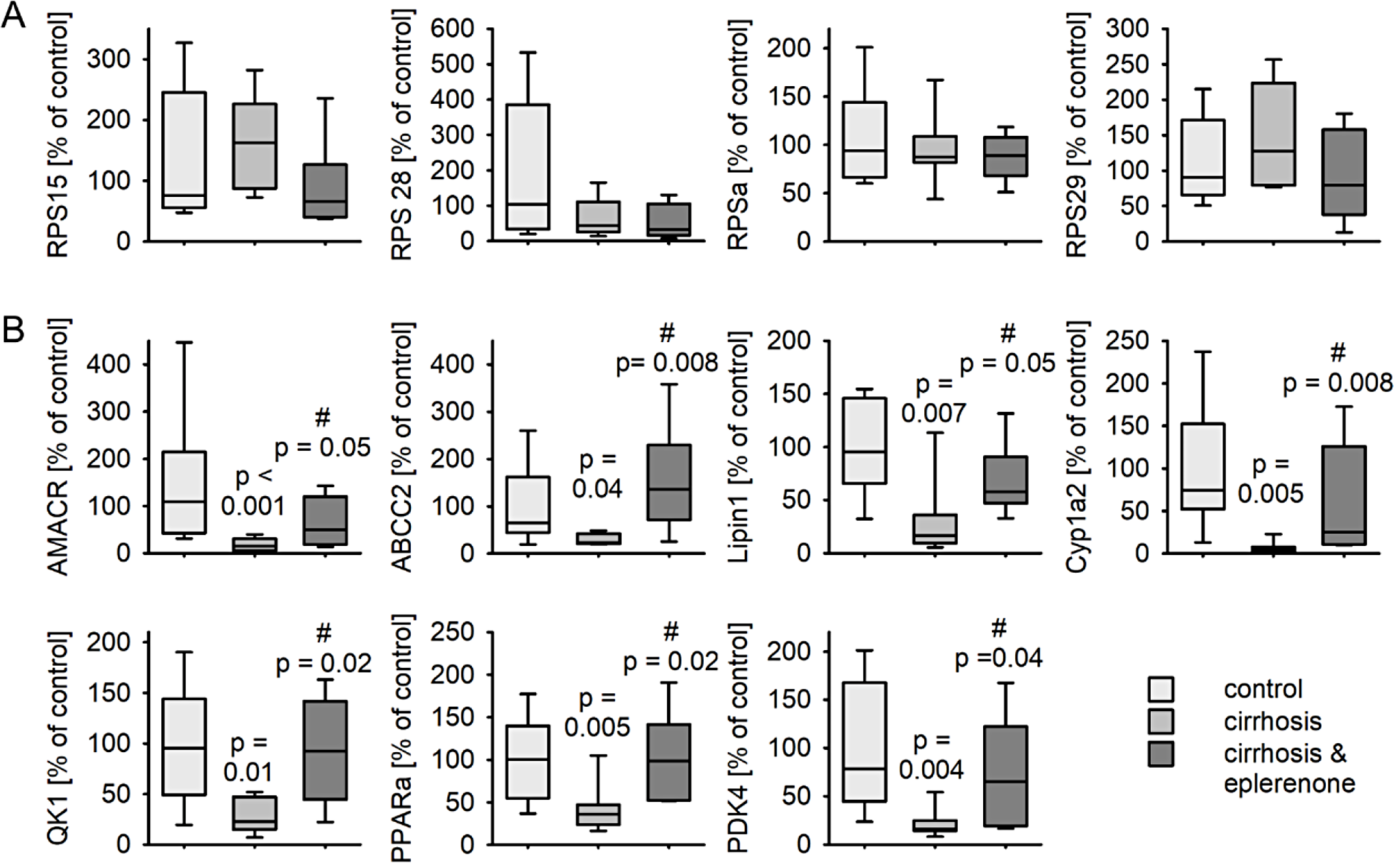
Expression of representative, differentially expressed genes identified by RNA sequencing in an extended cohort of liver samples. qRT-PCR of RNA from rat livers either from not treated (control) rats or from animals after 12 weeks of CCl_4_ & phenobarbital treatment (cirrhosis) or after 12 weeks of CCl_4_ & phenobarbital treatment accompanied by eplerenone treatment for the last four weeks (cirrhosis & eplerenone). *N* = 6–8 animals/ group, when below 0.05 p-values are given for the comparison vs. control. # indicates the p-value for the comparison vs. cirrhosis

For validation, we chose four eplerenone sensitive upregulated genes from the GO term cytosolic small ribosomal subunit (GO:0022627) and seven eplerenone sensitive downregulated genes from the GO term monocarboxylic acid metabolic process (GO:0032787). In the extended cohort we could not confirm that any of the selected genes annotated in the GO term cytosolic small ribosomal subunit were upregulated in livers from animals with cirrhosis compared to control (Fig. 3A). In contrast to the RNA sequencing results, eplerenone was not able to reduce the expression of the four genes compared to cirrhosis. Regarding the expression of the genes annotated in the term monocarboxylic acid metabolic process a downregulation for AMACR, ABCC2, Lipin1, Cyp1a2, QK1 (LOC108348175), PPARα and PDK4 was confirmed in the extended cohort. Eplerenone treatment increased the expression of those genes compared to animals of the cirrhosis group, thereby achieving similar levels to that of the control animals (Fig. 3B).

### Hypoxia induces MR-dependent downregulation of genes involved in lipid and glucose metabolism in hepatocytes

**Fig. 4:**
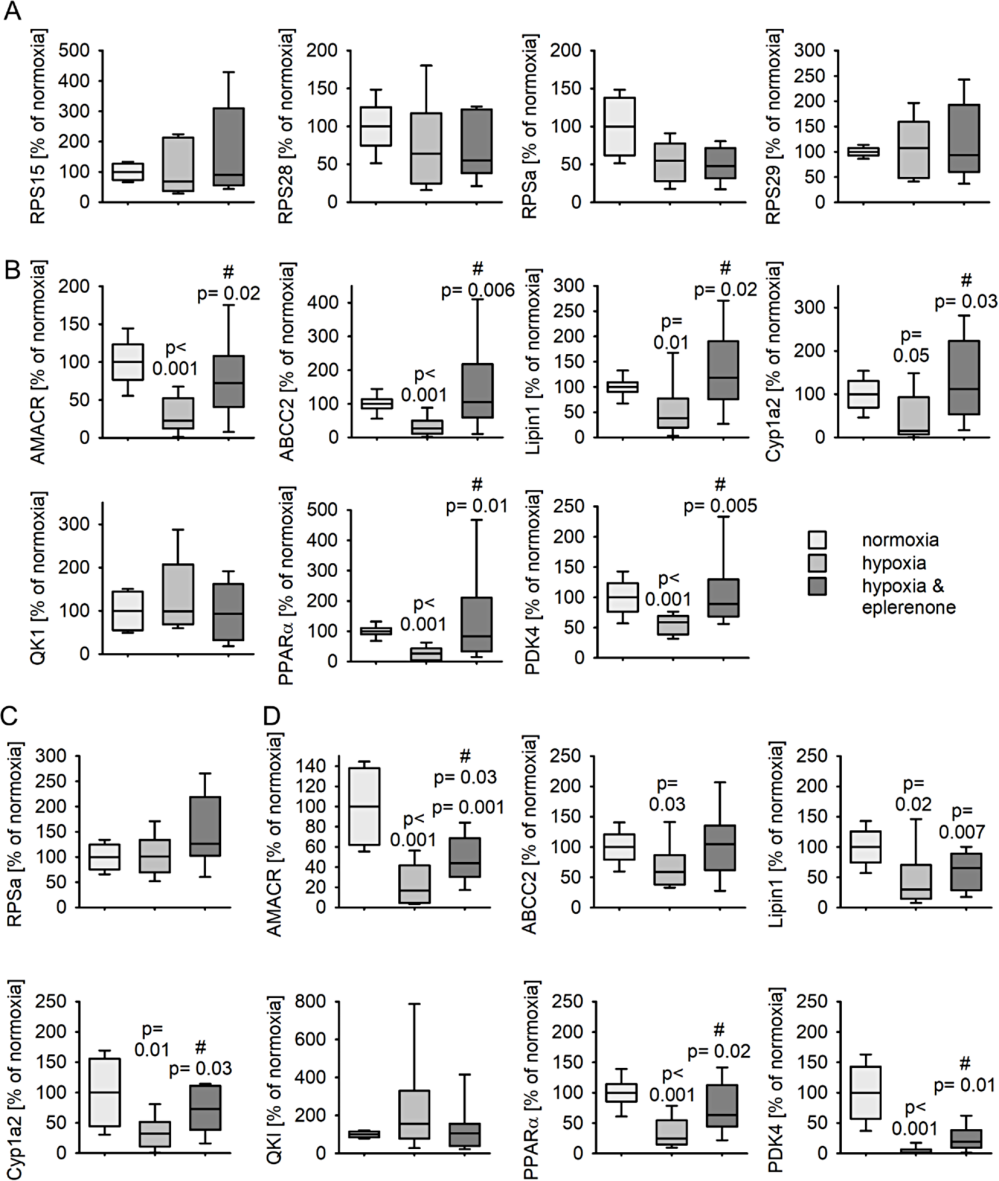
Change of expression of representative, differentially expressed genes identified by RNA sequencing from cirrhotic rat livers in primary rat hepatocytes or HepG2 cells upon treatment with hypoxia. **A & B**) Primary rat hepatocytes were treated for 24 h either under normoxic (16% oxygen) or hypoxic (1% oxygen) conditions with or without eplerenone (5µM). *N* = 4 experiments, *n* = 9–10 petri dishes/group. **C & D**) HepG2 cells were treated for 24 h either under normoxic (16% oxygen) or hypoxic (0.2% oxygen) conditions with or without eplerenone (5µM). *N* = 5 experiments, *n* = 9–10 petri dishes/group, when below 0.05 p-values are given for the comparison vs. normoxia, control. # indcates the p-value for the comparison vs. hypoxia, control

In our previous publication we demonstrated that the main cell type expressing the MR under control conditions are the hepatocytes and that hypoxia induced the activation and translocation of the MR into the nucleus [16]. Consequently, we incubated freshly isolated hepatocytes from healthy rats for 24 h with 1% O_2_, 5% CO_2’_ at 37 °C and 100% humidity. We analyzed if hypoxia reduces the expression of the genes annotated in the GO term “cytosolic small ribosomal subunit”. The expression did not change with hypoxia or with hypoxia & eplerenone treatment (Fig. 4A). In contrast, hypoxia reduced the expression of the eplerenone-sensitive genes ABCC2, PPARα, PDK4, Lipin1, Cyp1a2 and AMACR but not of QKI (Fig. 4B). Eplerenone inhibited the reduction of expression at least partially in primary rat hepatocytes. To ensure that this effect is reproducible in cells of human origin, we treated HepG2 cells for 24 h with 0.2% of oxygen with or without 5 µM eplerenone. In HepG2 cells, hypoxia did not increase the expression of RPSa (Fig. 4C) but reduced the expression of PPARα, PDK4, Lipin1, Cyp1a2 and AMACR but not of QKI (Fig. 4D). This effect was at least partially reversed by treatment with eplerenone. Together this indicates that hypoxia activates the MR and influences thereby lipid and glucose metabolism in hepatocytes. Before hypoxia treatment, the cells were starved for 24 h hours, therefore the natural ligands of the MR - glucocorticoids or mineralocorticoids - should not be present in the medium. Under normoxic conditions eplerenone had no effect on the expression of the respective genes (HepG2: Supplementary figure S9; pRH: Supplementary figure S10). Additionally, dexamethasone (100 nM, Supplementary table S5) under normoxic conditions had no significant effect on the expression of the respective genes, while RU-487 (1 µM, glucocorticoid receptor antagonist) increased the expression of ABCC2 and PDK4 (Supplementary figure S11).

**Fig. 5:**
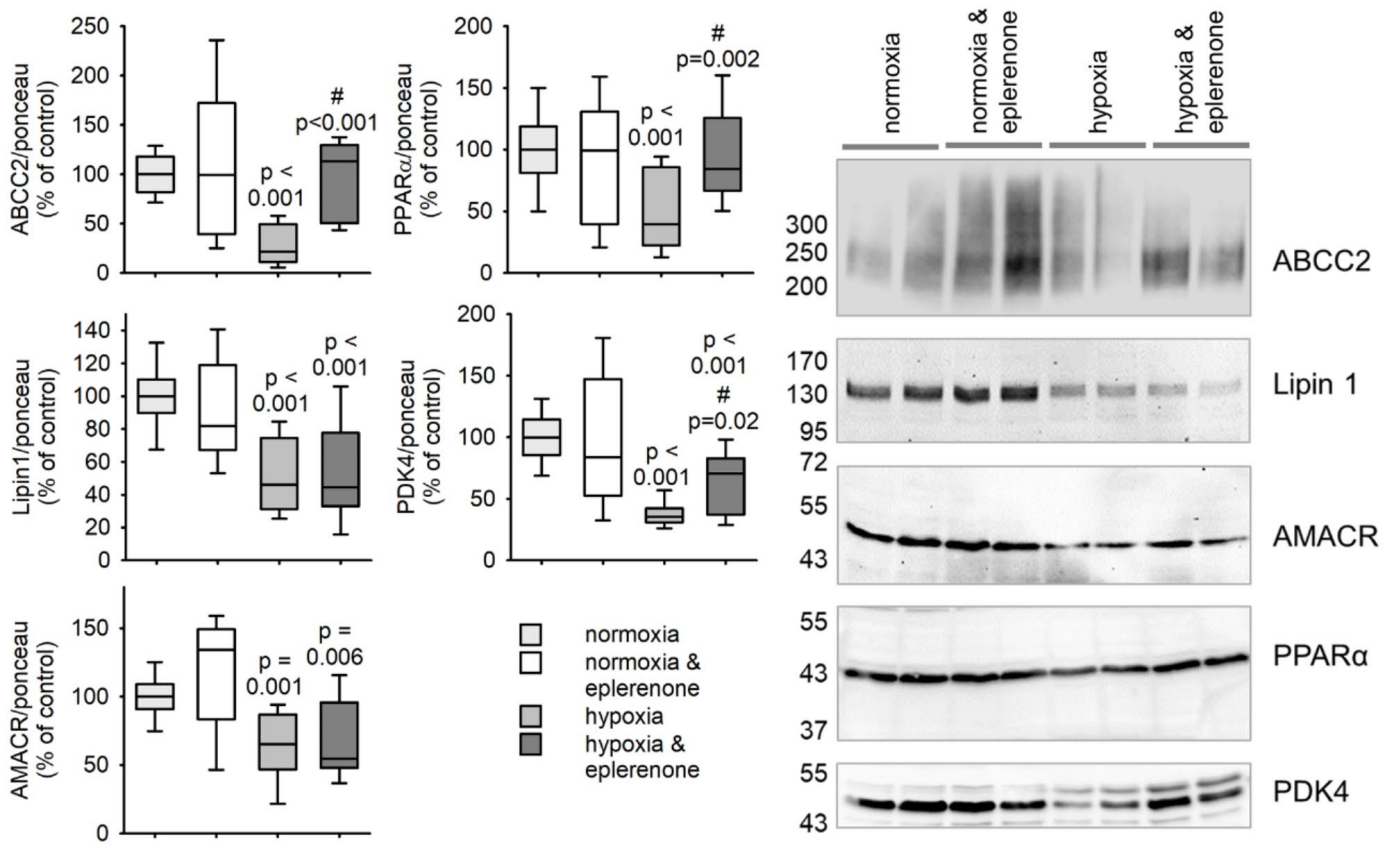
Change in protein expression of representative proteins annotated in the GO term “monocarboxylic acid metabolism” in human HepG2 cell line upon treatment with hypoxia. HepG2 cells were treated for 24 h either under normoxic (16% oxygen) or hypoxic (0.2% oxygen) conditions with or without eplerenone (5µM). *N* = 5 experiments, *n* = 9–10 petri dishes/group, when below 0.05 p-values are given for the comparison vs. normoxia, control. # indcates the p-value for the comparison vs. hypoxia, control

To ensure that the changes in mRNA amount translate into changes in protein content, we performed Western blot analysis (Fig. 5). Eplerenone under normoxic conditions did not alter the protein expression of ABCC2, PPARα, Lipin-1, PDK4 or AMACR. After 24 h of hypoxia, the protein content of all five proteins reduced significantly. This effect was partially inhibited by eplerenone for ABCC2, PPARα and PDK4. Lipin-1 and AMACR protein amount was not increased by eplerenone.

### Under hypoxic conditions MR increases glucose consumption and lipid accumulation in hepatocytes

**Fig. 6:**
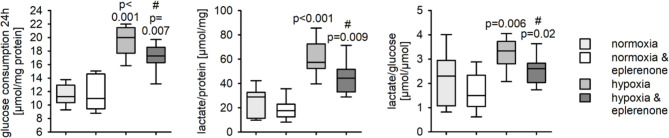
Impact of hypoxia and eplerenone on glucose metabolism in HepG2 cells. HepG2 cells were incubated for 24 h under normoxic (16%) or hypoxic (0.2%) conditions with or without eplerenone (5µM). *N* = 7 experiments, *n* = 13–14 petri dishes/group, p-values are given for the comparison vs. normoxia, control. # indcates the p-value for the comparison vs. hypoxia, control

As the eplerenone regulated genes under hypoxic conditions are related to cellular glucose and lipid metabolism we tested if eplerenone leads to an altered glucose metabolism in HepG2 cells. Hypoxia (24 h, 0.2% oxygen) does not lead to a significantly reduced protein concentration/well (Supplementary figure S12). As demonstrated in Fig. 6 under normoxic conditions, eplerenone has no effect on glucose consumption or lactate production of HepG2 cells over 24 h. As expected, under hypoxic conditions HepG2 cells consume more glucose. This effect is partially attenuated by eplerenone. Under hypoxic conditions acetyl-CoA cannot be used in the TCA cycle for ATP production. Therefore, an increased amount of lactate is produced in the cells. Lactate production is reduced by eplerenone under hypoxic conditions but not under normoxic conditions. From one glucose molecule only two lactate molecules can be produced. Under hypoxic conditions the lactate/glucose ratio increases to values ~ 3.5 (Fig. 6). This might indicate an increased utilization of alternative carbon sources, like glutamate, for energy supply in the HepG2 cells. The effect is attenuated by eplerenone under hypoxic conditions.

**Fig. 7:**
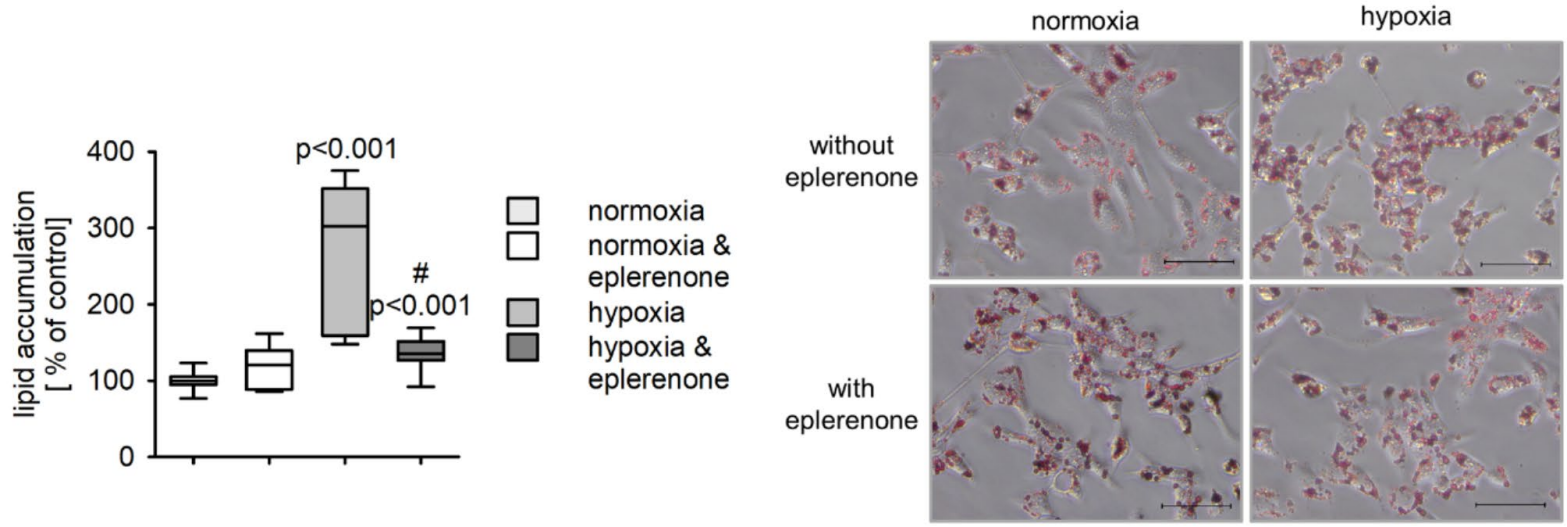
Impact of hypoxia and eplerenone on lipid accumulation in HepG2 cells. HepG2 cells were incubated for 24 h under normoxic (16%) or hypoxic (0.2%) conditions with or without eplerenone (5µM) and subsequently with free fatty acid containing cell culture media. *N* = 6 experiments, *n* = 11–12 petri dishes/group, When below 0.05 p-values are given for the comparison vs. normoxia, control. # indcates the p-value for the comparison vs. hypoxia, control

To test if eplerenone influences the intracellular lipid accumulation in hepatocytes we incubated HepG2 cells for 24 h under hypoxic conditions and subsequently added, under normoxic conditions, free fatty acid containing medium to allow lipid accumulation. While eplerenone under normoxic conditions had no effect on lipid accumulation, increased intracellular lipid accumulation after hypoxia was partially reversed by eplerenone in HepG2 cells (Fig. 7).

### MR effect on oxygen consumption rate, apoptosis and LDH activity in HepG2 cells

**Fig. 8:**
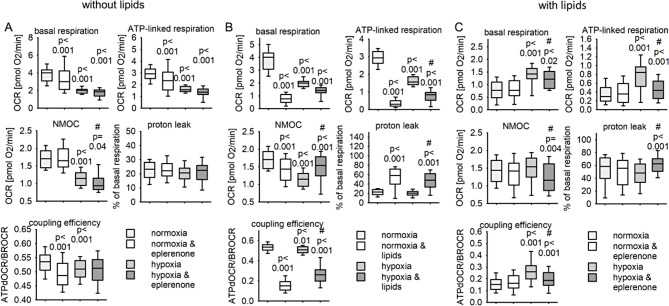
Impact of hypoxia, eplerenone and free fatty acids on mitochondrial oxygen consumption in HepG2 cells. (**A**) HepG2 cells were incubated for 24 h under normoxic (16%) or hypoxic (0.2%) conditions with or without eplerenone (5µM). Oxygen consumption rate was meaured 4 h after reoxygenation. (**B**) HepG2 cells were incubated for 24 h under normoxic (16%) or hypoxic (0.2%) conditions. After this incubation time free fatty acids were added to the supernatant under normoxic (16% O_2_) conditions for additional 4 h. Oxygen consumption rate was measured. (**C**) HepG2 cells were incubated for 24 h under normoxic (16%) or hypoxic (0.2%) conditions with or without eplerenone (5mM). After this incubation time free fatty acids were added to the supernatant under normoxic (16% O_2_) conditions for additional 4 h. Oxygen consumption rate was measured. *N* = 4 experiments, *n* = 56–73 wells/group, p-values are given for the comparison vs. normoxia, control. # indcates the p-value for the comparison vs. hypoxia, control

We analyzed if eplerenone affects mitochondrial function explaining the improved cellular glucose and lipid metabolism in hepatocytes. HepG2 cells were incubated as described before and oxygen consumption rate (OCR) was measured 4 h after oxygen levels were normalized. In cells exposed to normoxia alone, eplerenone reduces basal respiration and ATP-production linked respiration, leading to a reduced coupling efficiency (Fig. 8A). Even four hours after reoxygenation OCR attributable to basal respiration, ATP-production linked oxygen consumption, proton leak and non-mitochondrial oxygen consumption of HepG2 cells is reduced. Eplerenone does not alter the mitochondrial OCR of the reoxygenated cells but leads to an additional reduction of non-mitochondrial oxygen consumption (Fig. 8A).

As MR under hypoxic conditions influences the expression of genes related to glucose and lipid metabolism in hepatocytes we incubated the HepG2 cells after hypoxia treatment with or without lipids for 4 h (Fig. 8B). In normoxia pretreated HepG2 cells addition of lipids per se reduced basal respiration, non-mitochondrial oxygen consumption and ATP-linked oxygen consumption, as reflected by the reduced coupling efficiency (Fig. 8B). When lipids were added to HepG2 cells pretreated with hypoxia than the NMOC was increased. As basal respiration was altered by the previous availability of oxygen, we calculated the percentage of proton leak of the respective basal respiration. Lipids increased the proton leak independent of the previous oxygen tension (Fig. 8B).

Addition of eplerenone was not able to impede the lipid effect in HepG2 cells that were incubated the whole time under normoxic conditions (Fig. 8C). In cells treated with eplerenone and hypoxia lipids still reduced basal respiration, ATP-production linked respiration as well as the non mitochondrial oxygen consumption, leading to a reduced coupling efficiency (Fig. 8C). In summary, we exclude that the beneficial effects of eplerenone on glucose metabolism and lipid accumulation under hypoxic conditions is due to a change of mitochondrial function.

**Fig. 9:**
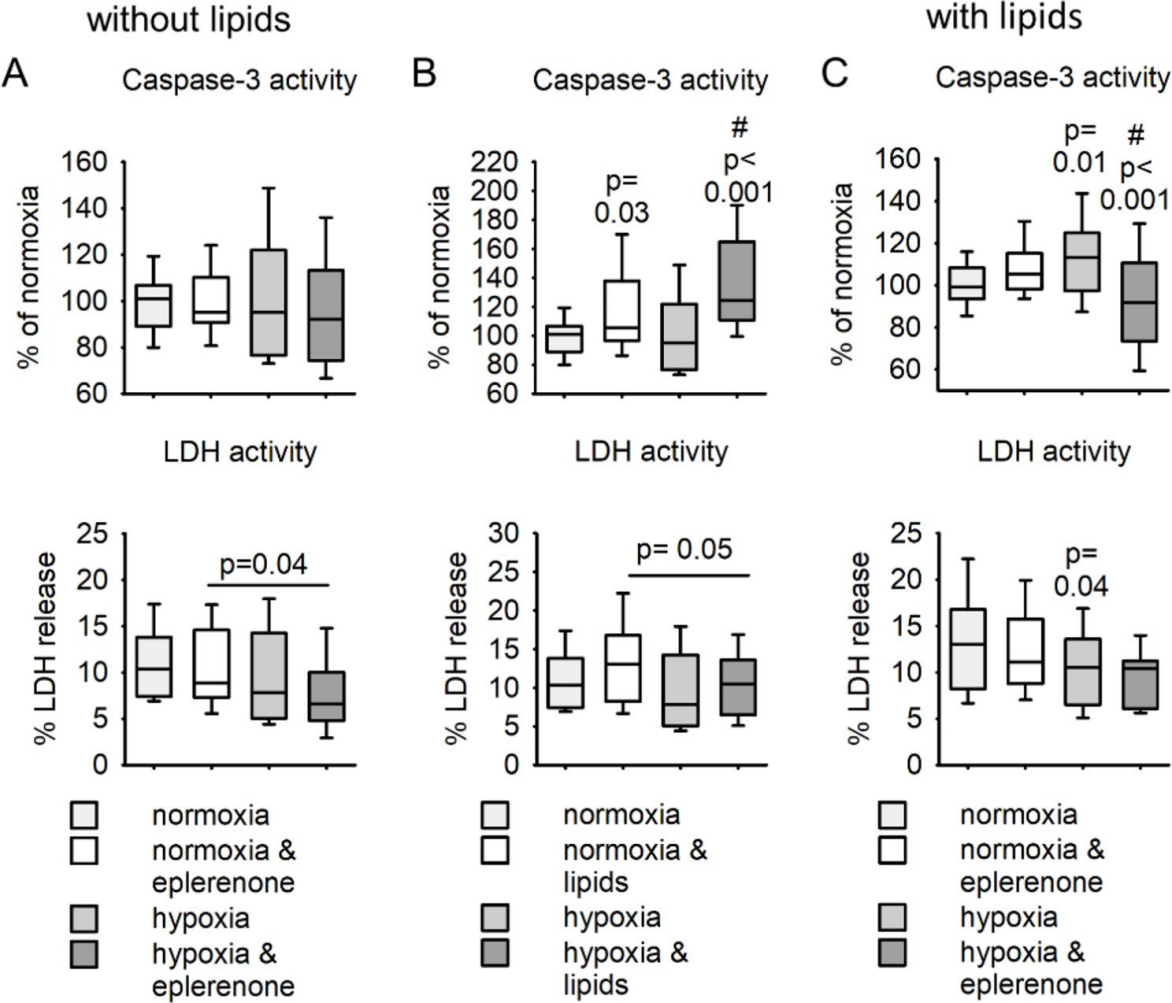
Impact of hypoxia, eplerenone and free fatty acids on apoptosis (caspase-3 activity) or necrosis (LDH activity) in HepG2 cells. HepG2 cells were incubated for 24 h under normoxic (16%) or hypoxic (0.2%) conditions with or without eplerenone (5µM). After this incubation time new media with or without free fatty acids were introduced and the cells were incubated under normoxic (16% O2) conditions for additional 24 h. After this time caspase3 activity, as an indicator of apoptosis, and LDH release in to the media, as an indicator for necrosis were measured and normalized to the protein content. (A) Dependence of the eplerenone effect on oxygen pretreatment in the absence of additional free fatty acids. (B) Effect of free fatty acids either under normoxic or hypoxic pretreatment. (C) Effect of eplerenone after normoxic or hypoxic pretreatment and in the presence of free fatty acids. *N* = 4 experiments, *n* = 24 petri dishes/group, p-values are given for the comparison vs. normoxia, control. # indcates the p-value for the comparison vs. hypoxia, control

Additionally, we analyzed in HepG2 cells if inhibition of MR under normoxic or hypoxic conditions has an impact on apoptosis (caspase-3 activity) or necrosis (LDH release). There was no impact of eplerenone on caspase-3 activity after 24 h of hypoxia or normoxia preincubation in the absence of free fatty acids for additional four hours after reoxygenation (Fig. 9). Addition of free fatty acids for 24 h increased caspase-3 activity in normoxic as well as in reoxygenated HepG2 cells. In cells treated under normoxic conditions alone eplerenone could not impede the effect of lipids on apoptosis. In contrast, in HepG2 cells treated for 24 h under hypoxic conditions before addition of lipids eplerenone inhibited caspase-3 activation. Neither hypoxia, eplerenone nor the addition of lipids had a major effect on the release of LDH into the media. In summary, treatment with eplerenone under hypoxic conditions in the presence of lipids leads to a slight reduction of apoptosis in HepG2 cells.

In summary, our data show that in cirrhosis, hypoxia activates the mineralocorticoid receptor and contributes to alterations in hepatocyte metabolism. Inhibition of the mineralocorticoid receptor under hypoxic conditions abrogated these alterations contributing to a reduced progression of liver deterioration, most probably by the reduction of hepatocyte cell death. Further studies need to investigate the mechanism of mineralocorticoid receptor induced metabolic alterations under hypoxic conditions.

## Discussion

In a former study [16], we demonstrated that application of eplerenone in CCl_4_-treated rats reduced fibrosis in livers. We identified hepatocytes to be the main cell type expressing the MR under physiological conditions. Upon activation the MR translocates to the nucleus and is degraded subsequently. This has been demonstrated in e.g. Hek293, MH1C1, RAW264.7 and bovine distal pulmonary artery smooth muscle cells [16, 48, 49]. Therefore, a reduced protein amount of the MR upon hypoxia in concert with a shift in transcriptional activation upon hypoxia [16] indicates an aldosterone independent activation of the MR. Herein we demonstrate by Western blot in combination with pharmacological inhibition, that the MR is activated by hypoxia in absence of the physiological ligand aldosterone in hepatocytes (HepG2 & pRH). Interestingly, 10 µg LPS or acidosis induce a change in protein size of the MR. To our knowledge there are no data available about the posttranscriptional modifications of the MR due to acidosis or LPS. There are reports about splice variants of the MR [50], but only shorter protein variants are listed. Additionally, several posttranslational modifications of the MR have been reported including phosphorylation, acetylation, ubiquitination, sumoylation and changes induced by nitrosative and oxidative stress [51] leading to an increase in molecular weight. To identify those changes is beyond the scope of this article.

It cannot be excluded that upon hypoxic stimulation, the cells synthesize MR ligands, but to our knowledge a de-novo-synthesis of aldosterone or glucocorticoids by hepatocytes is not described. On the other hand, glucocorticoids as well as mineralocorticoids are mainly metabolized by liver enzymes [52, 53], explaining increased plasma levels in liver diseases. Therefore, the in vivo situation is most probably different to the in vitro situation. In vivo ligand-dependent and ligand-independent effects of the MR might overlap and influence each other. Additionally, one has to take into account that eplerenone might act by binding to the glucocorticoid receptor, progesteron receptor or androgen receptor. According to Garthwaite & McMahon [54] the IC50 or the EC50 of eplerenone for the glucocorticoid receptor or the progesteron receptor is > 100µM, which is significantly higher than the eplerenone concentration we used, making unspecific side effects of eplerenone unlikely. There might be an unspecific side effect of eplerenone on the androgen receptor, as the IC50 is about 5µM, but again our stimulation conditions are lacking exogenous steroids. To our knowledge there is no information about the effect of eplerenone under hypoxic conditions on the afore mentioned steroid hormone receptors.

Treatment of cirrhotic rats with eplerenone reduces liver fibrosis and most probably also portal hypertension [16]. The beneficial effects of MR inhibition have been demonstrated in rodents [16, 19, 55] and in humans [56, 57]. Unfortunately, little is known about the underlying mechanisms. Transcriptomic analysis followed by GO term enrichment analysis of eplerenone treated liver samples from cirrhotic rats was performed and eplerenone sensitive genes were identified. Surprisingly, there was no enrichment of genes related to extracellular matrix formation. The GO terms including upregulated eplerenone sensitive genes pointed to translational processes including the ribosomes (Supplementary figure S4). Unfortunately, we were not able to confirm the transcriptomic changes via qRT-PCR in whole liver samples, isolated pRH or HepG2 cells after hypoxic treatment.

GO term enrichment analysis on the downregulated eplerenone-sensitive genes in cirrhosis indicated an influence of the mineralocorticoid receptor on monocarboxylic acid metabolic processes.

Interestingly, we demonstrated that eplerenone reduces the lactate production in HepG2 cells under normoxic conditions. Besides being a byproduct of glycolysis, lactate has been acknowledged as an effector modulating immune responses [58]. This might indicate that inhibition of MR in vivo reduces lactate production in hepatocytes, thereby reducing the immune stimulatory effects of lactate. Among the eplerenone sensitive upregulated genes PPARα, Lipin-1, AMACR, Abcc2, Cyp1a2 and PDK4 have been identified. In pRH as well as in human HepG2 cells we could confirm that eplerenone reduces the hypoxia induced downregulation of these genes. Nonetheless, we can not exclude that in vivo secondary effects due to tissue alteration or changes in the circulation of the autonomic nervous system contribute to the observed effects by eplerenone treatment.

To understand the implications of the gene expression changes a short description of the function of those proteins is needed. The α-methylacyl-CoA racemase, abbreviated as AMACR or P504S, is involved in the metabolism of branched-chain lipids. These occur as nutritional components, but are also used as drug molecules, e.g. ibuprofen [59]. They are processed and degraded in peroxisomes. AMACR regulates the metabolism of these lipids and drugs by enabling the entry of branched-chain lipids into the peroxisomal and mitochondrial β-oxidation pathways [59]. In addition, AMACR deficiency affects α-oxidation as the action of this enzyme links peroxisomal α- and β-oxidation and is also involved in the β-oxidation of bile acids [60]. The branched-chain lipids, modified by AMACR are known to be high-affinity ligands e.g. for the PPARs [59].

PPARα is a major regulator in the processes of fatty acid uptake, mitochondrial and peroxisomal β-oxidation, ketogenesis, triglyceride turnover and bile acid synthesis while coordinating these processes with peripheral tissue via FGF21 [61]. As a transcription factor it can be activated by free fatty acids and other lipids to enhance lipid clearance in the liver, thereby blunting the effects of high fat diet and other conditions leading to steatosis [61]. PPARα deficiency leads to exaggerated lipid accumulation in the liver [62]. In cardiomyocytes, an aldosterone dependent downregulation of PPARα [63] has been demonstrated. But there is also evidence that MR induced cardiac effects can be at least partially inhibited by activation of PPARs [63, 64]. It has been described that the transcription of PPARα is increased by the glucocorticoid receptor in hepatocytes under normoxic conditions, but not by the MR [65]. Additionally, PPARα and GR together lead to NFκB-transcriptional repression [62]. Unfortunately, little is known about the interplay of PPARα and the MR under hypoxic conditions. To elucidate this further studies are needed to fully understand their potential effects.

The function of Pyruvate dehydrogenase kinase 4 (PDK4) has been reviewed extensively by Sugden & Holness [66]. PDK4 reduces the activity of the pyruvate dehydrogenase complex (PDC) [66]. This enzyme complex links the glycolytic degradation of glucose to the TCA cycle by the rate-limiting and physiologically irreversible oxidative decarboxylation of pyruvate [66]. One of the factors influencing the activity of PDK4 is the NADH/NAD^+^ concentration ratio generated by mitochondrial β-oxidation. This contributed to the hypothesis that PDK4 is a ‘lipid-status’ responsive PDK isoform, facilitating FA oxidation by ‘sparing’ pyruvate for oxaloacetate formation [66]. In addition, up-regulation of PDK4 prevents lipid accumulation in the cytoplasm [66]. At the moment an interaction of the MR with PDK4 has not been reported. Well recognized is the impact of glucocorticoids on PDK4. In liver cell lines dexamethasone increases the expression of PDK4 [67] and PPARα is required for the effect of glucocorticoids to induce hepatic insulin resistance [66, 68].

In summary, the altered expression of these genes indicates a disturbed cellular lipid and glucose homeostasis enabling an increase in cytosolic lipid content presumably due to reduced lipid utilization. Consequently, we analyzed glucose consumption and lipid accumulation in HepG2 cells under hypoxic conditions with and without addition of eplerenone. Under hypoxic conditions, glucose consumption and lactate production were increased, indicating an increase of glycolysis. Interestingly, already under normoxic conditions glycolysis seems to contribute substantially to the energy homeostasis of HepG2 cells, as the lactate/glucose ratio under normoxic conditions is above two. This ratio increases even more under hypoxic conditions. This lactate is produced presumably from amino acids in the cell culture medium (about ~ 7.5 mmol/L glucogenic amino acids). The conclusion can be drawn that under normoxic conditions about 10% of the lactate must be produced by metabolizing other carbon sources. Under hypoxic conditions the metabolization of alternative carbon sources seems to increase, beside the increased glucose consumption. And, under hypoxic conditions eplerenone seems to reduce both.

Disturbances of hepatocyte metabolism often induce an accumulation of intracellular lipid droplets [69] and oxygen is essentially needed for β-oxidation of lipids. The accumulation of lipids in the cytosol might lead to an increase in lipotoxic species and thereby to endoplasmic reticulum stress and oxidative stress (70). We therefore aimed to analyze if hypoxia leads to lipid accumulation in HepG2 cells and if this can be inhibited by eplerenone. To achieve this, the tissue culture medium was supplemented with exogenous lipids and human albumin after hypoxia treatment. Hypoxic conditions enable HepG2 cells to accumulate lipids and this is reduced when eplerenone is present. Whether these fat accumulations are due to reduced utilization under hypoxic conditions or due to de-novo-synthesis of lipids needs to be determined in further experiments. It is discussed that the generation of lipid droplets could be a protective mechanism for the cells, since the metabolism of fats requires more oxygen than the metabolism of glucose [66]. The changes in PPARα, PDK4 and AMACR by eplerenone would favor the utilization of lipids either in the mitochondria or in the peroxisomes. Our results demonstrate, that eplerenone most probably does not induce these alterations in glucose and lipid metabolism by changing the oxygen consumption rate of the mitochondria in HepG2 cells. If eplerenone leads to an increased utilization of carbon sources in the peroxisomes will be the focus of future studies.

Interestingly, we did see that eplerenone reduces the activation of caspase-3 or the release of LDH under hypoxic conditions, which can not be observed under normoxic conditions, this could also indicate that eplerenone via inhibition of the MR in hepatocytes and thereby reduced cell death leads to a reduced activation of hepatic stellate cells. But this has to be evaluated in future experiments.

Herein we demonstrate, that inhibition of the MR by eplerenone - but not inhibition of the GR by RU-486 - reverses at least partially the hypoxia induced changes in the identified, downregulated genes. We hypothesize that the change in expression e.g. of PPARα is leading to a change in hepatocyte metabolism and perhaps to a change in hepatocyte crosstalk with other cells, most probably hepatic stellate cells or sinusoidal endothelial cells. Additionally, eplerenone under hypoxic conditions reduces the apoptosis of hepatocytes. Alltogether this would increase the progression of fibrosis and liver deterioration. One has to mention that in vivo the situation might be more complicated, as the ligands for the mineralocorticoid receptor aldosterone and corticosterone are present. Therefore ligand-dependent and –independent effects most probably occur at the same time and influence each other. Additionally, changes in other tissues than the liver might contribute to the effect of eplerenone on liver cirrhosis.

## Conclusions

From our findings and the literature, we propose the following pathogenic mechanism: In early stages of chronic liver disease the hypoxic areas throughout the liver increase and cause an activation of the MR in hepatocytes independent of its endogenous ligand aldosterone. This changes the expression of several genes related to energy homeostasis and consequently to cell stress related pathways contributing to hepatocyte death and deterioration of liver function. At the moment MR antagonists are given to patients with cirrhosis in decompensated stage to induce diuresis and reduce ascites. We postulate, that administering MR antagonists, like eplerenone, at the initial stage or compensated stage (i.e. cACLD), the hypoxia-induced, ligand-independent MR activation can be stopped. Thereby inhibiting liver deterioration before a “point of no return”. This could, potentially, increase liver healing and reduce health costs. With the presented data, human patient studies analyzing the outcome in patients with cACLD should be taken into account.

## Electronic supplementary material

Below is the link to the electronic supplementary material.

Supplementary Material 1

Supplementary Material 2

Supplementary Material 3

Supplementary Material 4

## Data Availability

RNA sequencing data (raw and processed) on which is based this study have been deposited at GEO and are publicly available as of the date of publication under the accession number GSE255673. Further data and materials that support the findings of this study are available from the corresponding authors upon reasonable request.

## Abbreviations

ABCC2: Multidrug resistance-associated protein 2 /ATP-binding cassette/ MRP2
AMACR: α-Methylacyl-CoA racemase
α: SMA-alpha-smooth muscle actin
ATP: Adenosine triphosphate
BCA: Bicinchoninic acid
BW: Body weight
cACLD: compensated advanced chronic liver disease
CCl_4_: Carbon tetrachloride
COL1A1: Collagen type I alpha 1
CTGF: Connective tissue growth factor
CYP1a2: Cytochrome P450 Family 1 Subfamily A Member 2
DEG: Differentially expressed genes
DNase: Deoxyribonuclease
ECL: Enhanced chemiluminescence
FCS: Fetal calf serum
FDR: False discovery rate
Fn: 1-Fibronectin 1
FPM: fragments per million
GO: Gene ontology
GRE: Glucocorticoid response element
GSEA: Gene set enrichment analysis
HepG2: Human hepatocyte cell line
HEPES − 4: (2-hydroxyethyl)-1-piperazineethanesulfonic acid
HGM: Hepatocyte growth medium
HPRT: Hypoxanthine-guanine phosphoribosyltransferase
IgG: Immunoglobulin G
LDH: lactate dehydrogenase
LPS: Lipopolysaccharide
MCP: 1-Monocyte Chemoattractant Protein-1
MES: Morpholinoethanesulfonic acid
MR: mineralocorticoid receptor
NAD+/NADH: nicotinamide adenine dinucleotide
NADP+/NADPH: nicotinamide adenine dinucleotide phosphate
NIH: National Institutes of Health
PAI1: Plasminogen activator inhibitor-1
PBS: Phosphate buffered saline
PDC: Pyruvate dehydrogenase complex
PDK4: Pyruvate dehydrogenase lipoamide kinase isozyme 4 sub-family C member 2
PPARα: Peroxisome proliferator-activated receptor alpha
pRH: Primary rat hepatocytes
QK1: KH Domain Containing RNA Binding
qRT: PCR-Real-Time Quantitative Reverse Transcription Polymerase chain reaction
RAAS: Renin-angiotensin-aldosterone system
RIN: RNA Integrity Number
RNA: Ribonucleic acid
RPMI: Roswell Park Memorial Institute
RPS: Ribosomal proteins
RT: Reverse transcription
SDS: sodium dodecyl sulfate
SDS: PAGE-Sodium dodecyl-sulfate polyacrylamide gel electrophoresis
SEM: Standard error of mean
SGK: 1-Serum/glucocorticoid regulated kinase 1
TCA cycle: Tricarboxylic acid cycle
TMM: Trimmed mean of M value
TNFα: Tumor necrosis factor alpha
Tris: HCl-Tris (hydroxymethyl)aminomethane hydrochloride
WB: Western blot
