# Supplementary material for: Eplerenone, a mineralocorticoid receptor inhibitor, reduces cirrhosis associated changes of hepatocyte glucose and lipid metabolism
Source: Cell Commun Signal. 2024 Dec 20;22:614. doi: 10.1186/s12964-024-01991-2 (PMC11660827; doi:10.1186/s12964-024-01991-2)
Supplement: Supplementary file 1 — Supplementary Material 1 [file 12964_2024_1991_MOESM1_ESM.pdf]

In this part the original Western Blot pictures are summarized

Western blots for figure 1A

Cell type: primary rat hepatocytes, isolated on 04.09.2020
target protein: mineralocorticoid receptor **MW:** ~107kDa
WB date: 11.11.2020 **Marker :** PageRuler (Thermo Fisher Scientific)
Incubation: normoxia, 1µg/ml LPS, 10µg/ml LPS, hypoxia **duration:** 24h

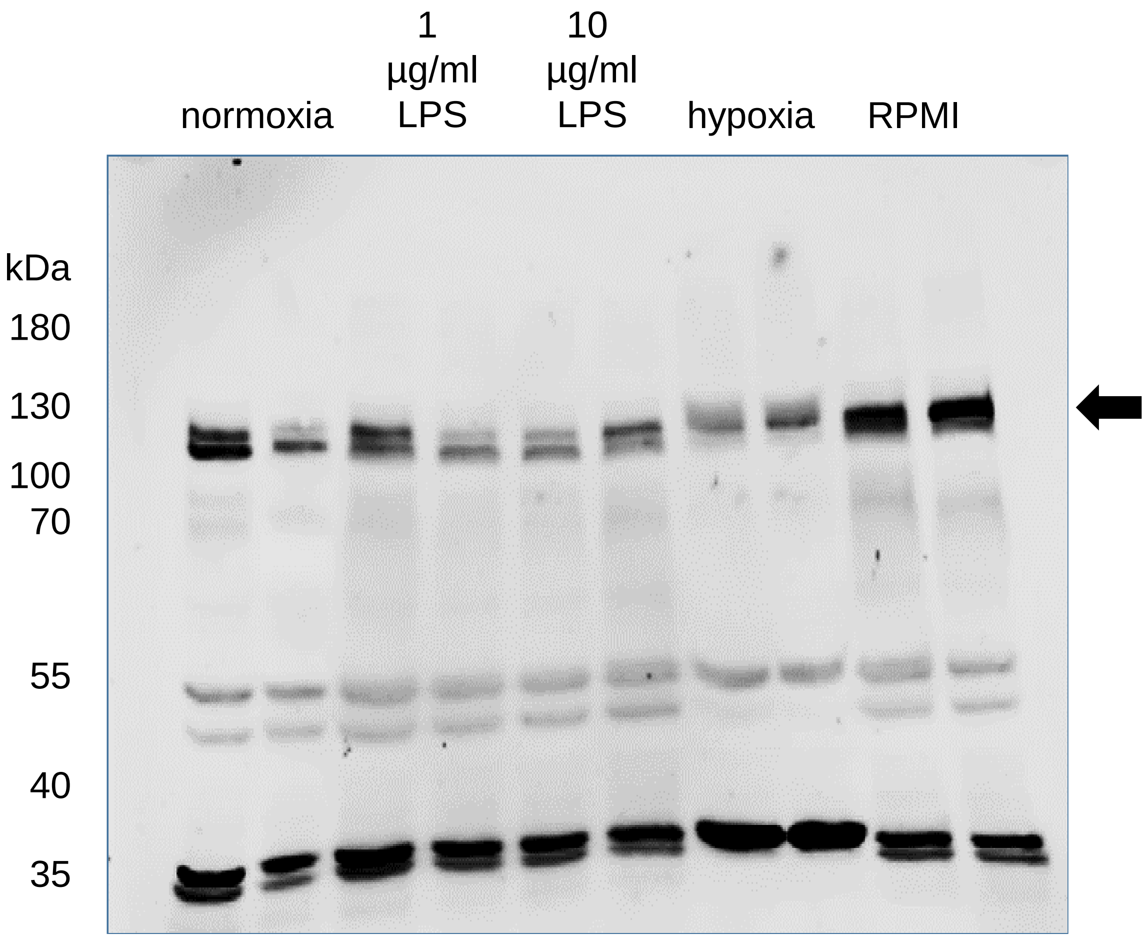

1. AB: MR (1:500) (GS 1-18)105-S
2. AB: anti-Mouse (CellSignaling) 1:1000

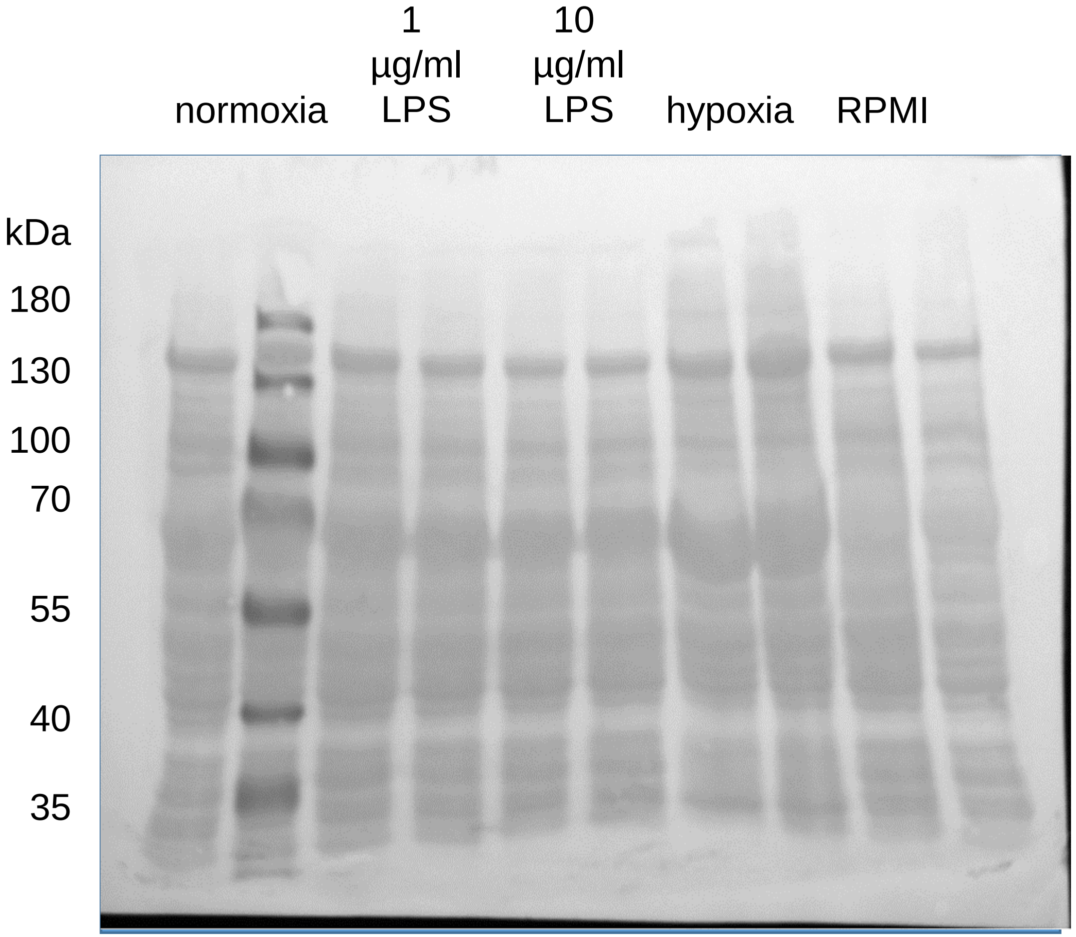

Ponceau

Cell type: primary rat hepatocytes, isolated on 14.09.2020
target protein: mineralocorticoid receptor **MW:** ~107kDa
WB date: 27.02.2023 **Marker :** PageRuler (Thermo Fisher Scientific)
Incubation: normoxia, 1µg/ml LPS, 10µg/ml LPS, hypoxia **duration:** 24h

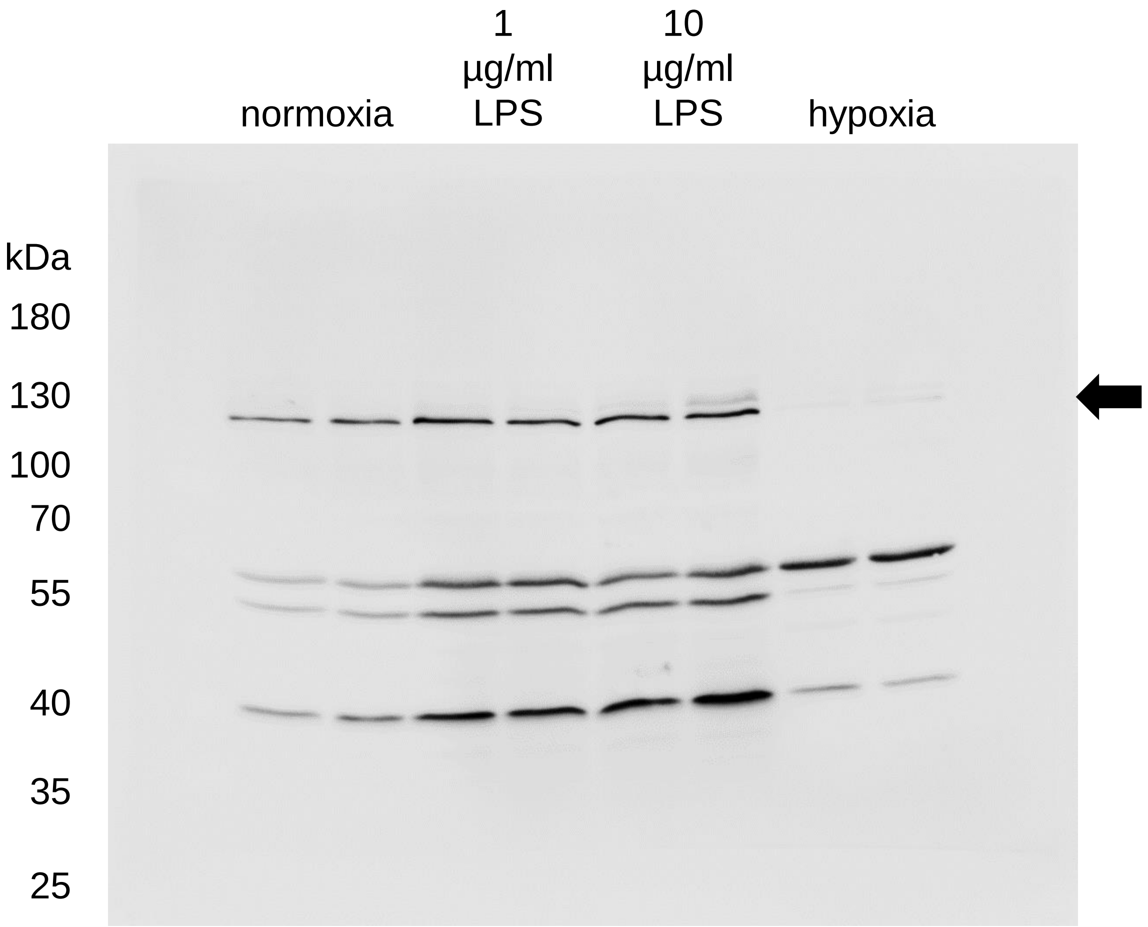

1. AB: MR (1:500) (GS 1-18)105-S
2. AB: anti-Mouse (CellSignaling) 1:1000

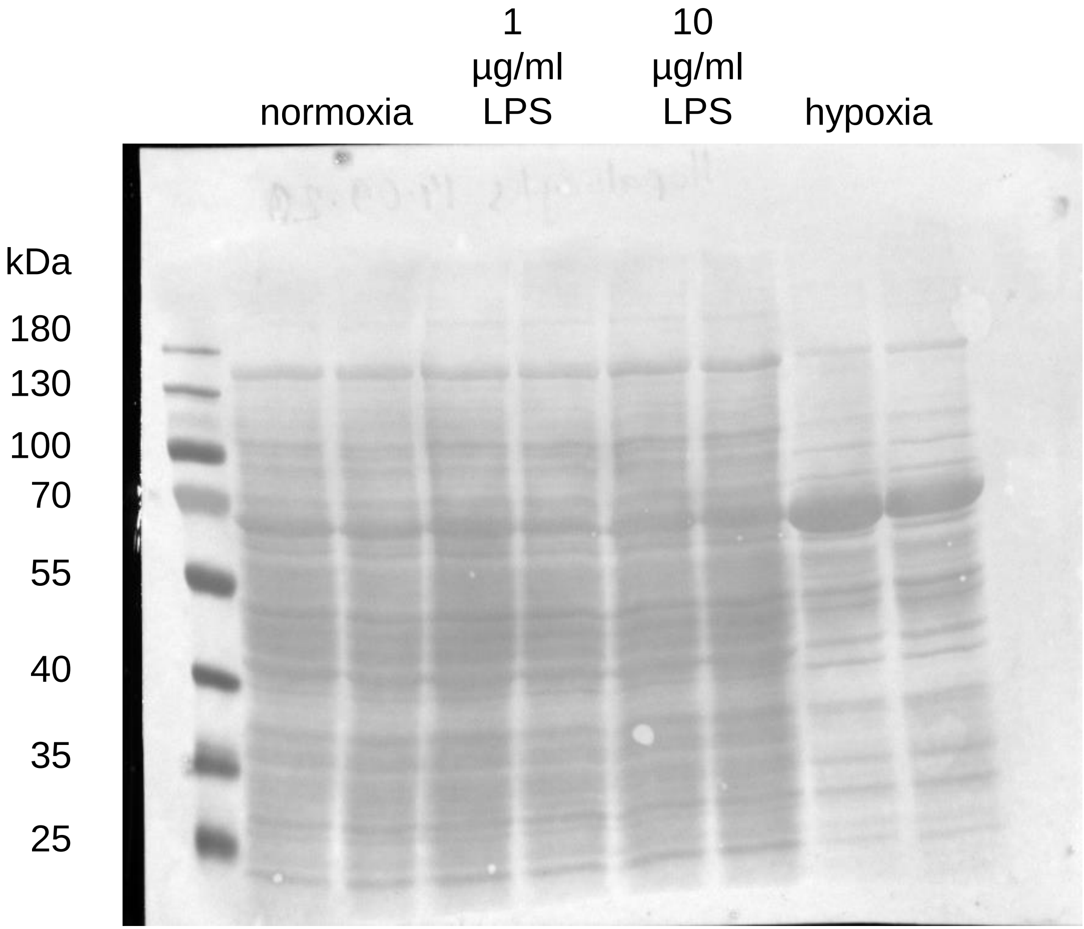

Ponceau

cell type: primary rat hepatocytes, isolated on 04.09.2020 & 14.09.2020
target protein: mineralocorticoid receptor **MW:** ~107kDa
WB date: 27.02.2023 **marker :** PageRuler (Thermo Fisher Scientific)
incubation: normoxia, acidosis **duration:** 24h

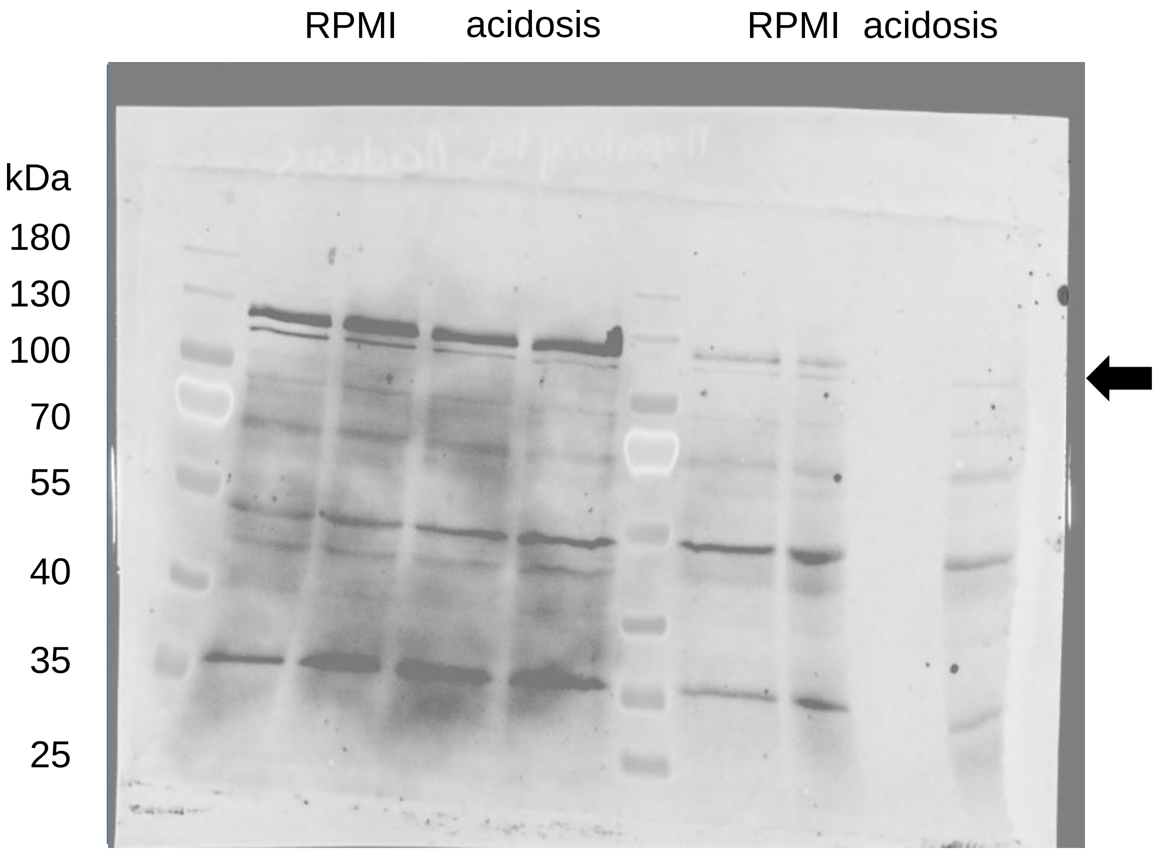

1. AB: MR (1:500) (GS 1-18)105-S
2. AB: anti-Mouse (CellSignaling) 1:1000

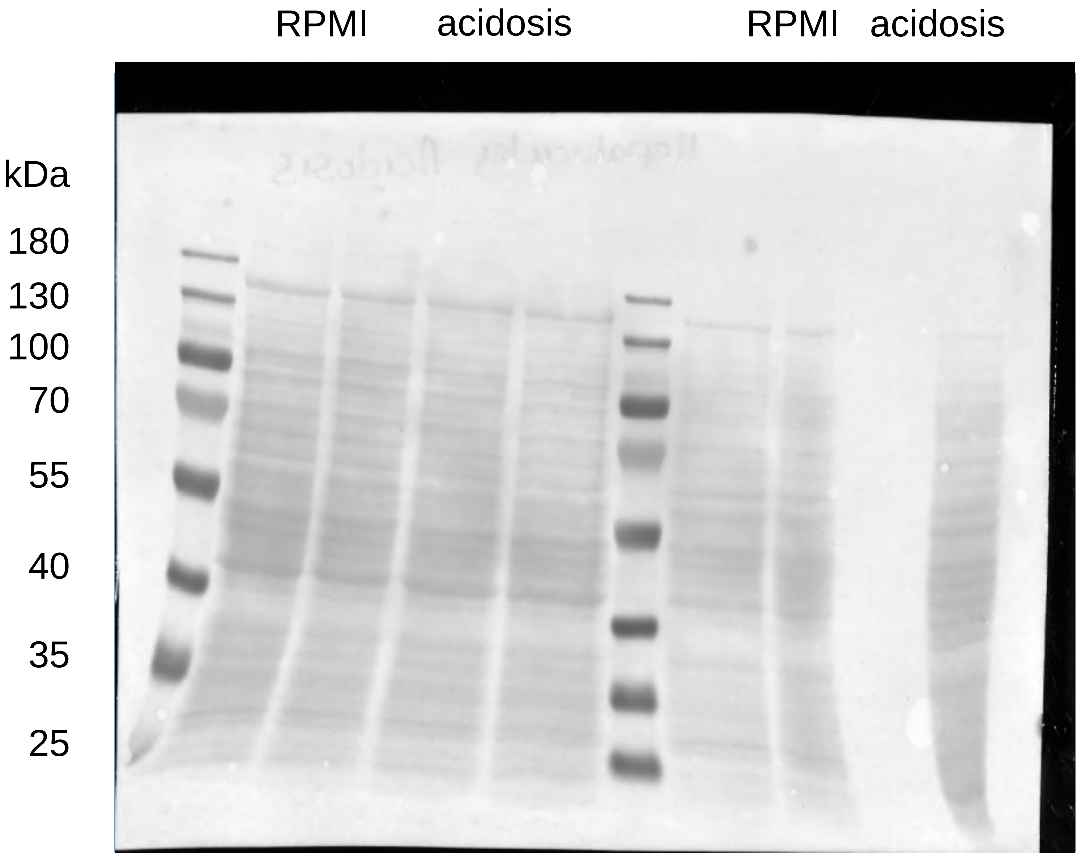

Ponceau

cell type: primary rat hepatocytes, isolated on 18.09.2020
target protein: mineralocorticoid receptor **MW:** ~107kDa
WB date: 10.10.2020 **Marker :** PageRuler (Thermo Fisher Scientific)
incubation: normoxia, 1µg/ml LPS, 10µg/ml LPS, acidosis, hypoxia **duration:** 24h

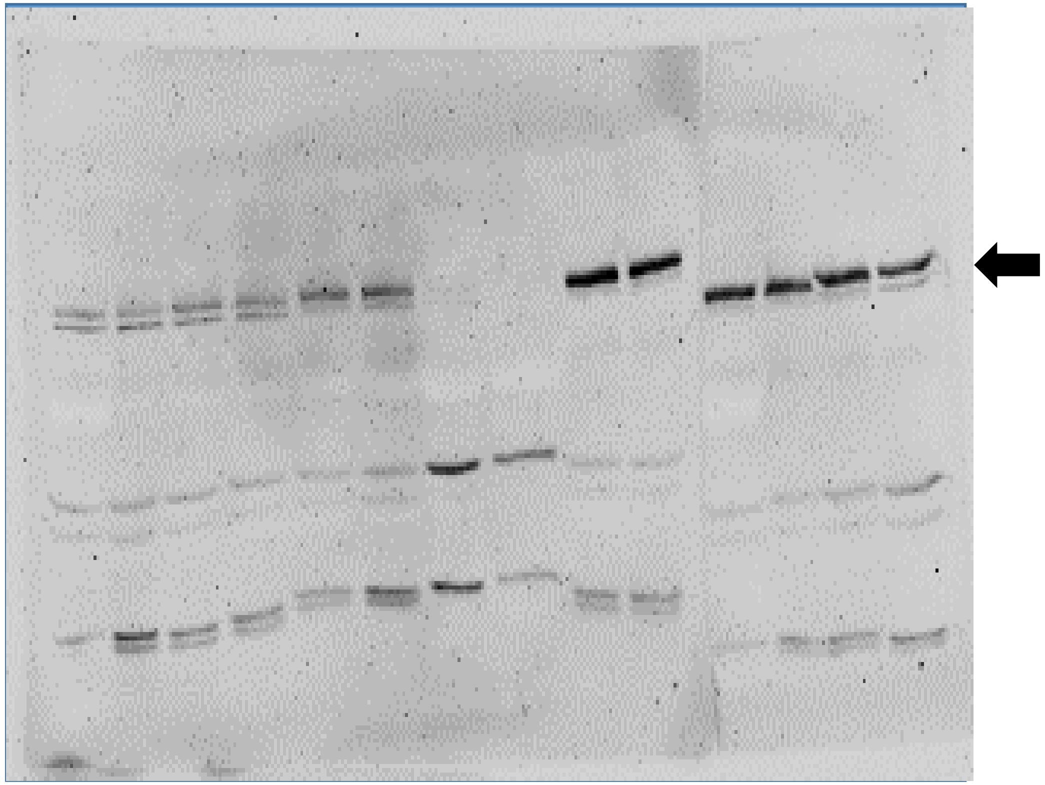

1. AB: MR (1:500) (GS 1-18)105-S
2. AB: anti-Mouse (CellSignaling) 1:1000

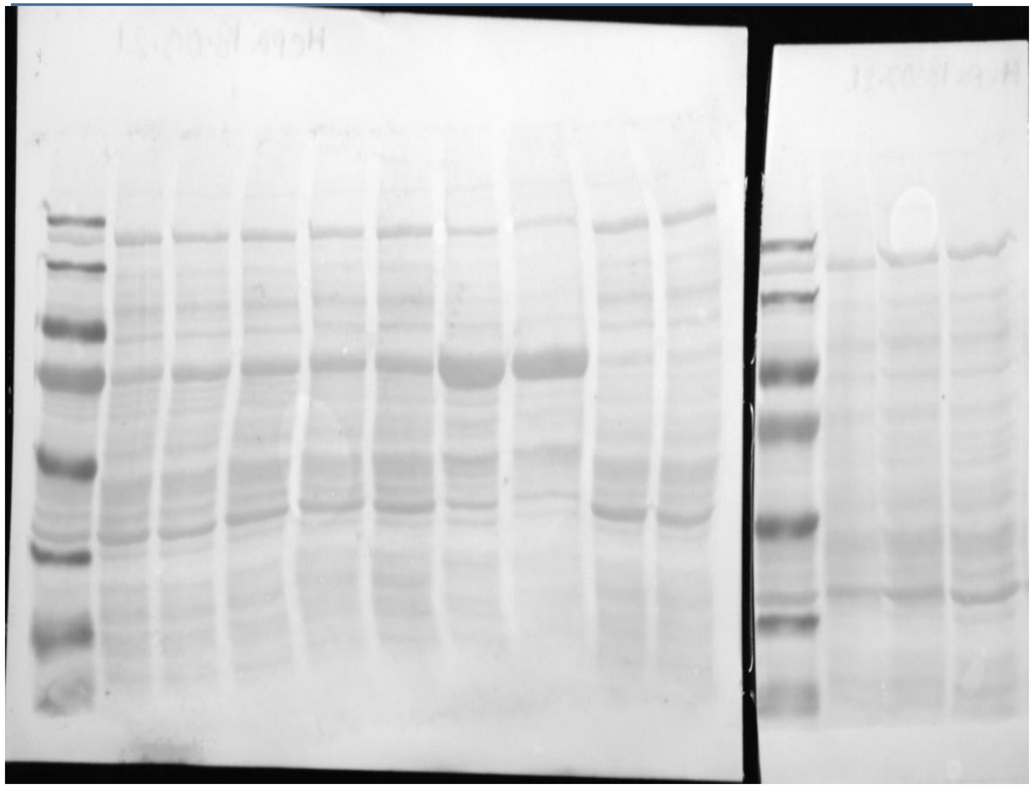

Ponceau

Cell type: primary rat hepatocytes, isolated on 01.10.2020
target protein: mineralocorticoid receptor **MW:** ~107kDa
WB date: 27.02.2023 **Marker :** PageRuler (Thermo Fisher Scientific)
Incubation: normoxia, 1µg/ml LPS, 10µg/ml LPS, hypoxia **duration:** 24h

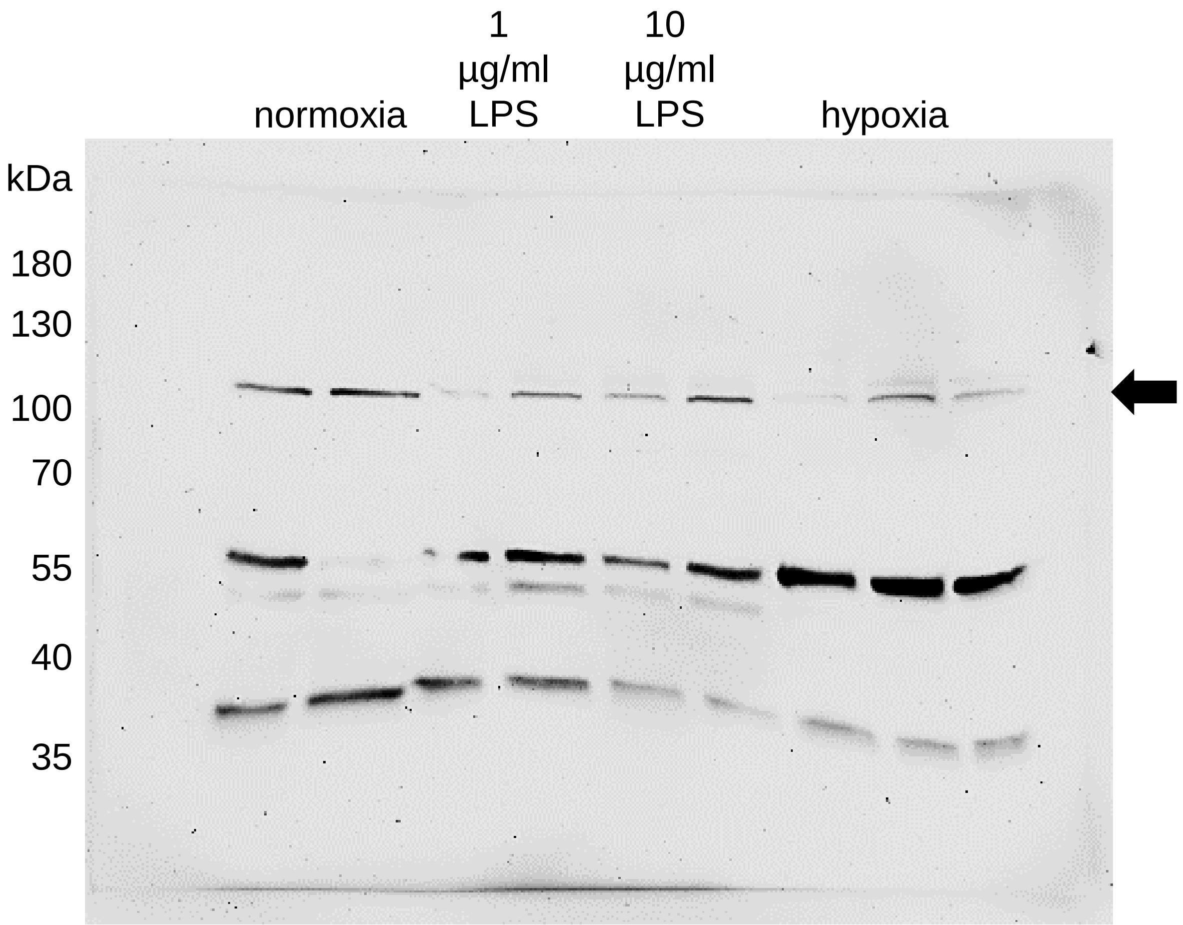

1. AB: MR (1:500) (GS 1-18)105-S
2. AB: anti-Mouse (CellSignaling) 1:1000

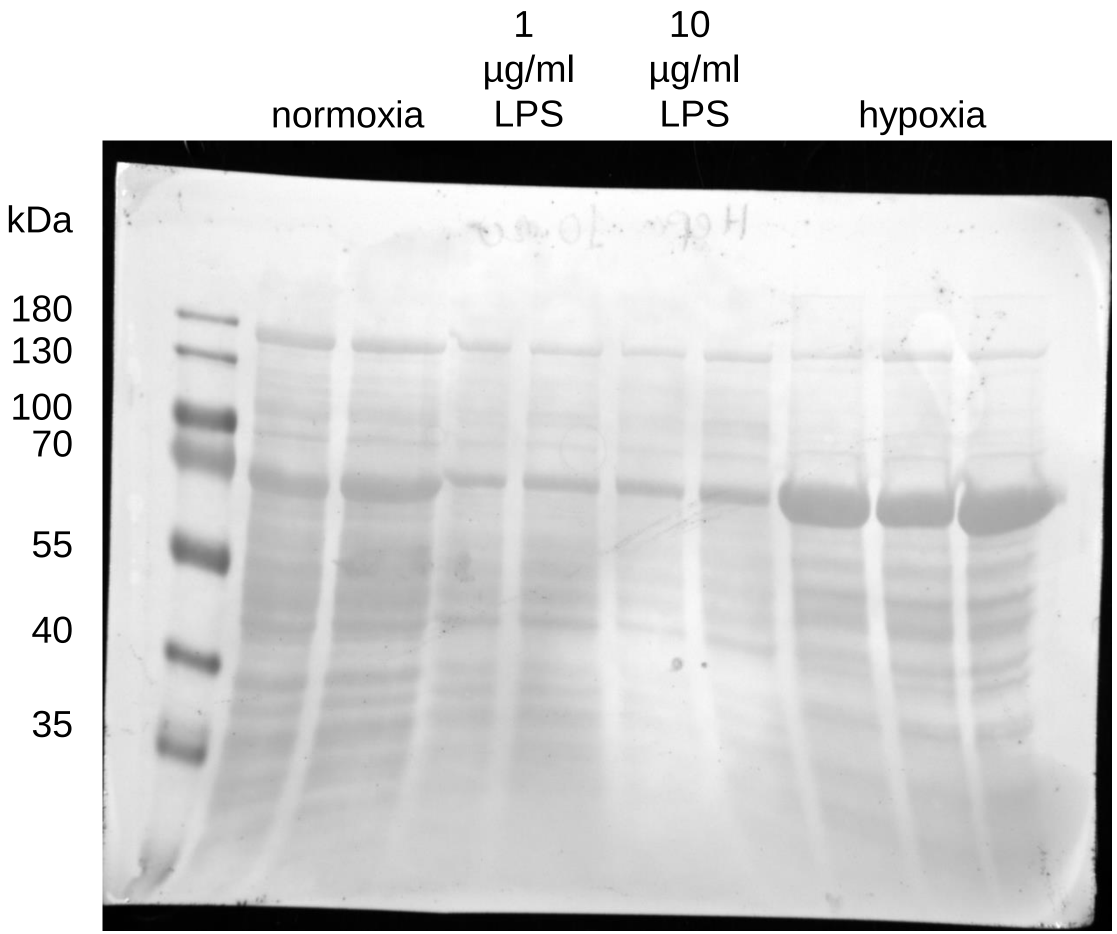

Ponceau

cell type: primary rat hepatocytes, isolated on 15.10.2020
target protein: mineralocorticoid receptor **MW:** ~107kDa
WB date: 17.03.2023 **Marker :** PageRuler (Thermo Fisher Scientific)
incubation: normoxia, 1µg/ml LPS, 10µg/ml LPS, hypoxia **duration:** 24h

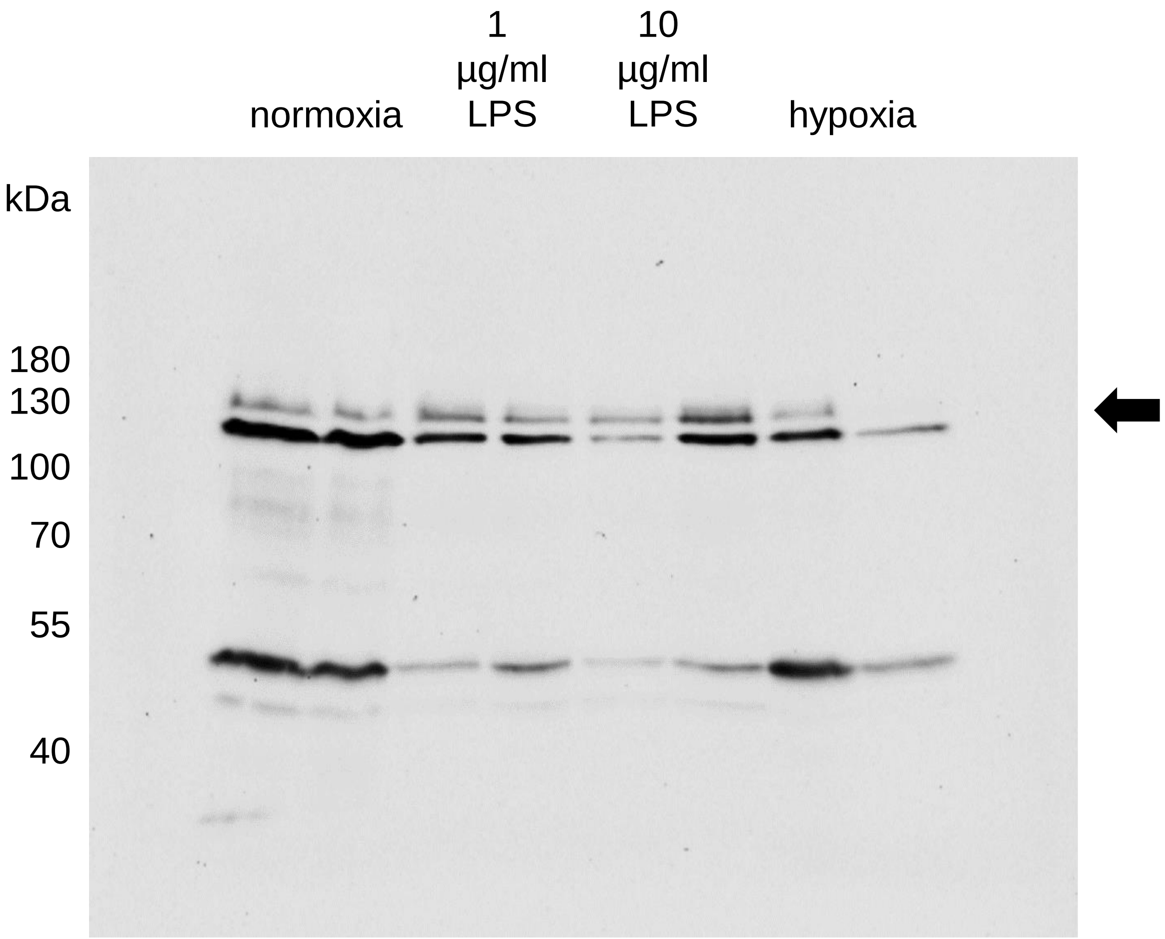

1. AB: MR (1:500) (GS 1-18)105-S
2. AB: anti-Mouse (CellSignaling) 1:1000

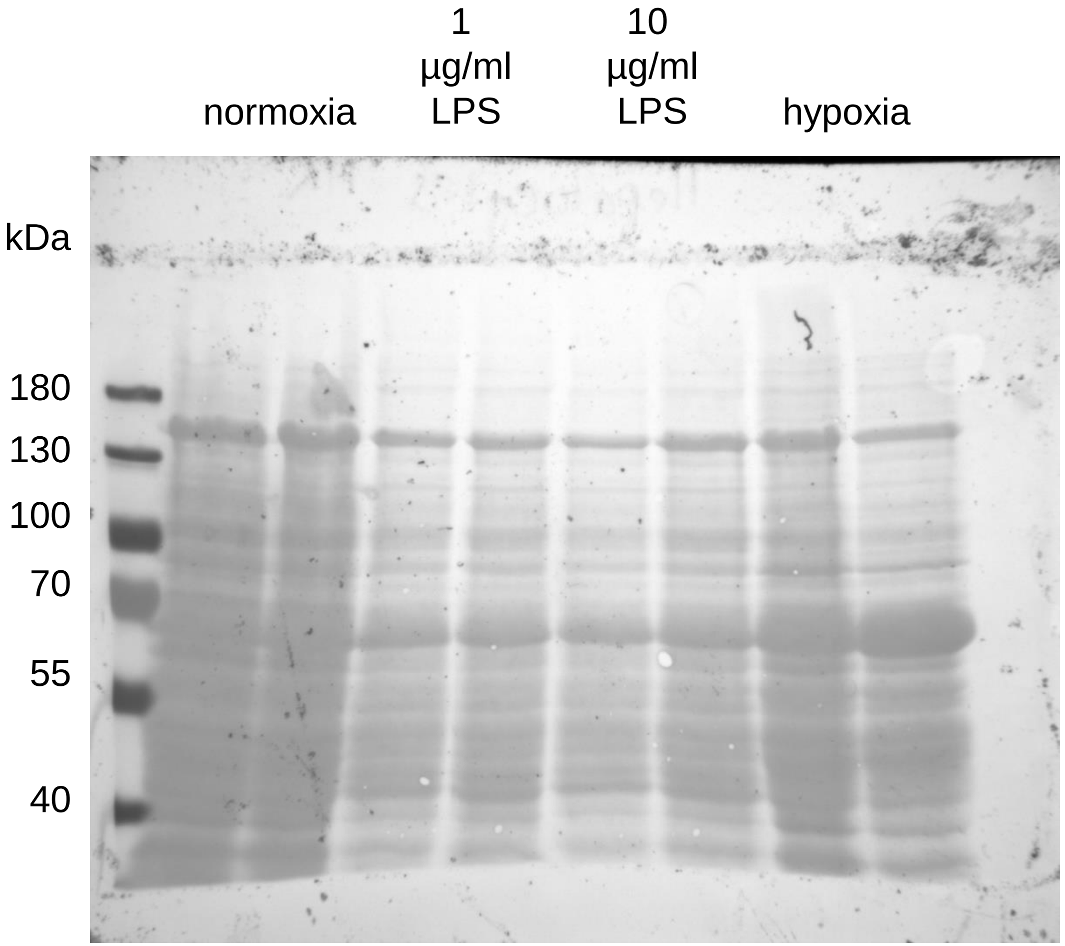

Ponceau

Cell type: primary rat hepatocytes, isolated on 01.10.2020 & 15.10.2020
target protein: mineralocorticoid receptor **MW:** ~107kDa
WB date: 17.03.2023 **Marker :** PageRuler (Thermo Fisher Scientific)
Incubation: normoxia, acidosis, hypoxia **duration:** 24h

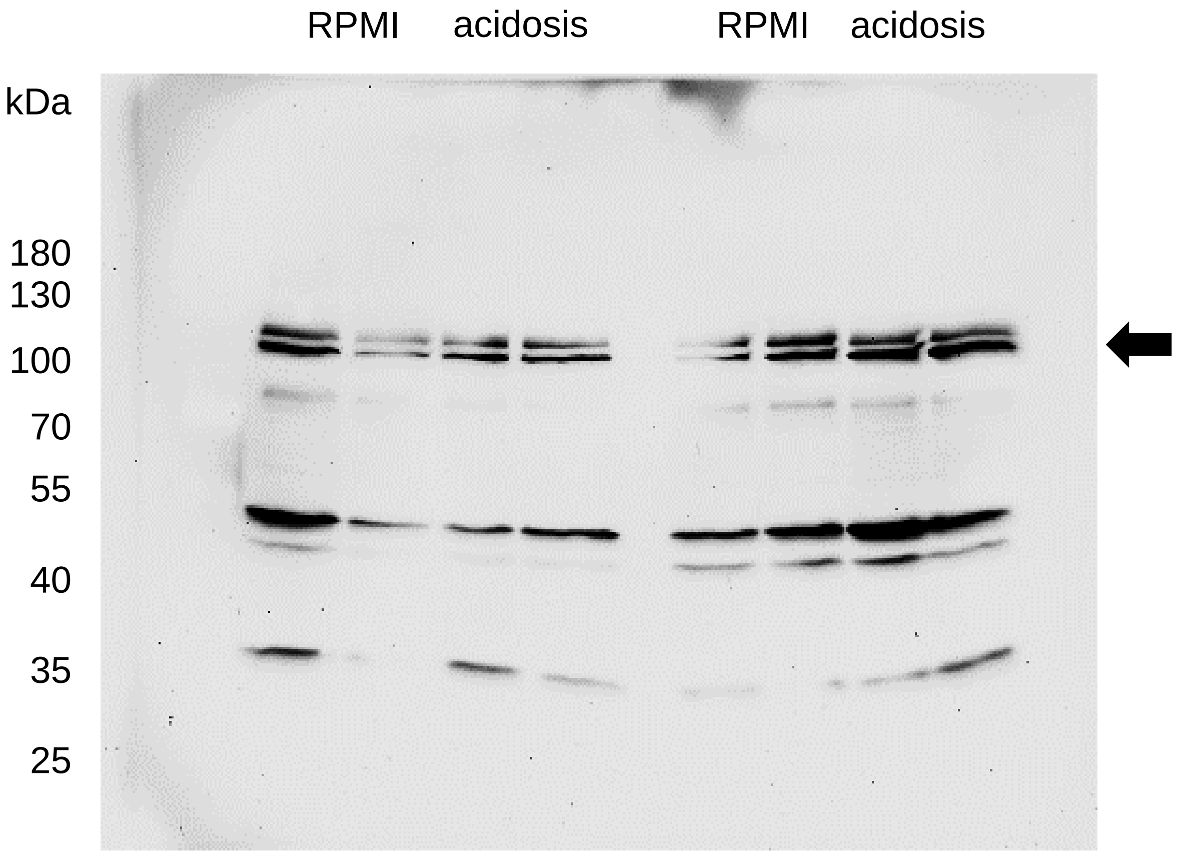

1. AB: MR (1:500) (GS 1-18)105-S
2. AB: anti-Mouse (CellSignaling) 1:1000

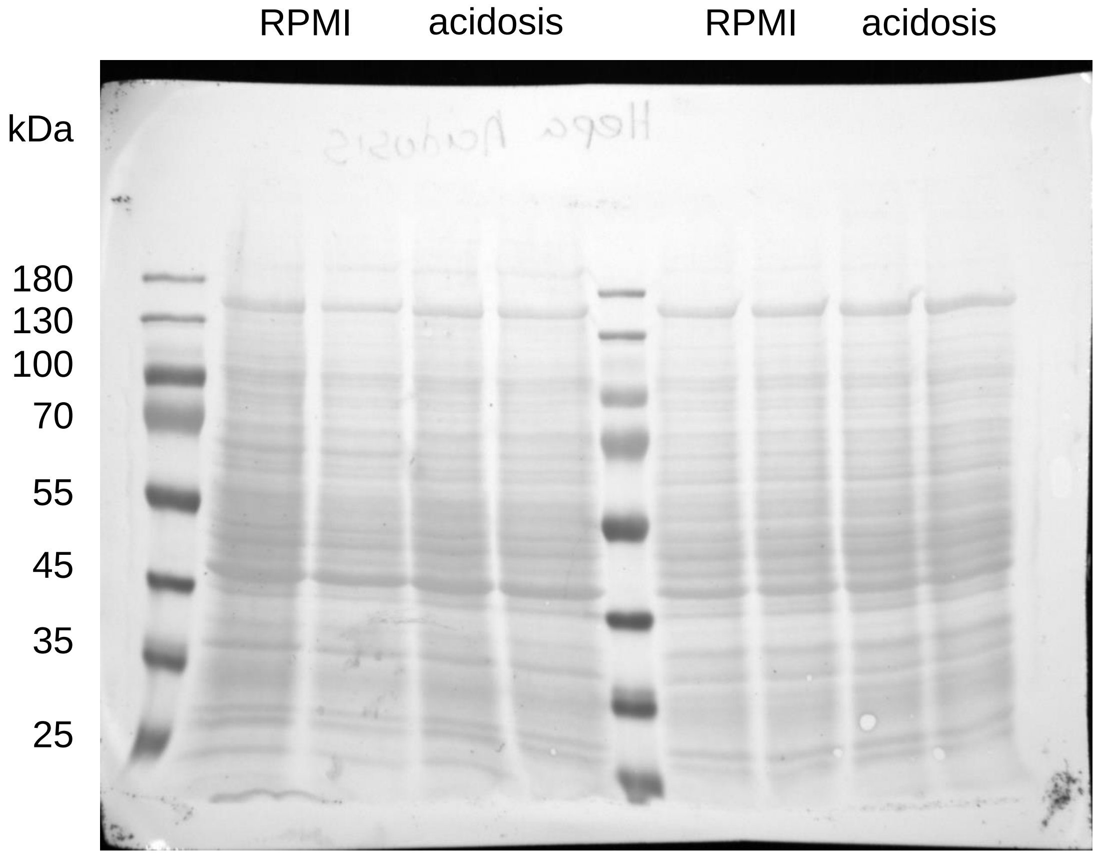

Ponceau

Western blots for figure 1B

cell type: HepG2, passage x+25

target protein: mineralocorticoid receptor

WB date: 10.03.2022

incubation: normoxia, 1µg/ml LPS, 10µg/ml LPS, acidosis, hypoxia

positive control: myc-tagged MR (~ 110 kDa)

date of experiment: 07.03.2022

MW: ~107kDa

Marker : PageRuler (Thermo Fisher Scientific)

duration: 24h

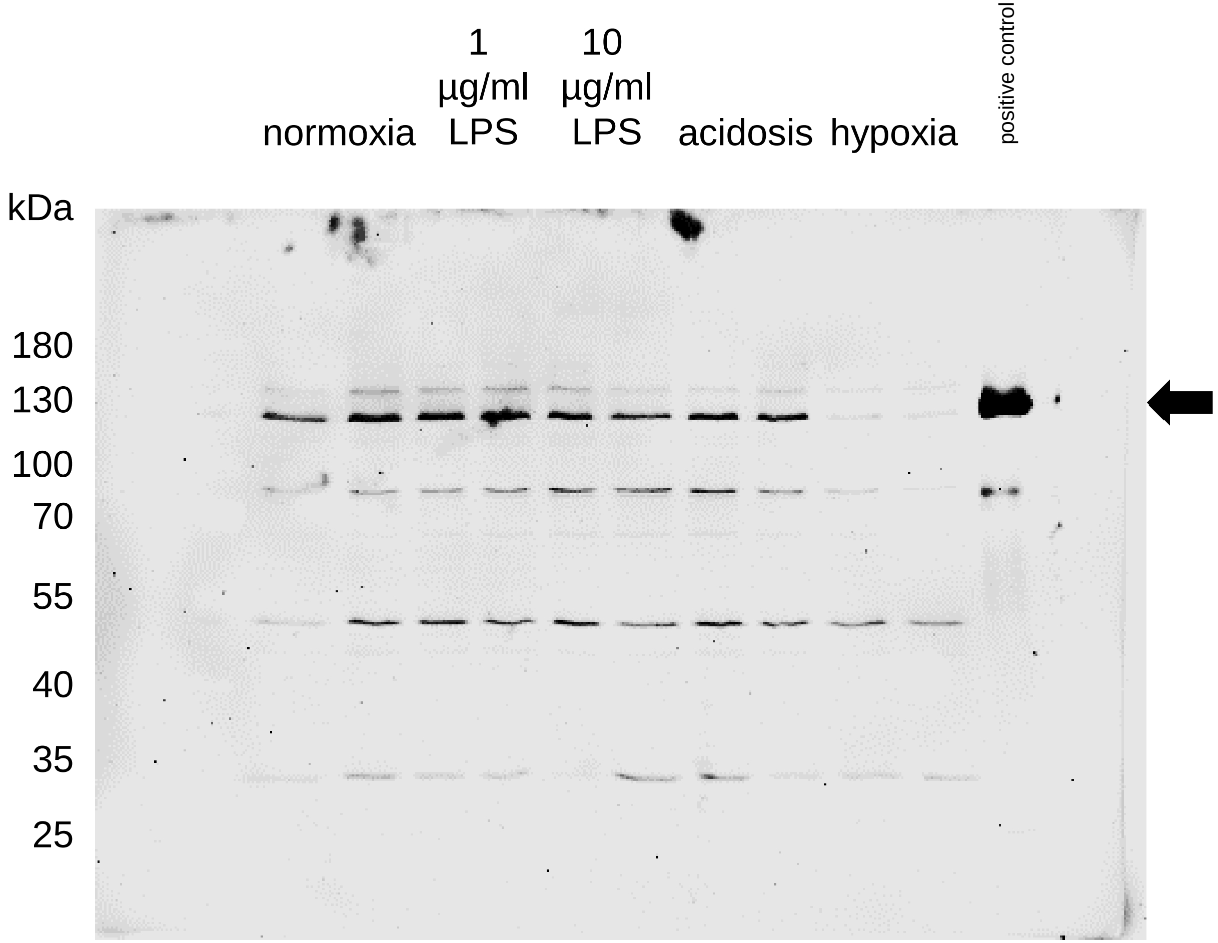

1. AB: MR (1:500) (GS 1-18)105-S
2. AB: anti-Mouse (CellSignaling) 1:1000

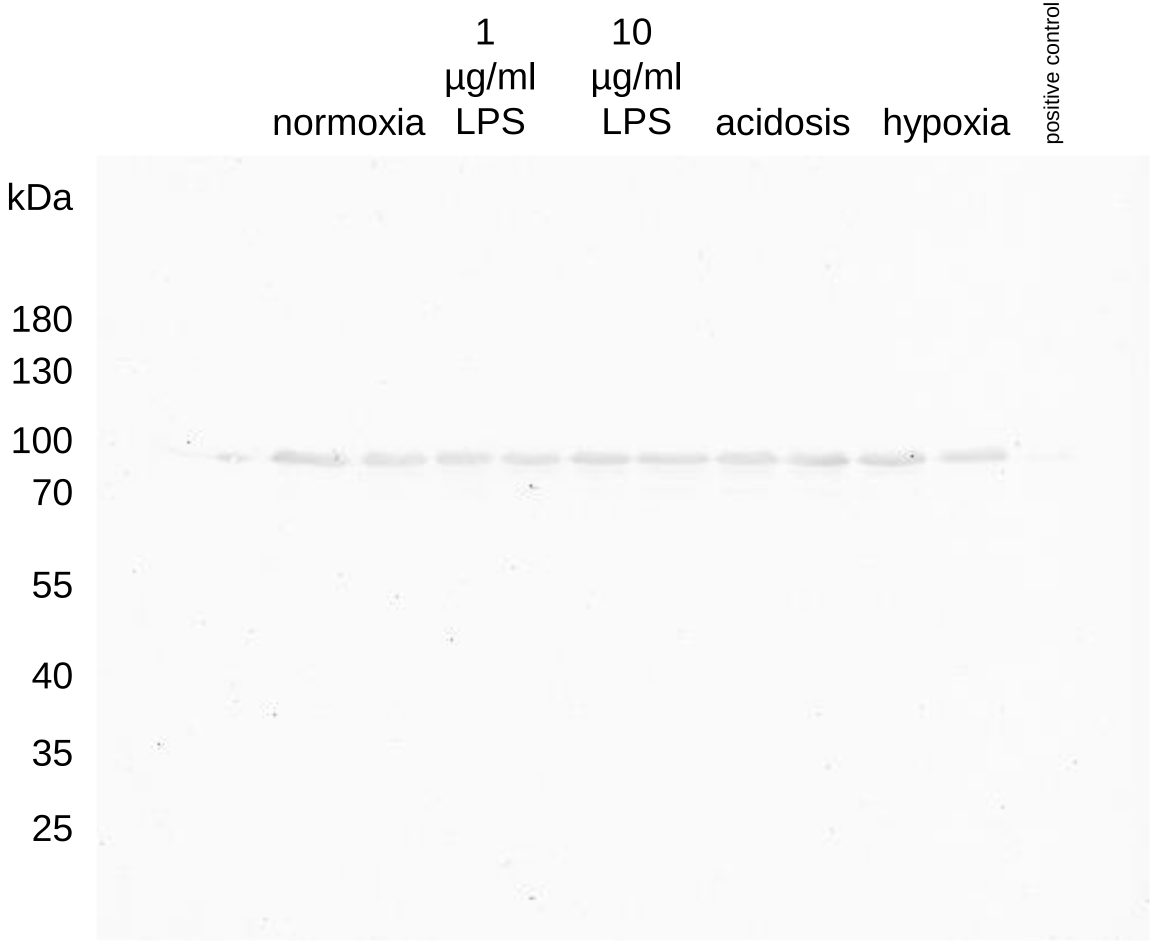

1. AB: HSP90 (1:2000) # 4874 ; Cell Signalling
2. AB: anti-Rabbit (CellSignaling) 1:2000

cell type: HepG2, passage x+26

target protein: mineralocorticoid receptor

WB date: 10.03.2022

incubation: normoxia, 1µg/ml LPS, 10µg/ml LPS, acidosis, hypoxia

positive control: myc-tagged MR (~ 110 kDa)

date of experiment: 07.03.2022

MW: ~107kDa

Marker : PageRuler (Thermo Fisher Scientific)

duration: 24h

positive control | normoxia | 1 µg/ml LPS | 10 µg/ml LPS | acidosis | hypoxia

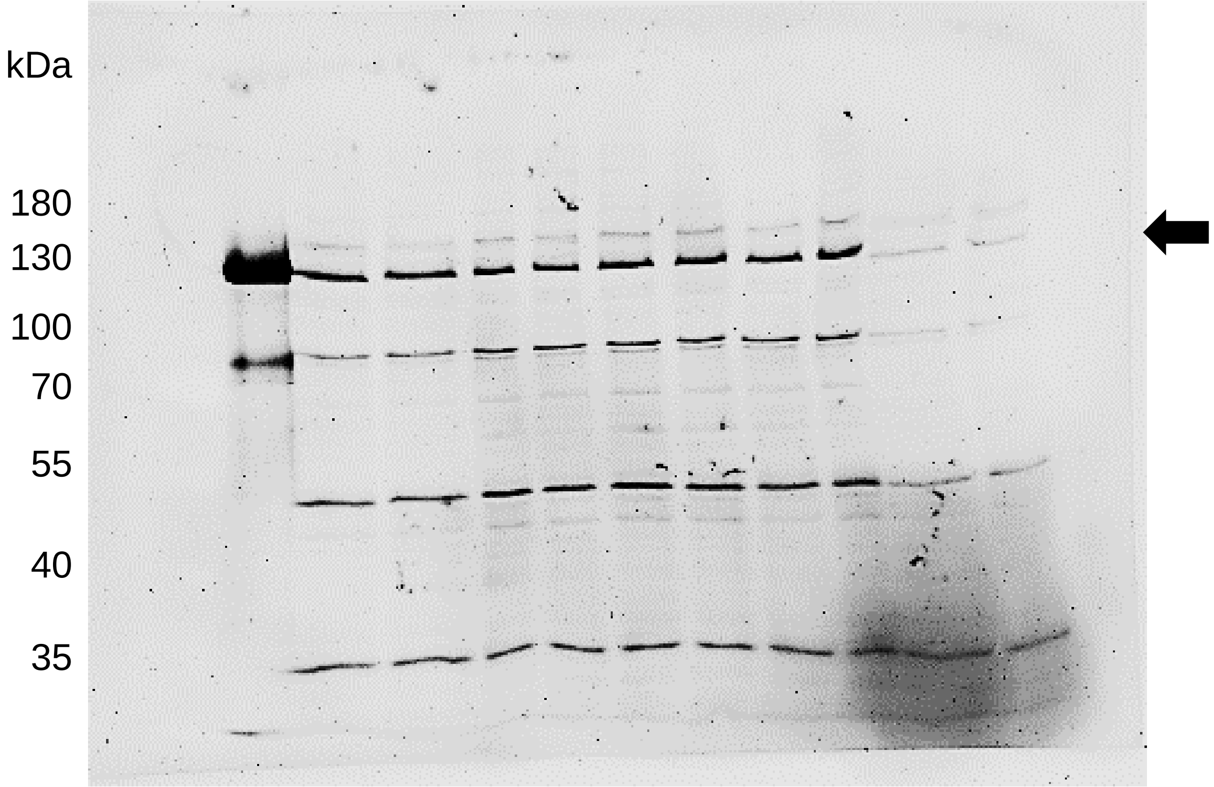

1. AB: MR (1:500) (GS 1-18)105-S
2. AB: anti-Mouse (CellSignaling) 1:1000

positive control | normoxia | 1 µg/ml LPS | 10 µg/ml LPS | acidosis | hypoxia

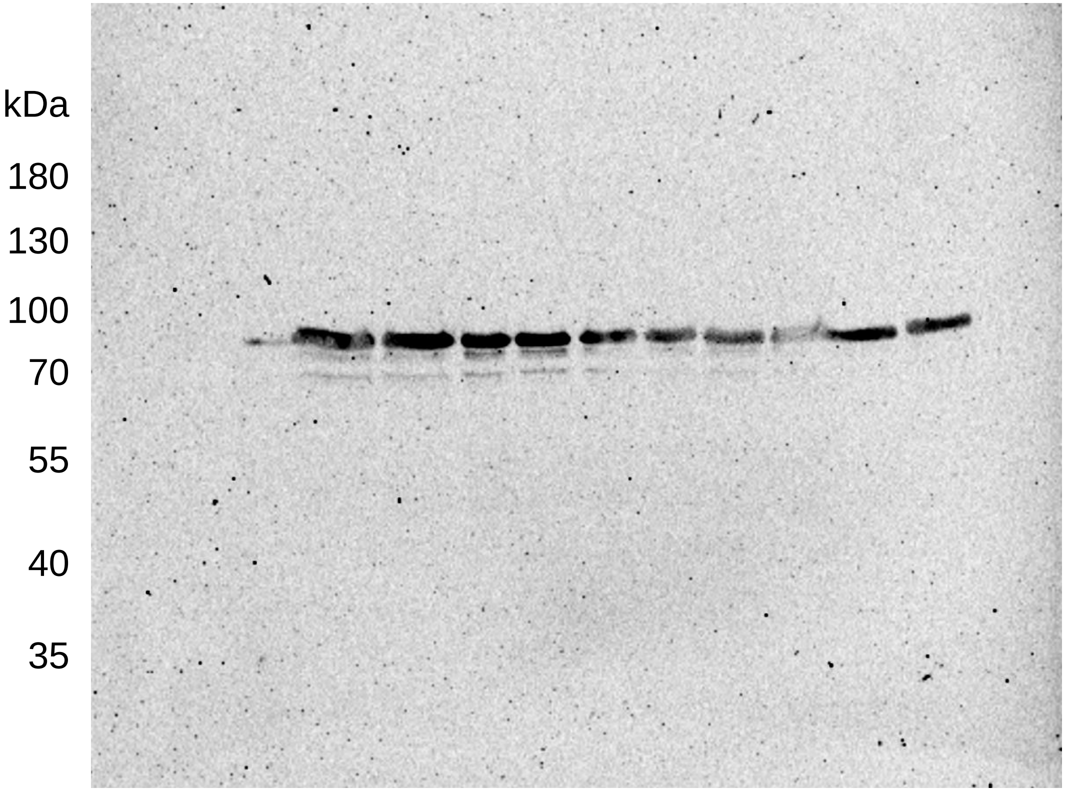

1. AB:HSP90 (1:2000) # 4874 ; Cell Signalling
2. AB anti-Rabbit (CellSignaling) 1:2000

Cell type: HepG2, passage x+24
target protein: mineralocorticoid receptor
WB date: 16.02.2023
incubation: normoxia, 1µg/ml LPS, 10µg/ml LPS, acidosis, hypoxia
positive control: myc-tagged MR (~ 110 kDa)

date of experiment: 08.04.2022
MW: ~107kDa
Marker : PageRuler (Thermo Fisher Scientific)
duration: 24h

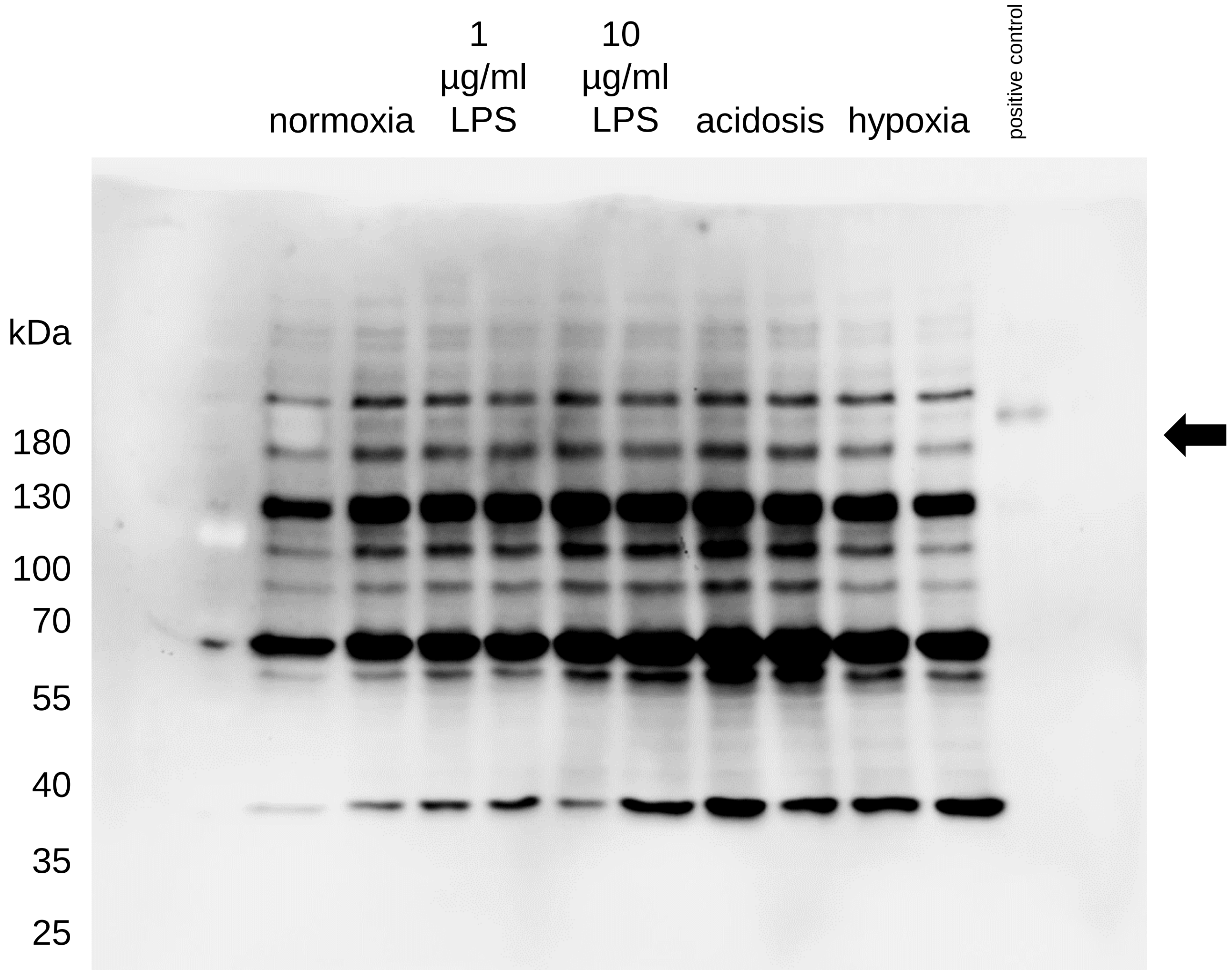

1. AB: MR (1:500) (GS 1-18)105-S
2. AB: anti-Mouse (CellSignaling) 1:1000

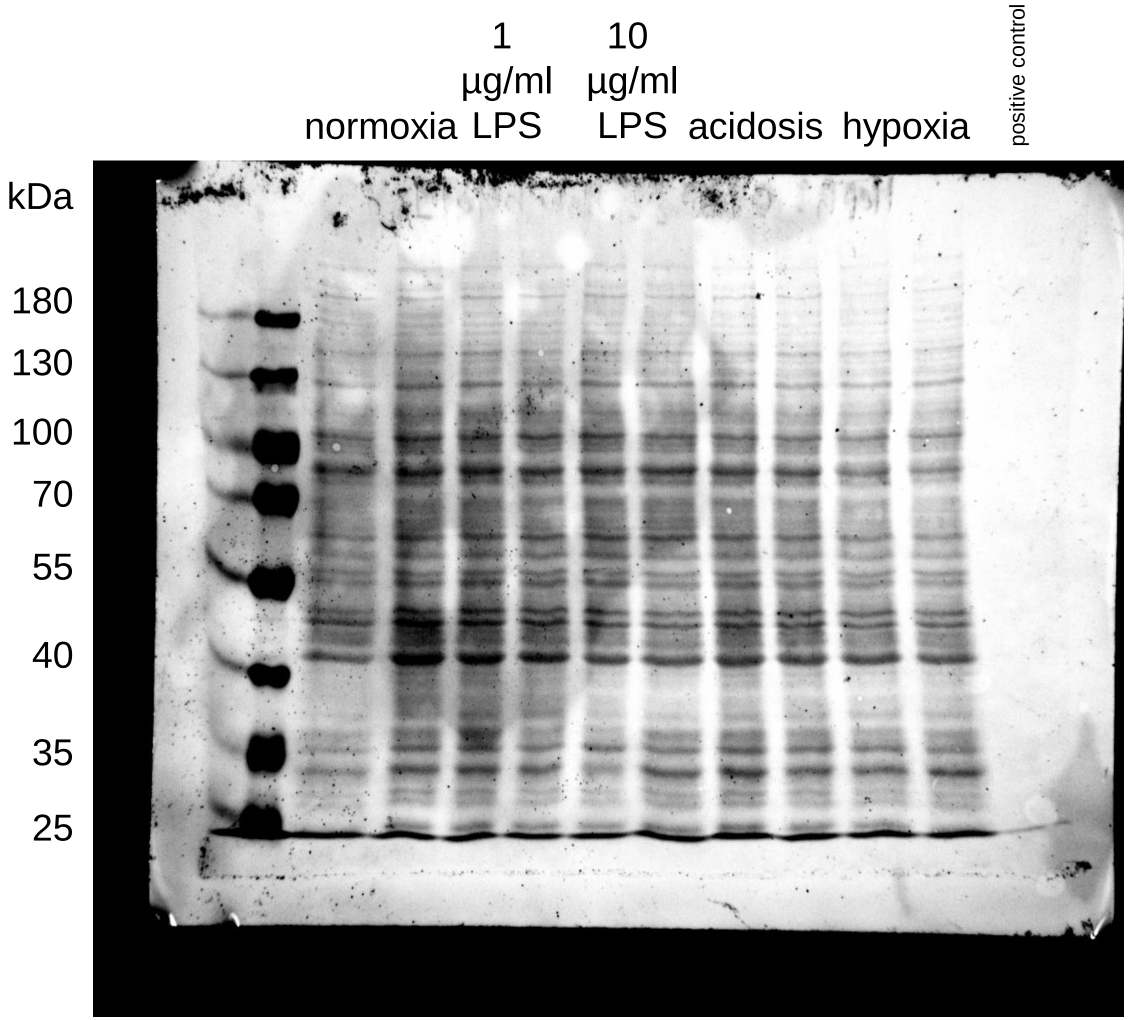

Ponceau

cell type: HepG2, passage x+12
target protein: mineralocorticoid receptor
WB date: 16.02.2023
incubation: normoxia, 1µg/ml LPS, 10µg/ml LPS, acidosis, hypoxia
positive control: myc-tagged MR (~ 110 kDa)

date of experiment: 08.04.2022
MW: ~107kDa
Marker : PageRuler (Thermo Fisher Scientific)
duration: 24h

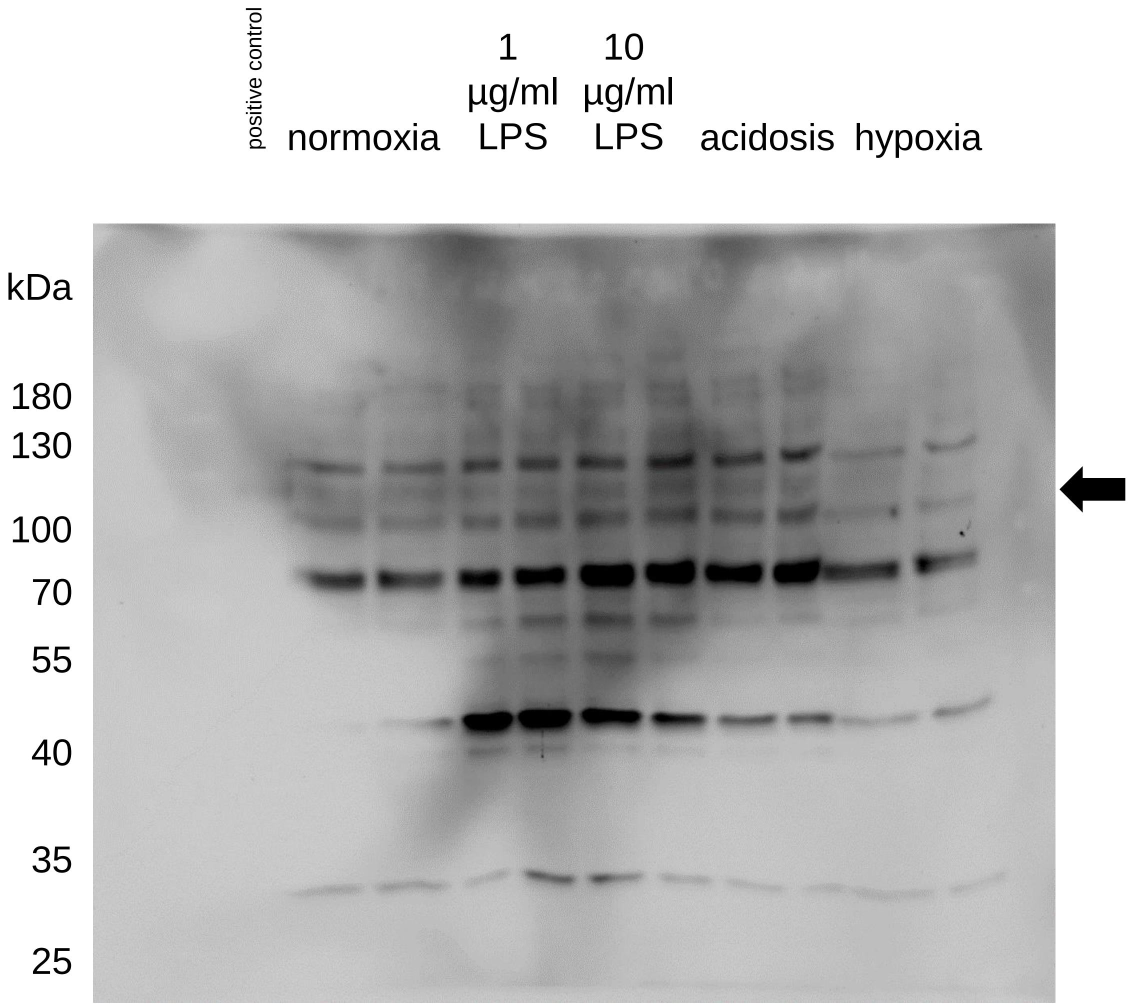

1. AB: MR (1:500) (GS 1-18)105-S
2. AB: anti-Mouse (CellSignaling) 1:1000

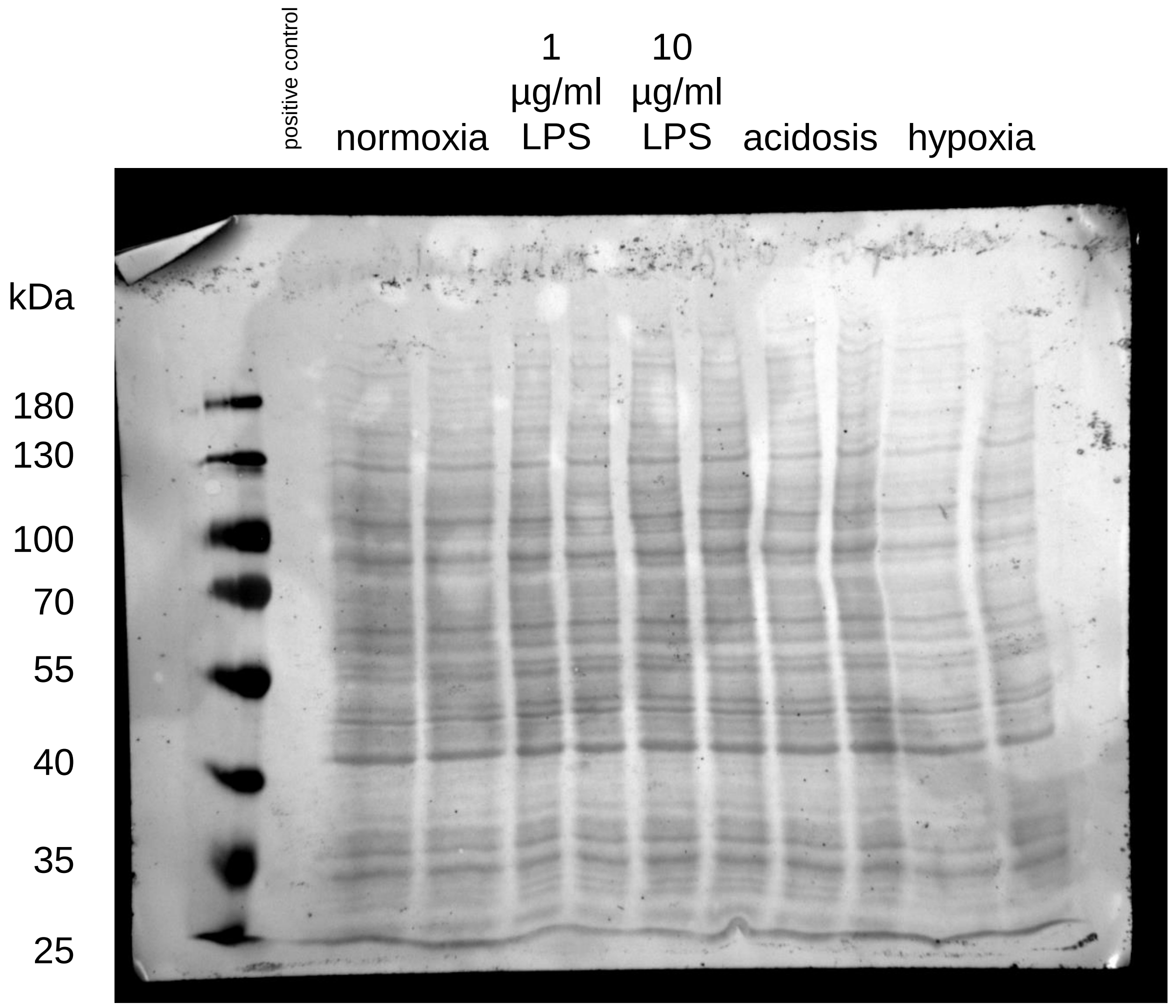

Ponceau

Western blots for figure 5

Cell type: HepG2, passage x+7 **date of experiment:** 16.12.2022
target protein: PPARα **MW:** ~52 kDa
WB date: 20.12.2022 **Marker :** PageRuler (Thermo Fisher Scientific)
Incubation: normoxia, normoxia & epl, hypoxia, hypoxia & epl **duration:** 24h

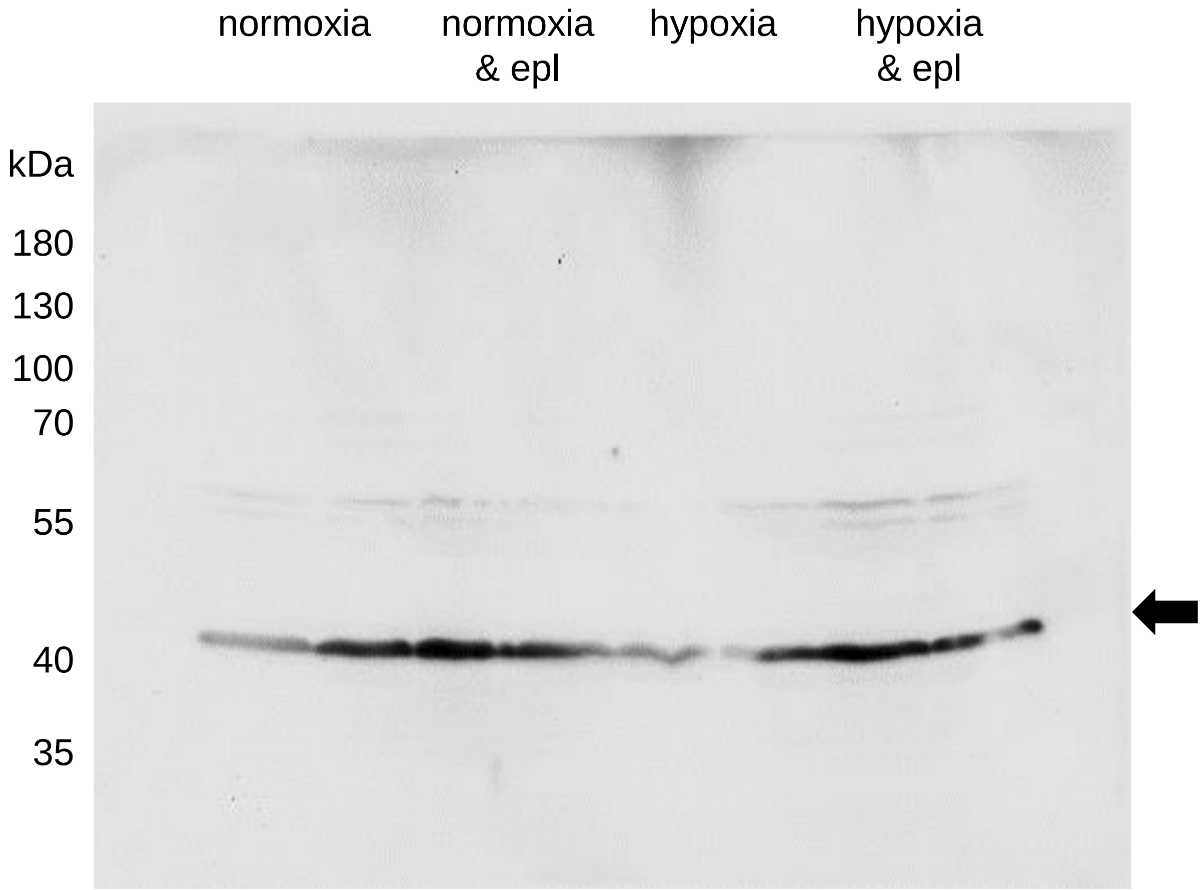

1. AB: PPARa (1:500) ABCAM (ab126285)
2. AB: anti-Rabbit (CellSignaling) 1:2000

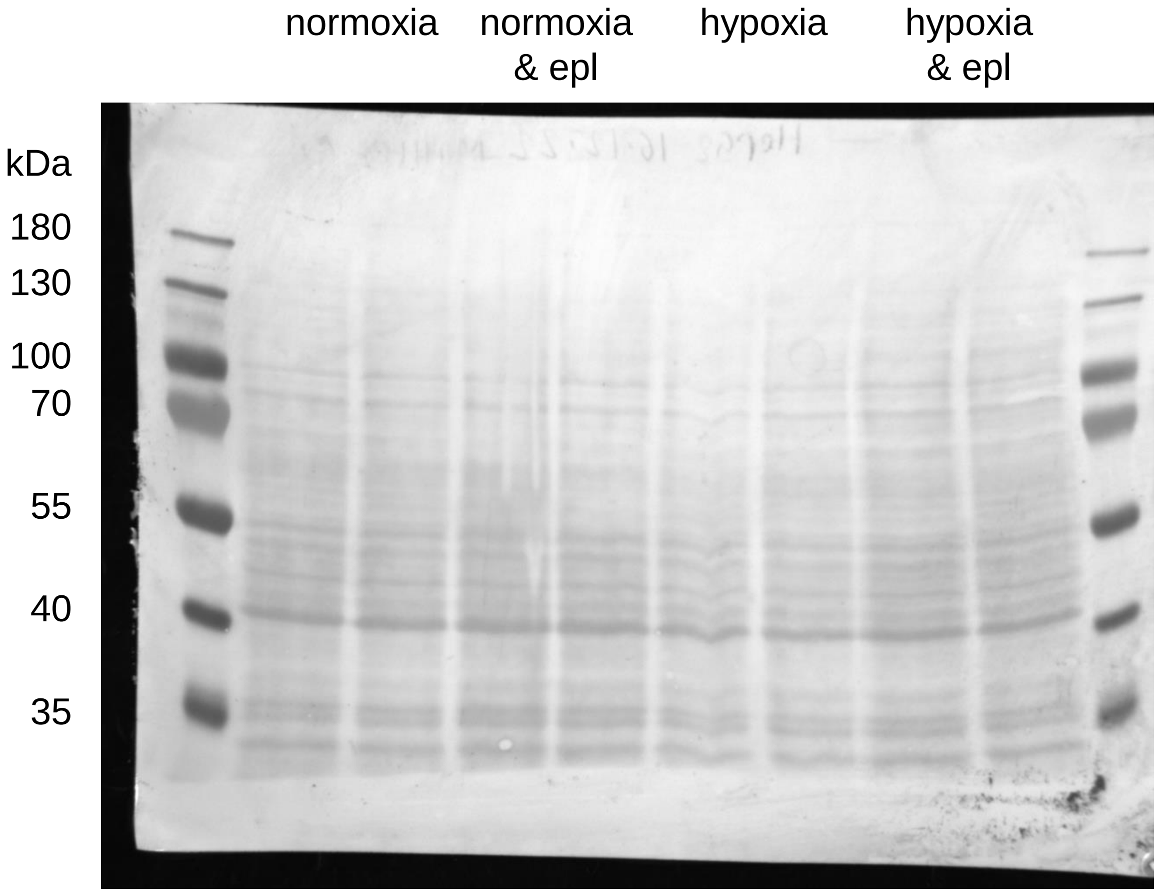

Ponceau

cell type: HepG2, passage x+8

date of experiment: 16.12.2022

target protein: PPARα

MW: ~52 kDa

WB date: 20.12.2022

Marker : PageRuler (Thermo Fisher Scientific)

incubation: normoxia, normoxia & epl, hypoxia, hypoxia & epl

duration: 24h

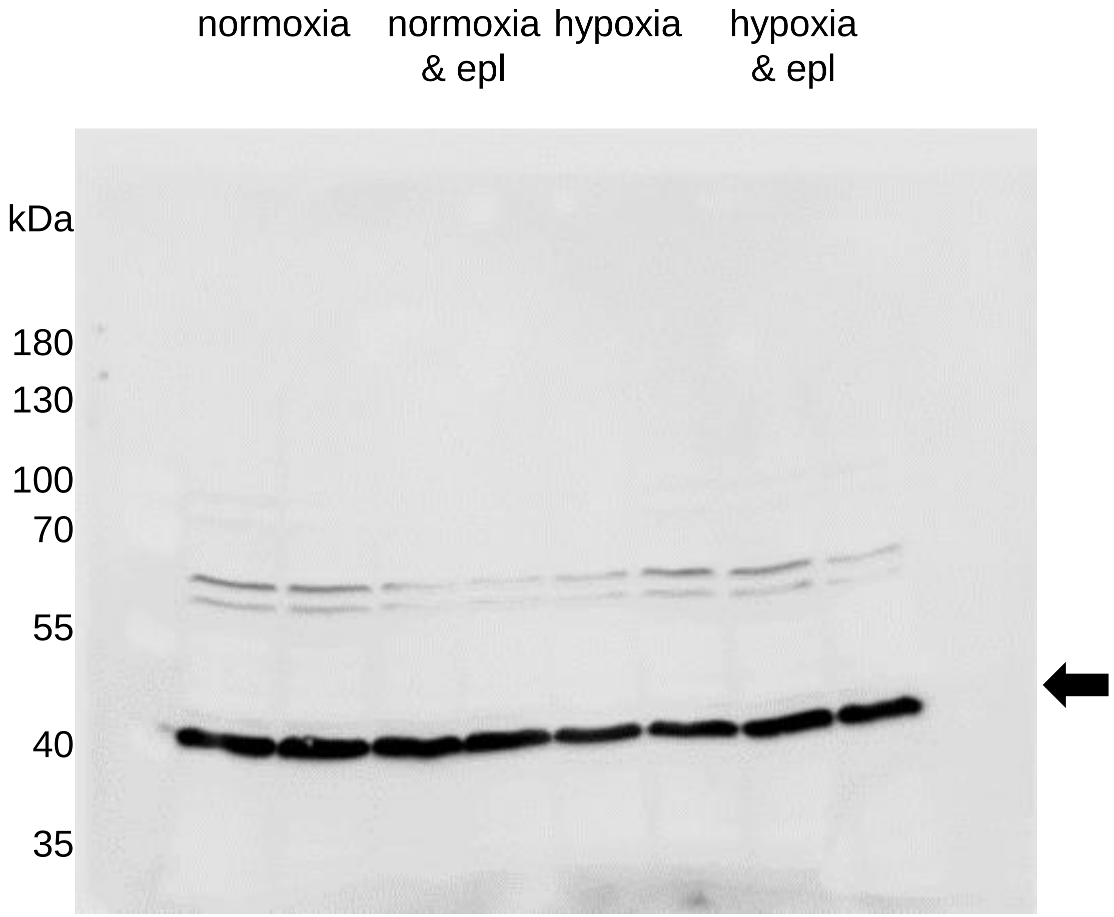

1. AB: PPARa (1:500) ABCAM (ab126285)
2. AB: anti-Rabbit (CellSignaling) 1:2000

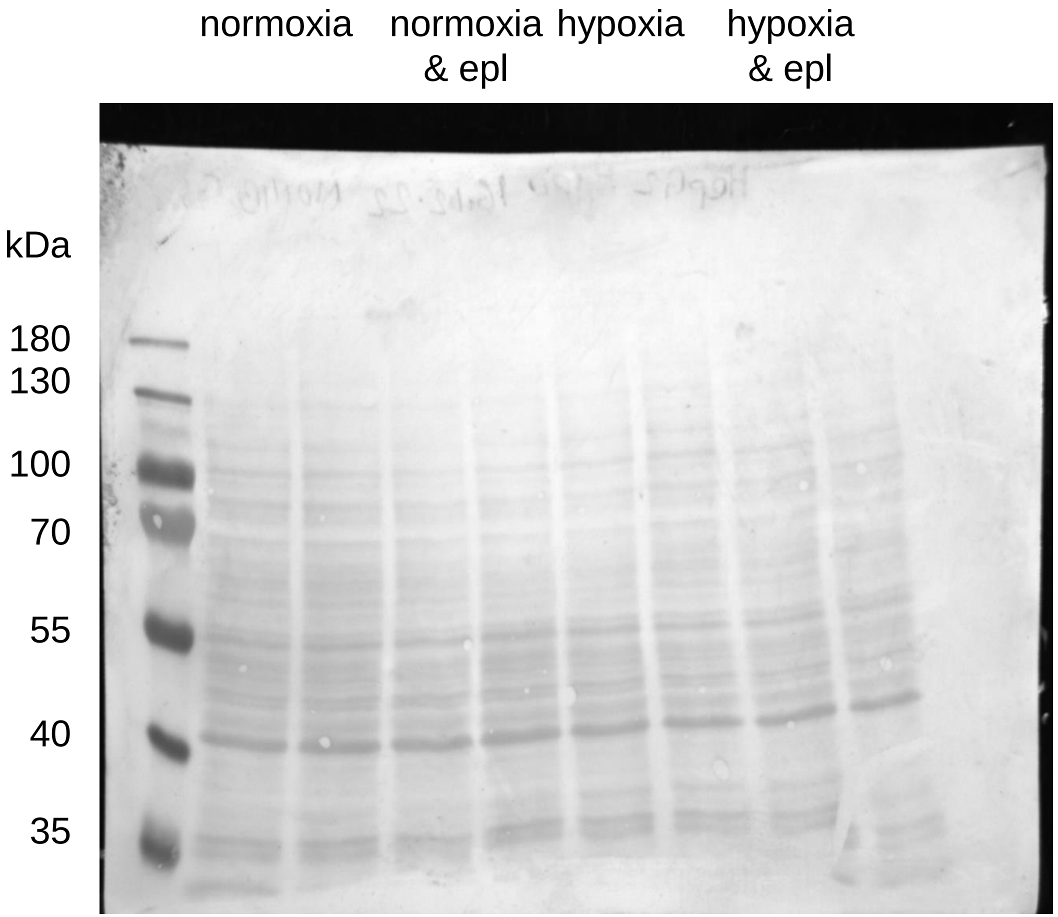

Ponceau

cell type: HepG2, passage x+9 **date of experiment:** 16.12.2022
target protein: PPARα **MW:** ~52 kDa
WB date: 27.02.2023 **Marker :** PageRuler (Thermo Fisher Scientific)
incubation: normoxia, normoxia & epl, hypoxia, hypoxia & epl **duration:** 24h

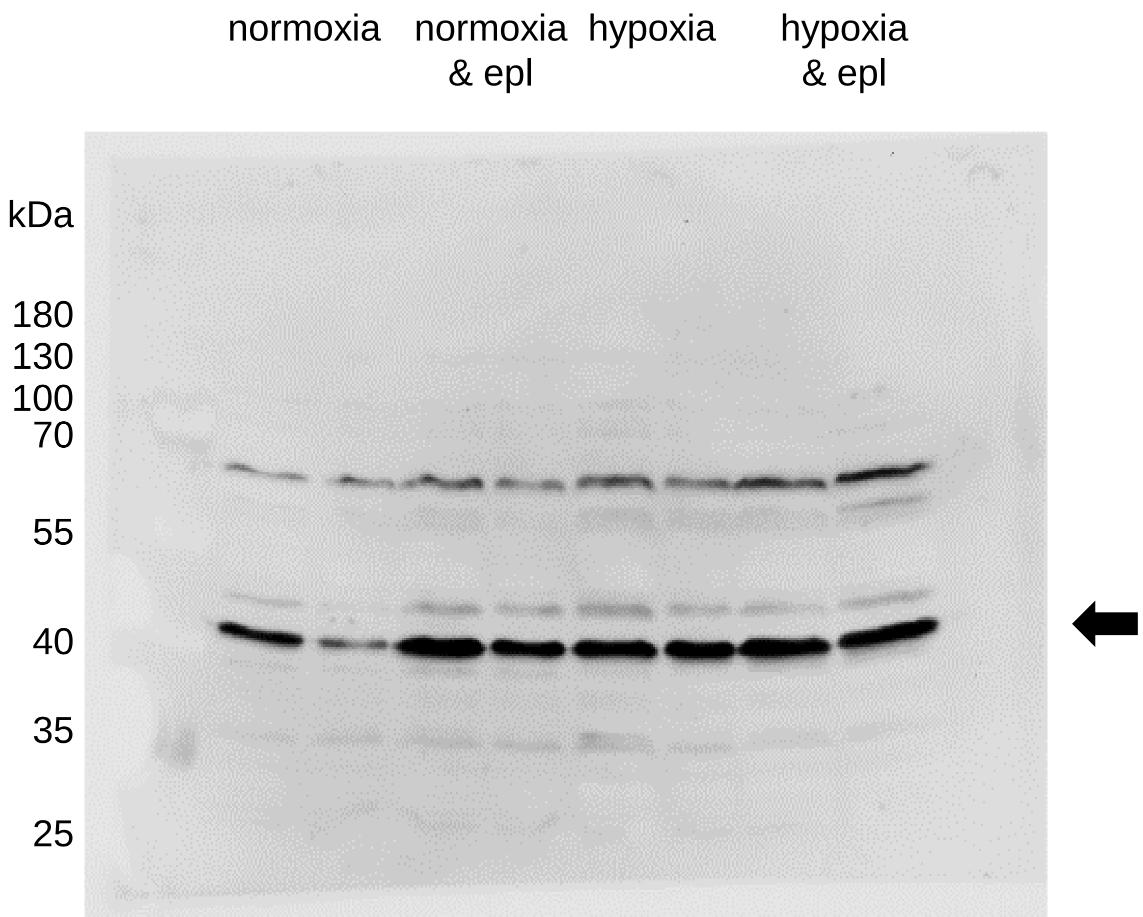

1. AB: PPARa (1:500) ABCAM (ab126285)
2. AB: anti-Rabbit (CellSignaling) 1:2000

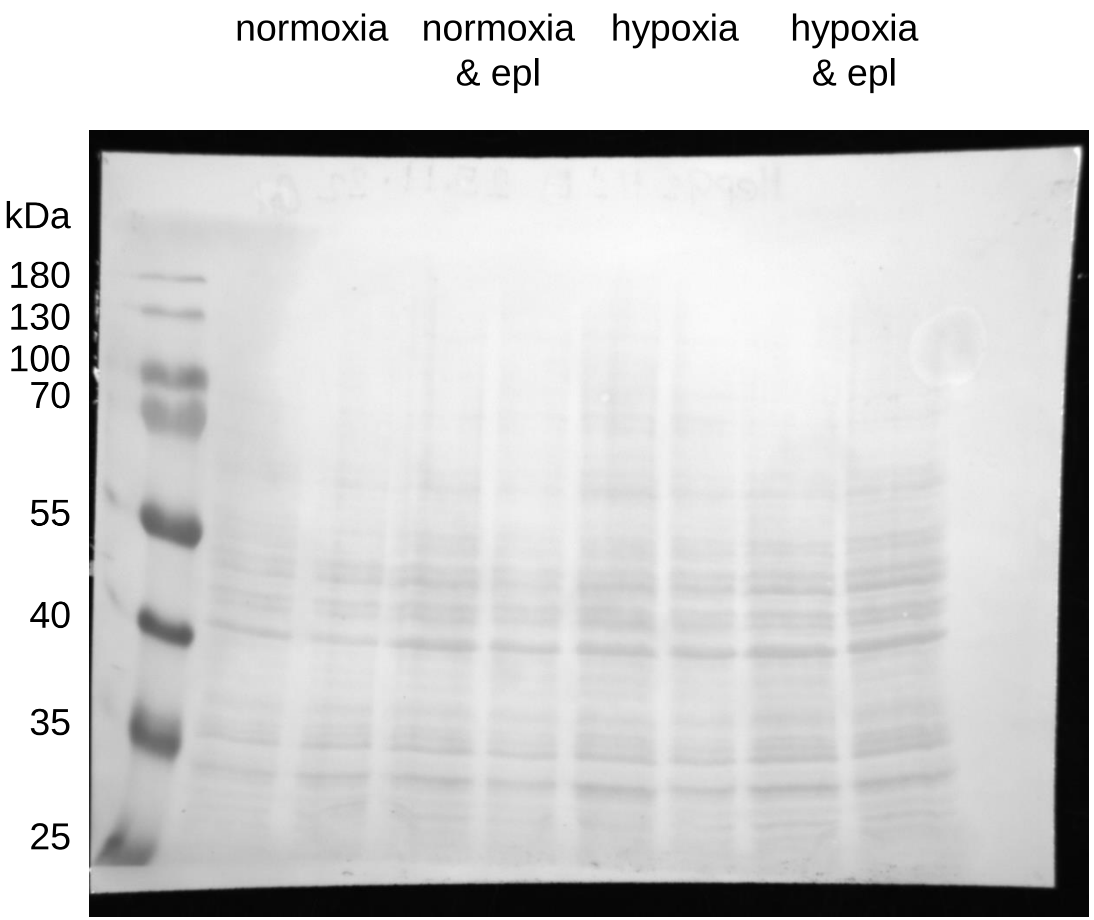

Ponceau

cell type: HepG2, passage x+11
target protein: PPARα
WB date: 25.01.2023
incubation: normoxia, normoxia & epl, hypoxia, hypoxia & epl

date of experiment: 22.12.2022
MW: ~52 kDa
Marker : PageRuler (Thermo Fisher Scientific)
duration: 24h

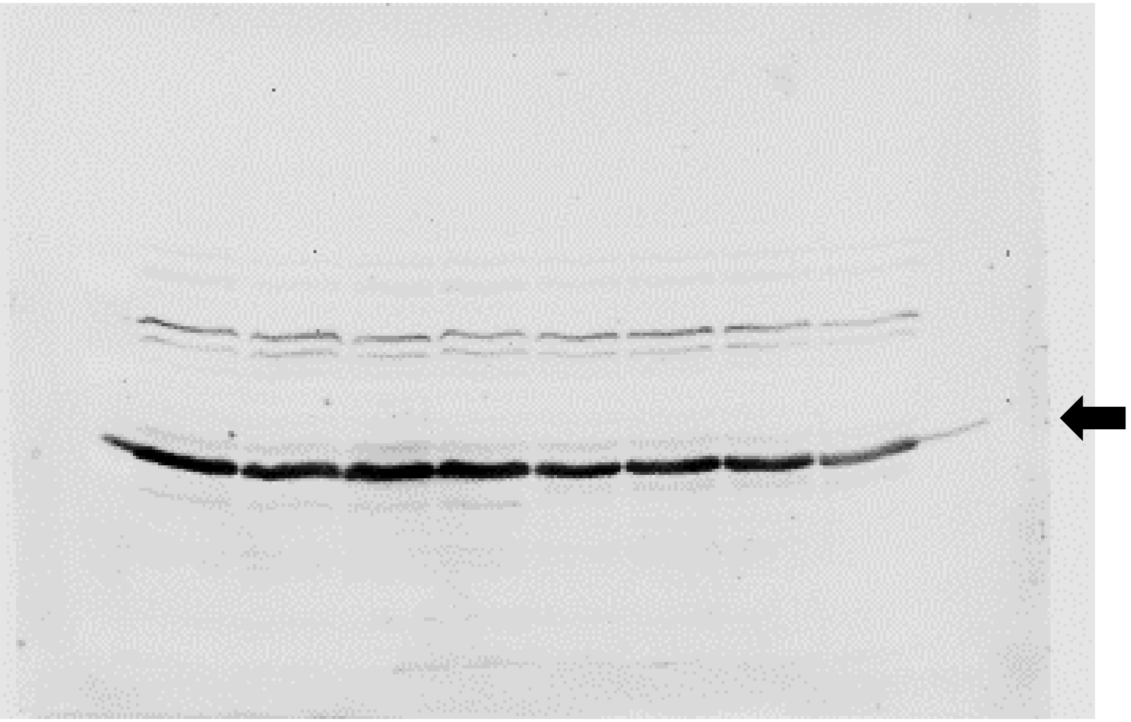

1. AB: PPARa (1:500) ABCAM (ab126285)
2. AK anti-Rabbit (CellSignaling) 1:2000

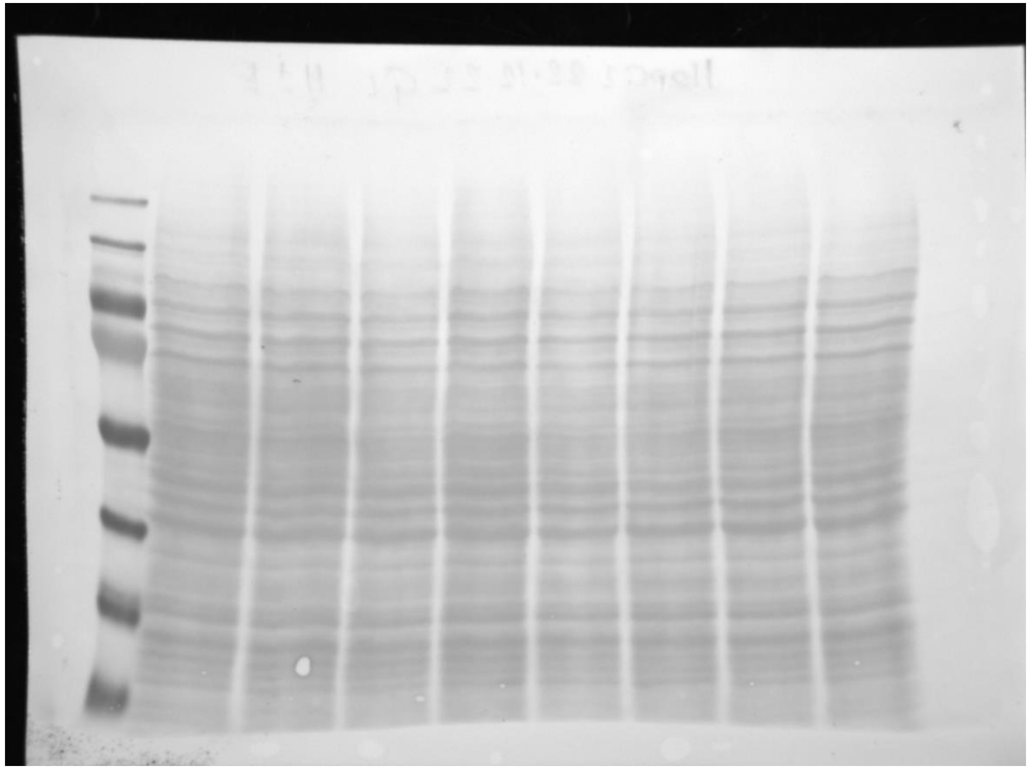

Ponceau

cell type: HepG2, passage x+12
date of experiment: 23.12.2022
target protein: PPARα
MW: ~52 kDa
WB date: 25.01.2023
Marker : PageRuler (Thermo Fisher Scientific)
incubation: normoxia, normoxia & epl, hypoxia, hypoxia & epl
duration: 24h

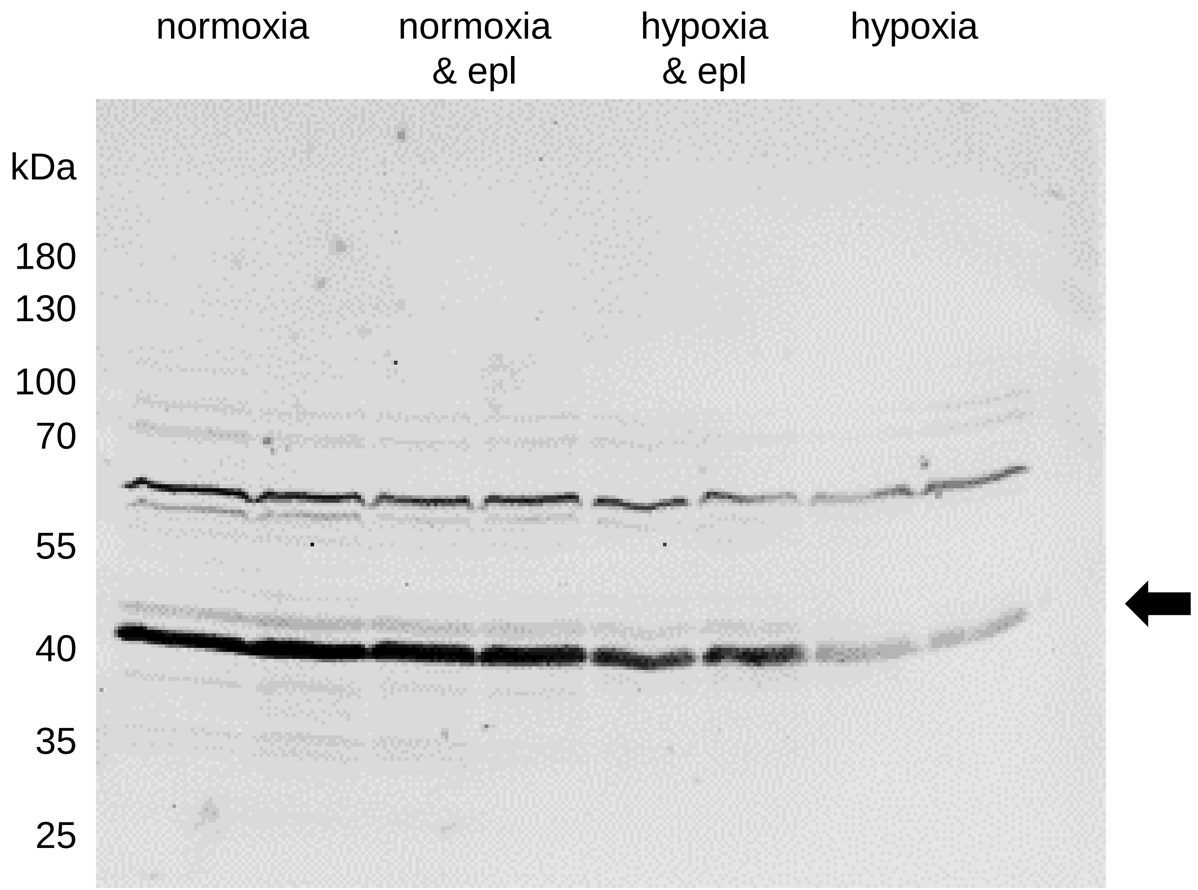

1. AB: PPARa (1:500) ABCAM (ab126285)
2. AB: anti-Rabbit (CellSignaling) 1:2000

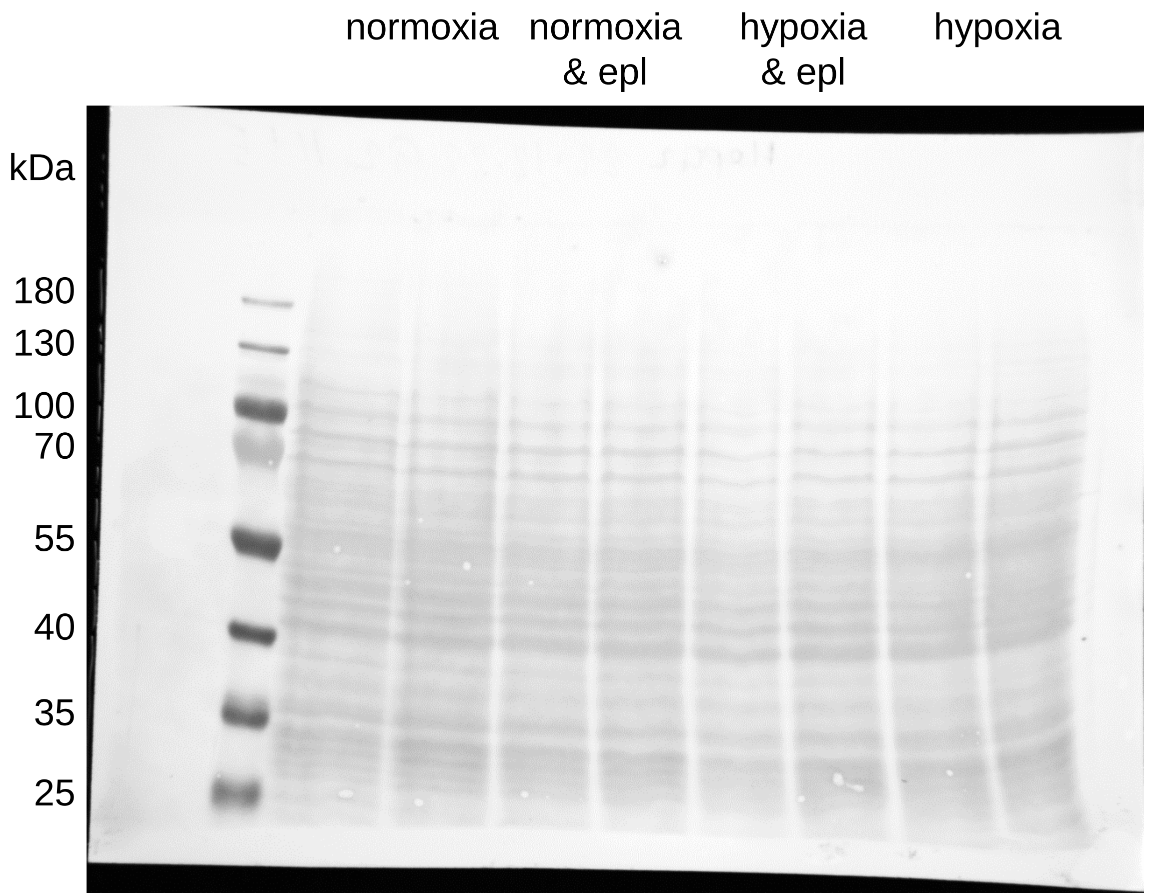

Ponceau

cell type: HepG2, passage x+6 **date of experiment:** 25.01.2023
target protein: PPARα **MW:** ~52 kDa
WB date: 08.03.2023 **Marker :** PageRuler (Thermo Fisher Scientific)
incubation: normoxia, normoxia & epl, hypoxia, hypoxia & epl **duration:** 24h

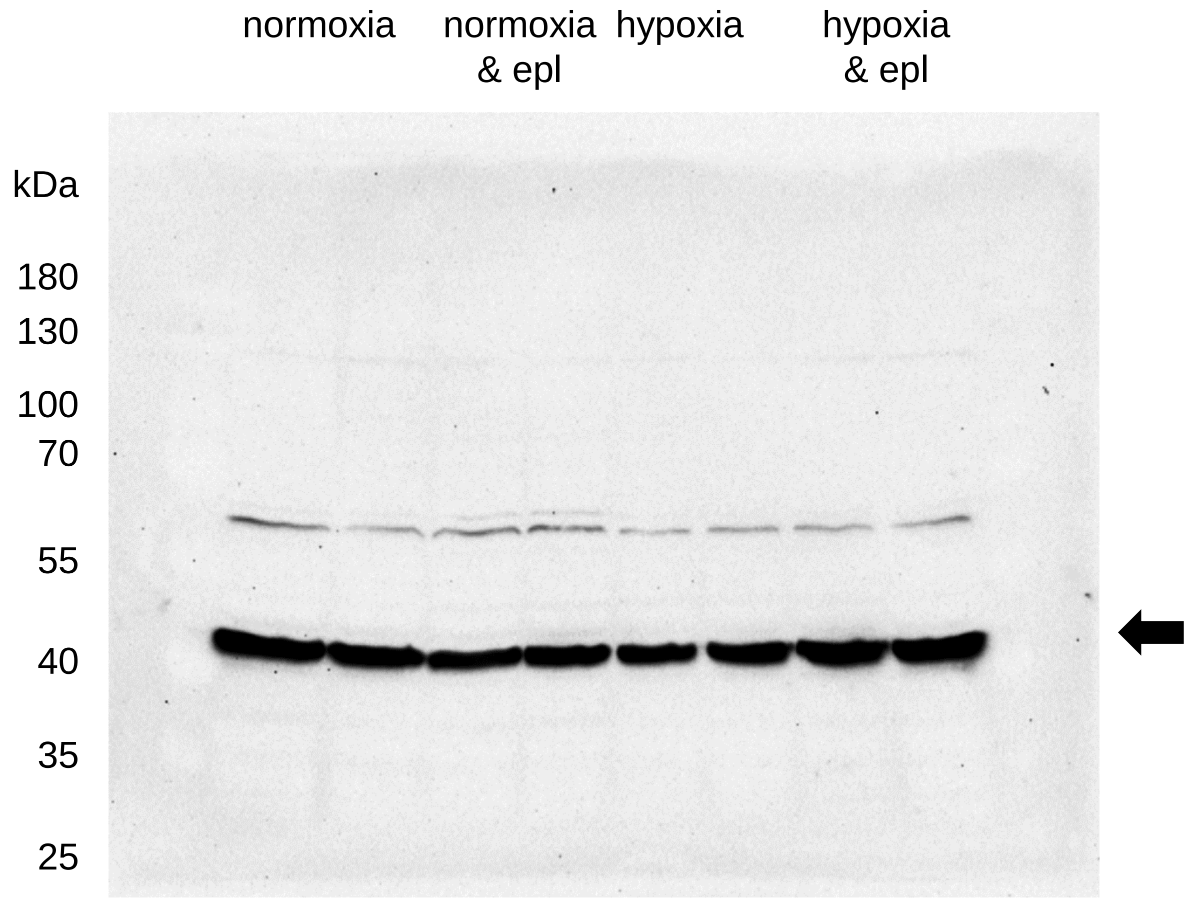

1. AB: PPARa (1:500) ABCAM (ab126285)
2. AB: anti-Rabbit (CellSignaling) 1:2000

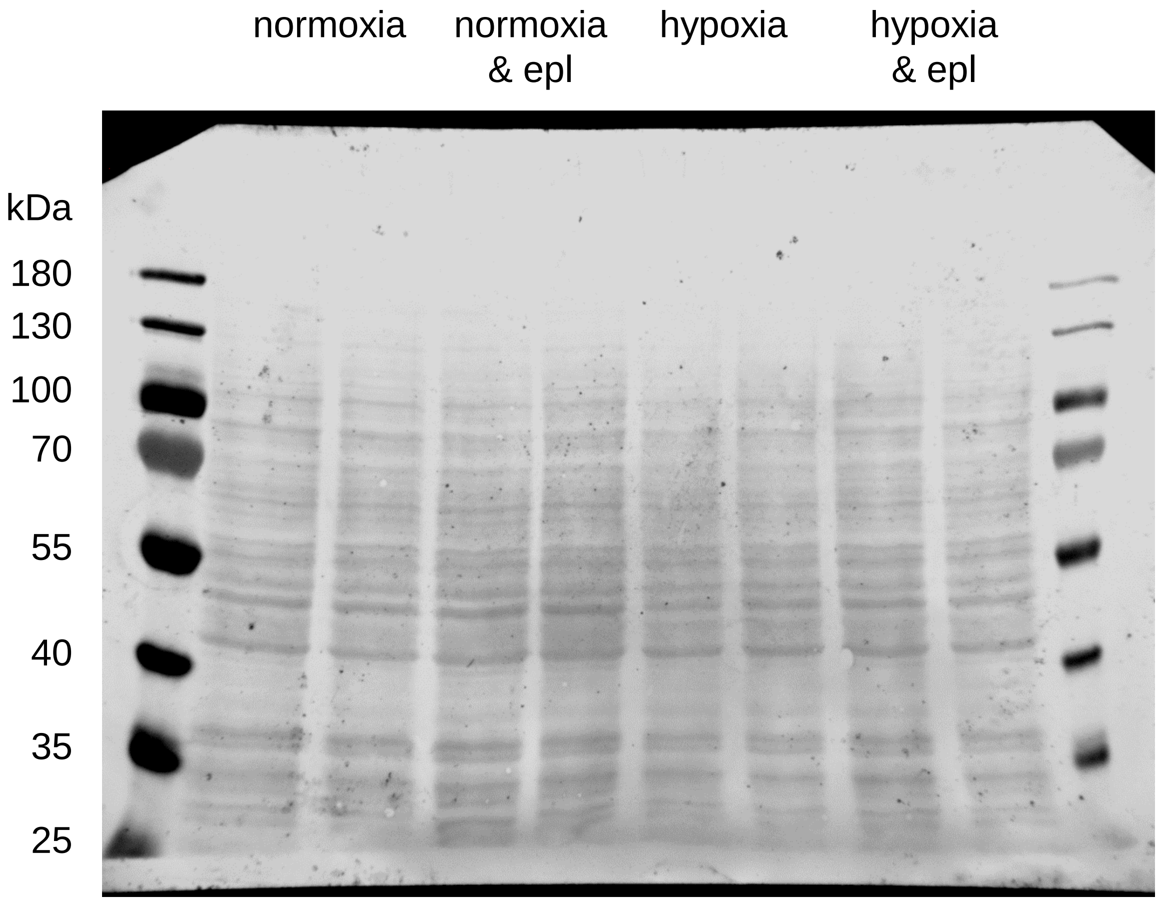

Ponceau

cell type: HepG2, passage x+8
date of experiment: 25.11.2022
target protein: PDK4
MW: ~46 kDa
WB date: 07.12.2022
Marker : PageRuler (Thermo Fisher Scientific)
incubation: normoxia, normoxia & epl, hypoxia, hypoxia & epl
duration: 24h

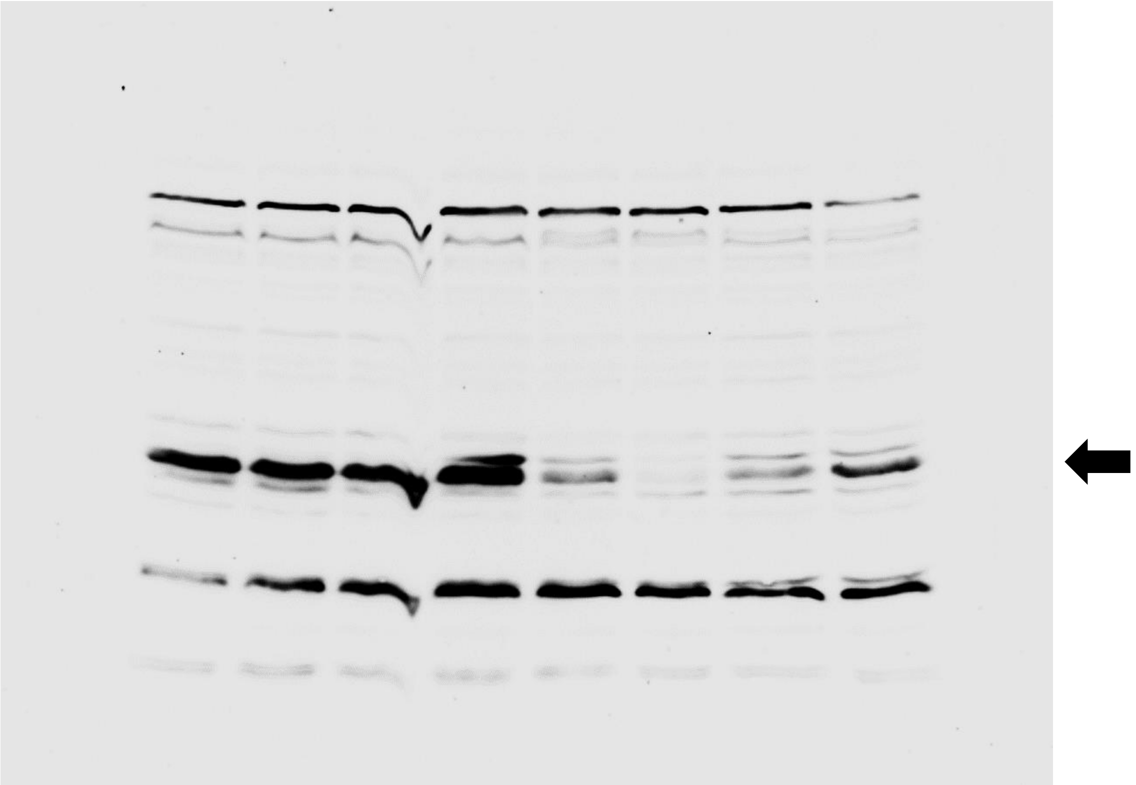

1. AB: PDK4 (1:1000) Boston
2. AB: anti-Rabbit (CellSignaling) 1:2000

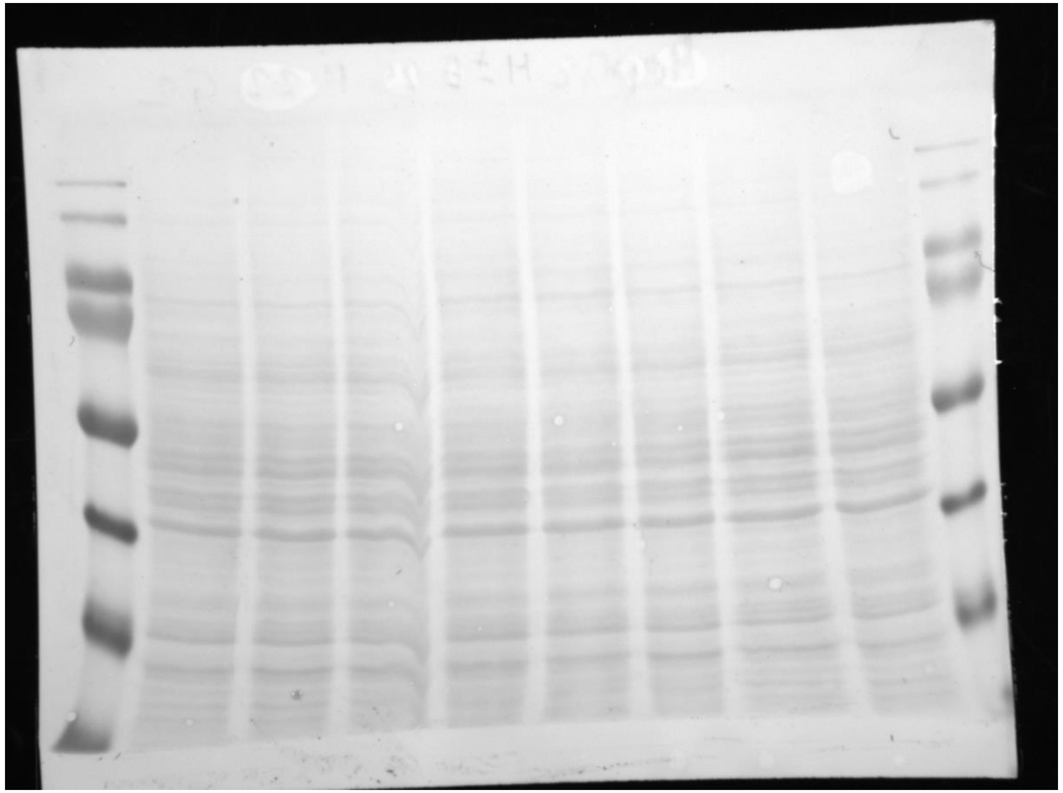

Ponceau

cell type: HepG2, passage x+7
target protein: PDK4
WB date: 20.12.2022
incubation: normoxia, normoxia & epl, hypoxia, hypoxia & epl
positive control: pCMV6-PDK4 (~ 50 kDa)

date of experiment: 16.12.2022
MW: ~46 kDa
Marker : PageRuler (Thermo Fisher Scientific)
duration: 24h

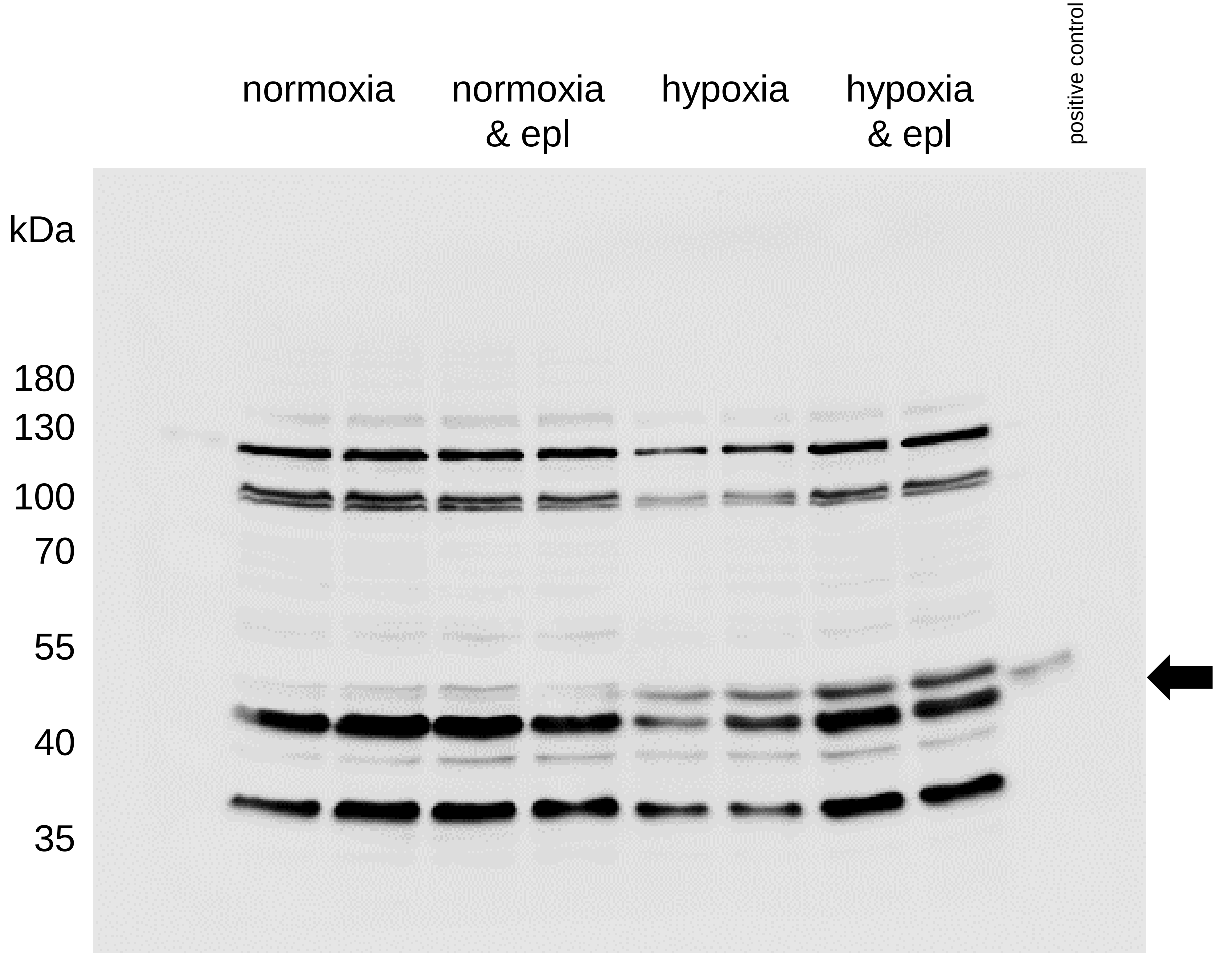

1. AB: PDK4 (1:1000) Boston
2. AB: anti-Rabbit (CellSignaling) 1:2000

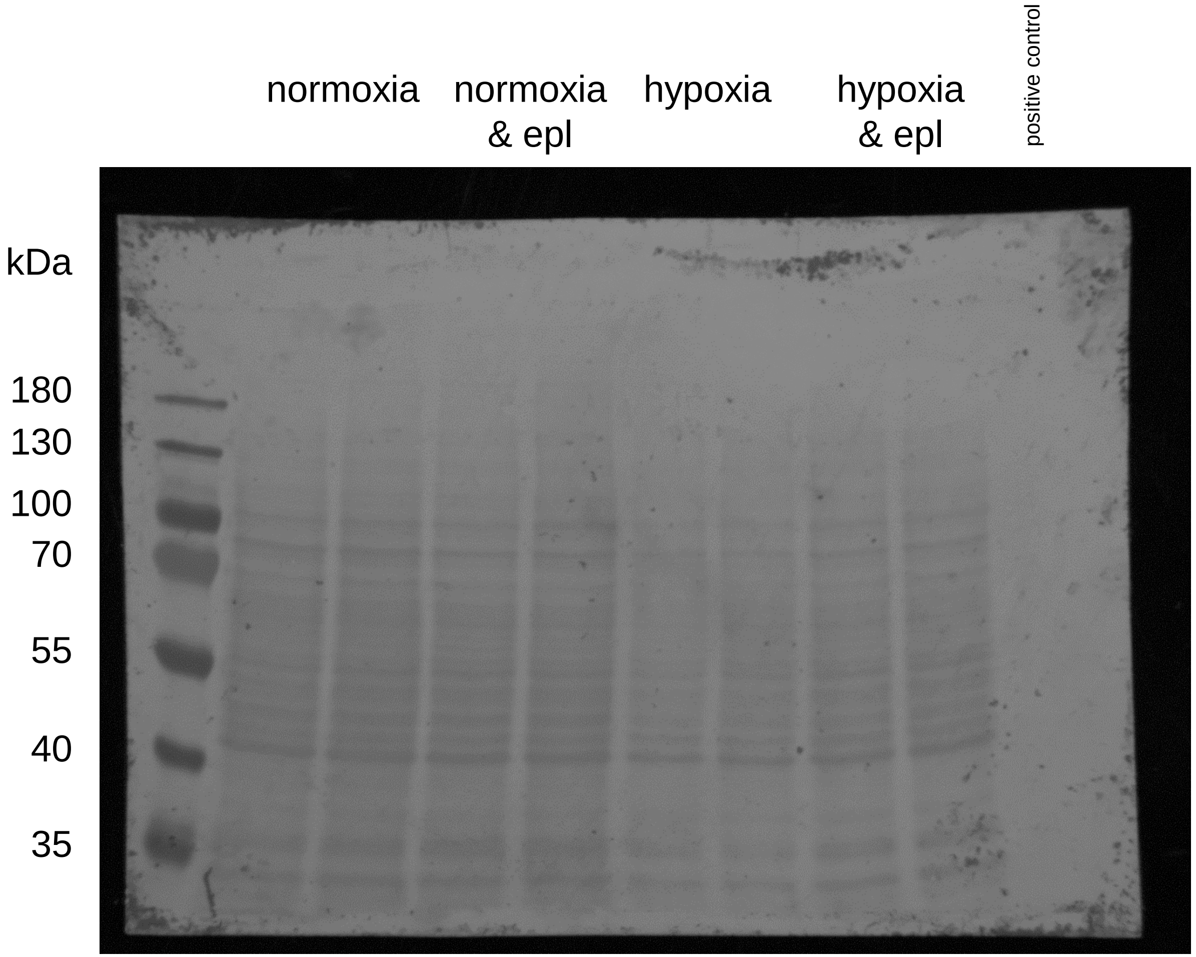

Ponceau

cell type: HepG2, passage x+8
target protein: PDK4
WB date: 18.01.2023
incubation: normoxia, normoxia & epl, hypoxia, hypoxia & epl
positive control: pCMV6-PDK4 (~ 50 kDa)

date of experiment: 16.12.2022
MW: ~46 kDa
Marker : PageRuler (Thermo Fisher Scientific)
duration: 24h

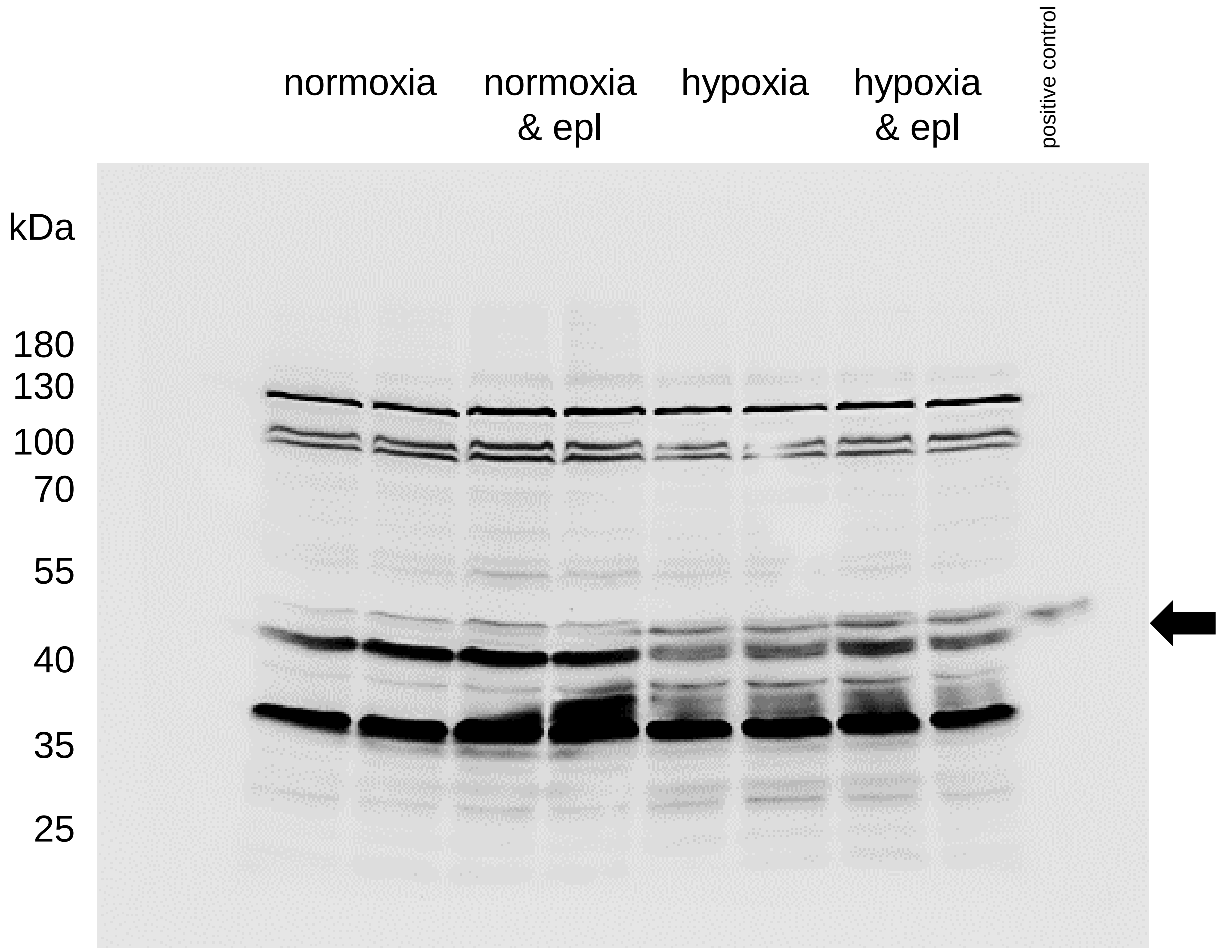

1. AB: PDK4 (1:1000) Boston
2. AB: anti-Rabbit (CellSignaling) 1:2000

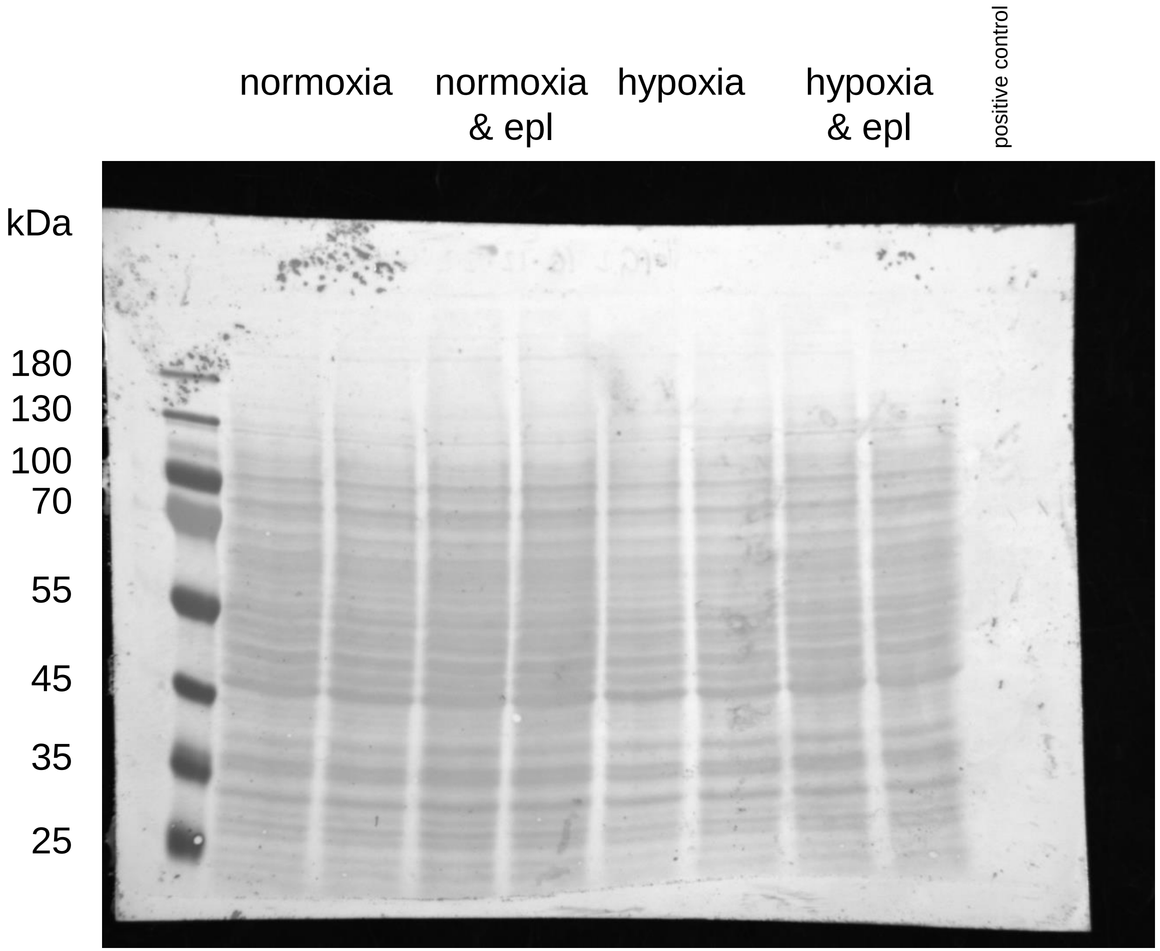

Ponceau

cell type: HepG2 cells, passage x+9
date of experiment: 16.12.2022
target protein: PDK4
MW: ~46 kDa
WB date: 18.01.2023
Marker : PageRuler (Thermo Fisher Scientific)
incubation: normoxia, normoxia & epl, hypoxia, hypoxia & epl
duration: 24h
positive control: pCMV6-PDK4 (~ 50 kDa)

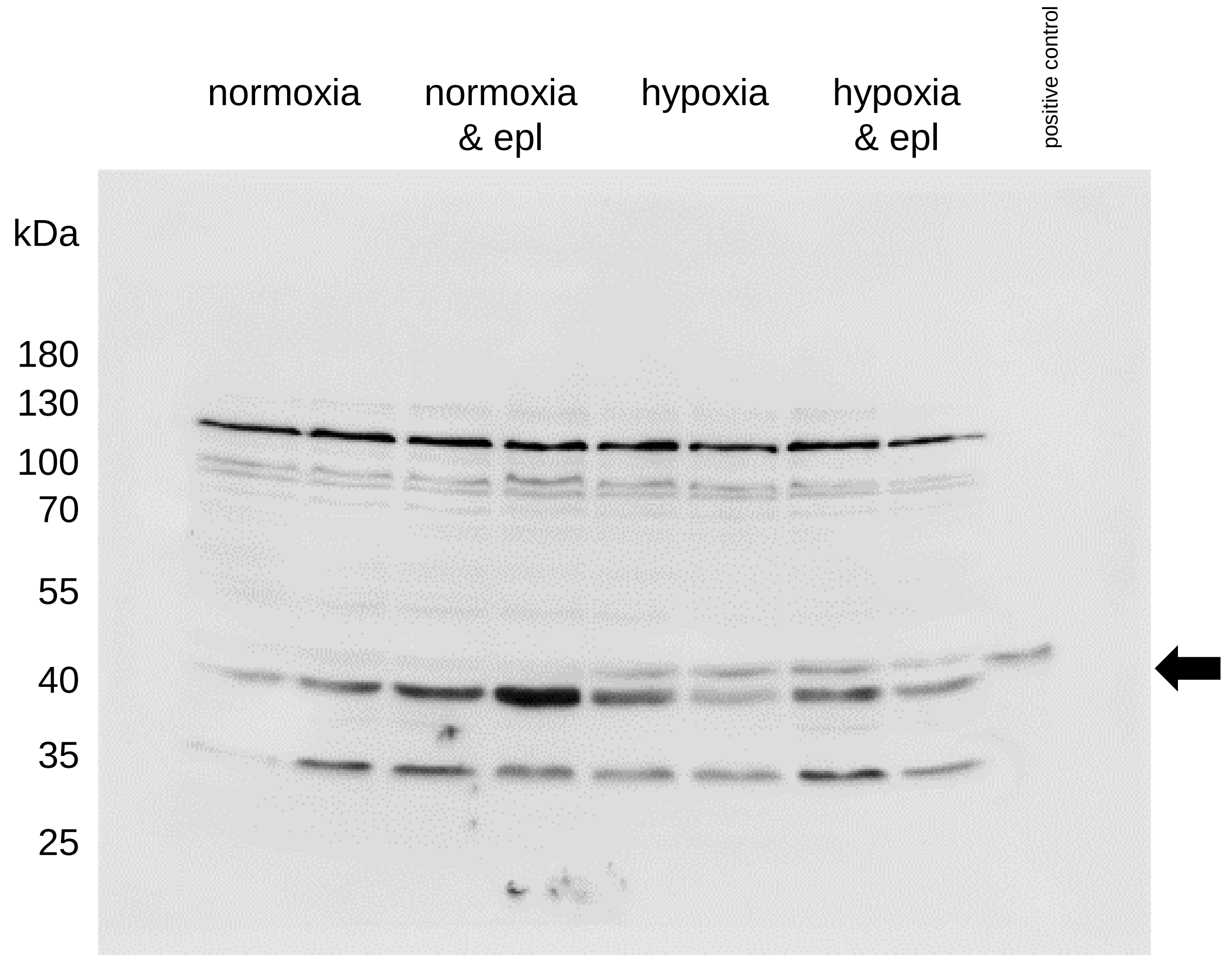

1. AB: PDK4 (1:1000) Boston
2. AB: anti-Rabbit (CellSignaling) 1:2000

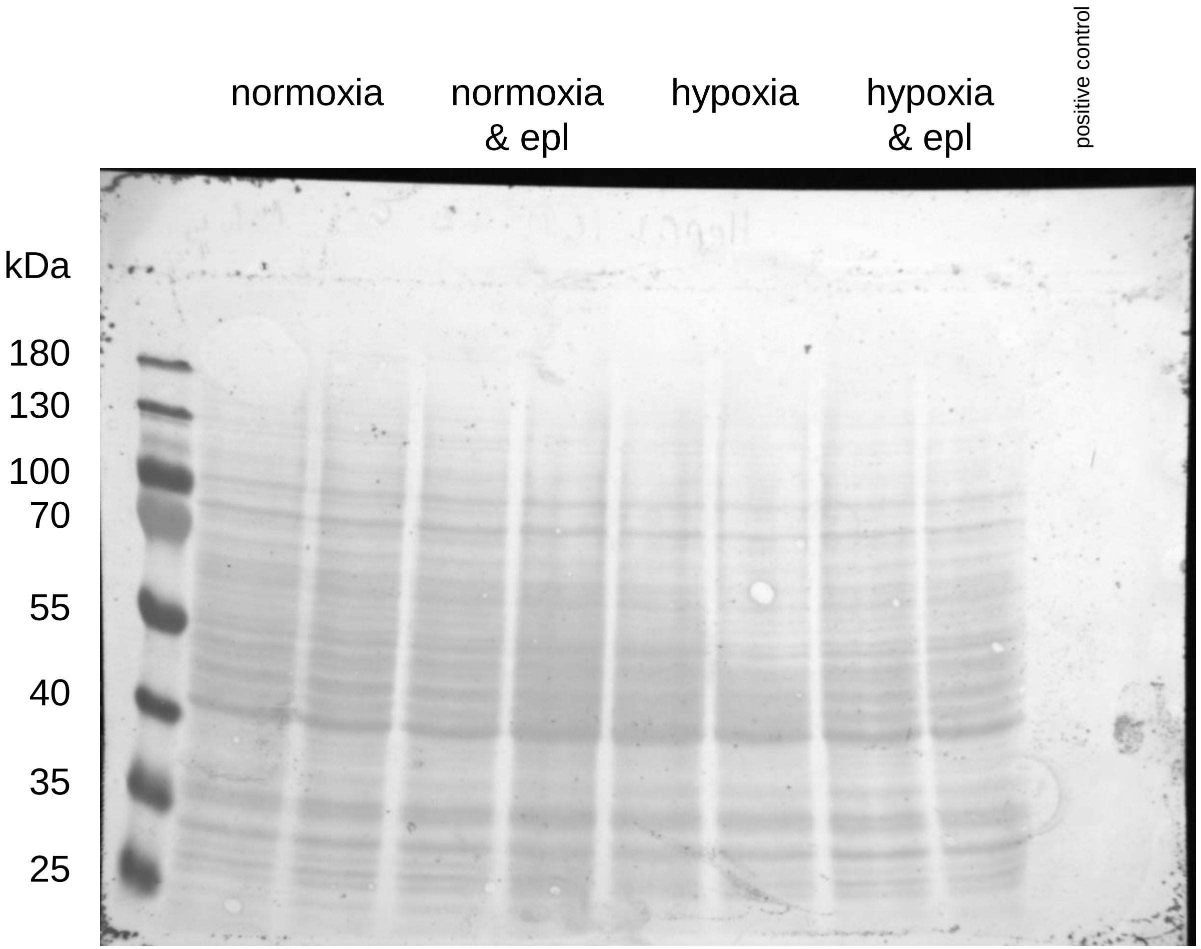

Ponceau

cell type: HepG2, passage x+11
target protein: PDK4
WB date: 18.01.2023
Incubation: normoxia, normoxia & epl, hypoxia, hypoxia & epl
positive control: pCMV6-PDK4 (~ 50 kDa)

date of experiment: 22.12.2022
MW: ~46kDa
Marker : PageRuler (Thermo Fisher Scientific)
duration: 24h

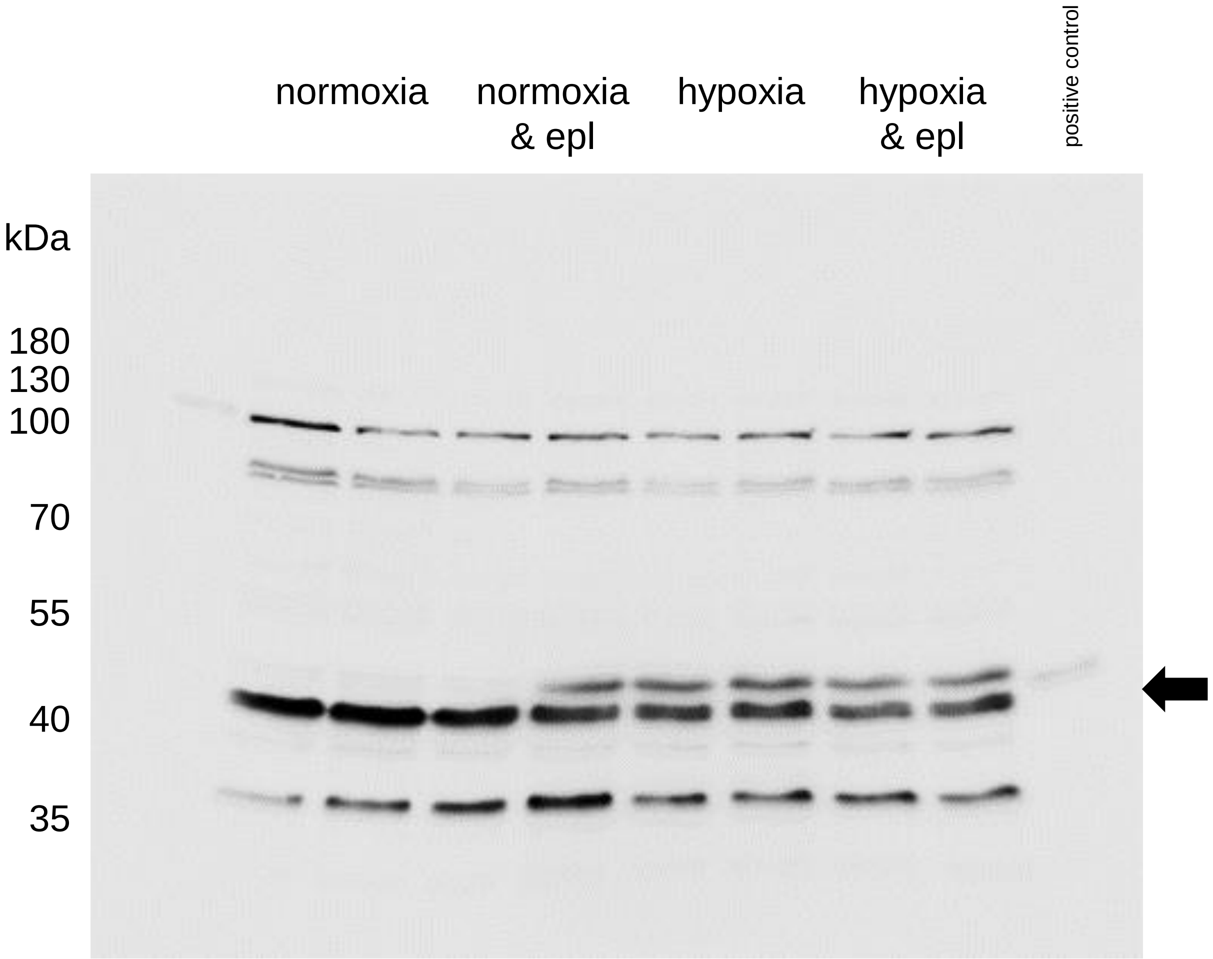

1. AB: PDK4 (1:1000) Boston
2. AB: anti-Rabbit (CellSignaling) 1:2000

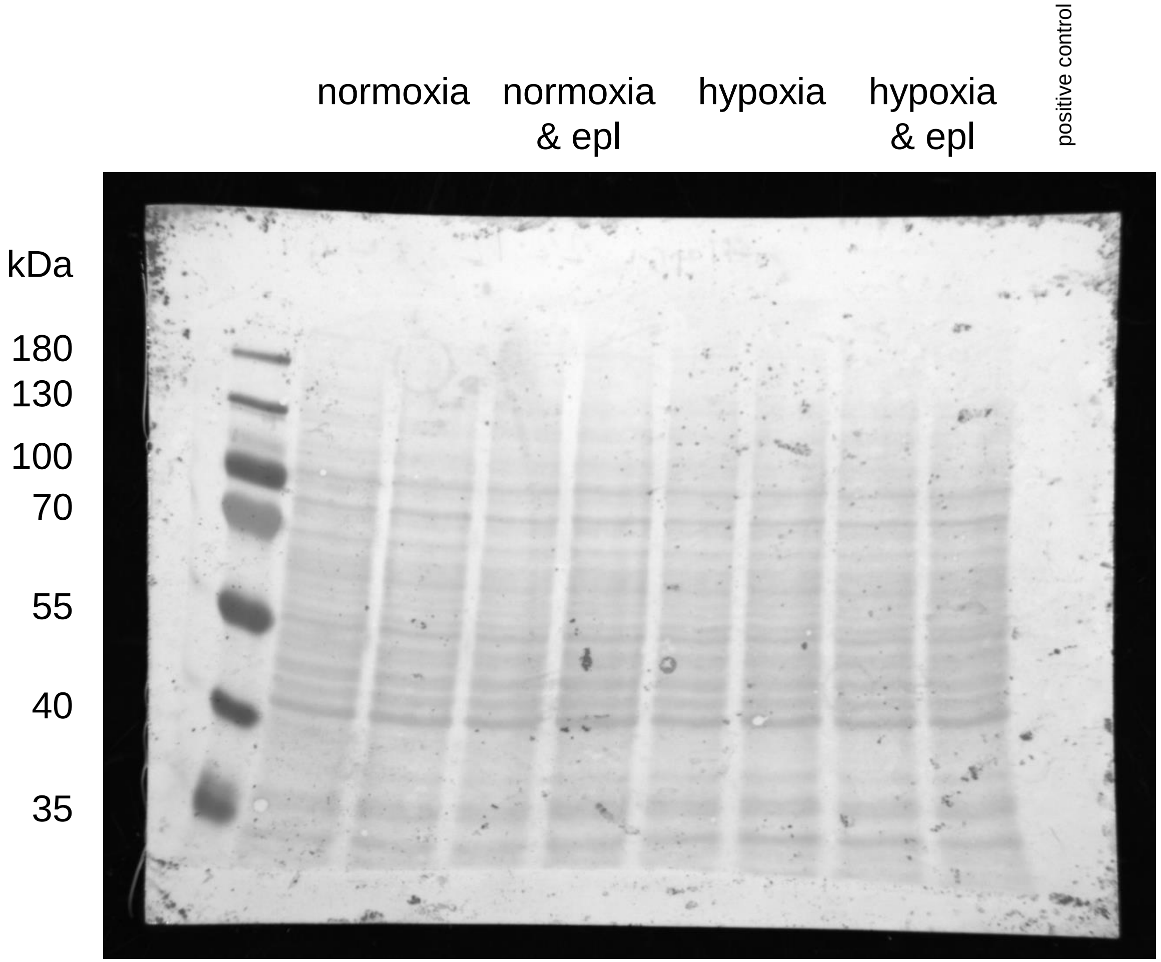

Ponceau

cell type: HepG2, passage x+6
target protein: PDK4
WB date: 07.03.2023
Incubation: normoxia, normoxia & epl, hypoxia, hypoxia & epl

date of experiment: 25.01.2023
MW: ~46 kDa
Marker : PageRuler (Thermo Fisher Scientific)
duration: 24h

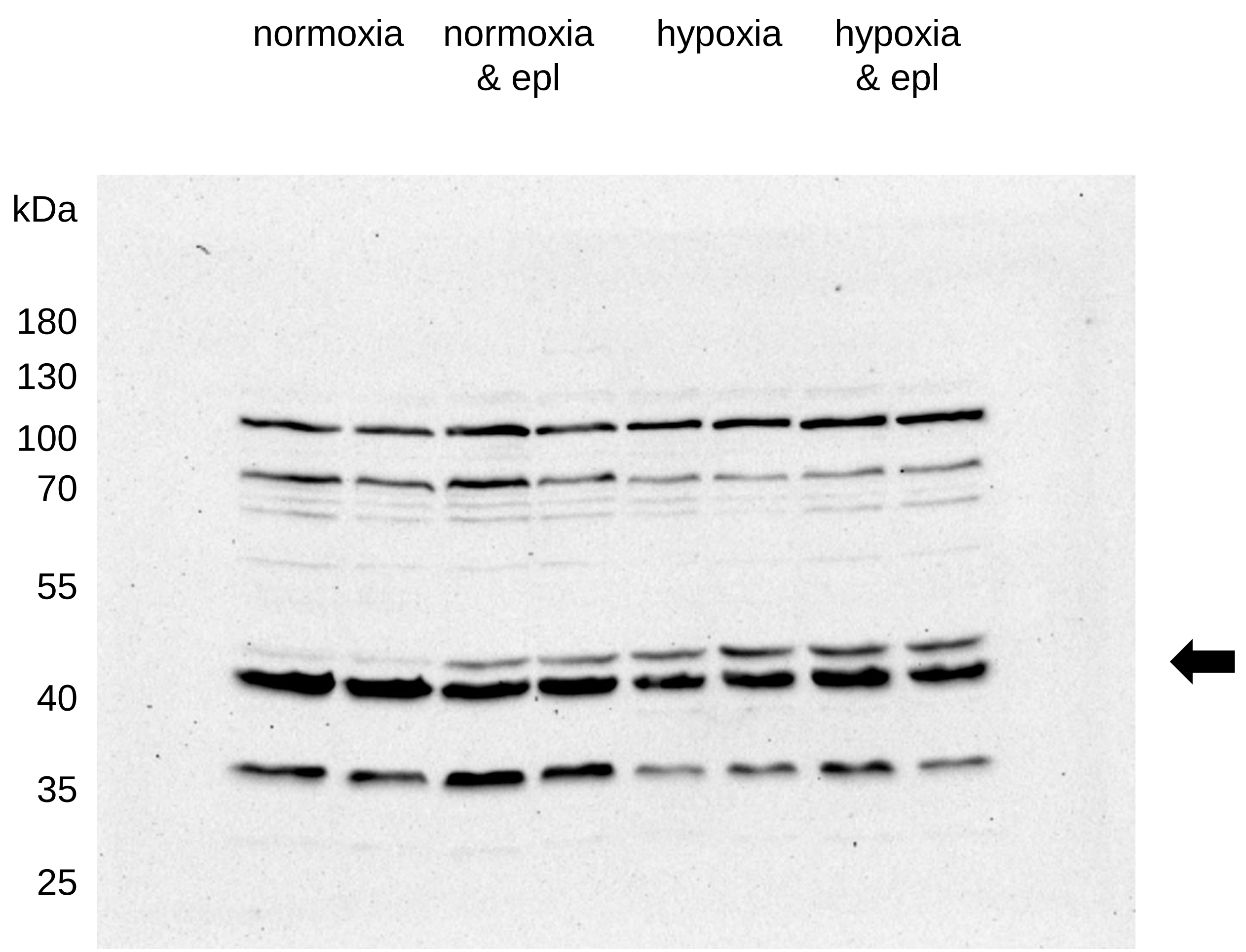

1. AB: PDK4 (1:1000) Boston
2. AB: anti-Rabbit (CellSignaling) 1:2000

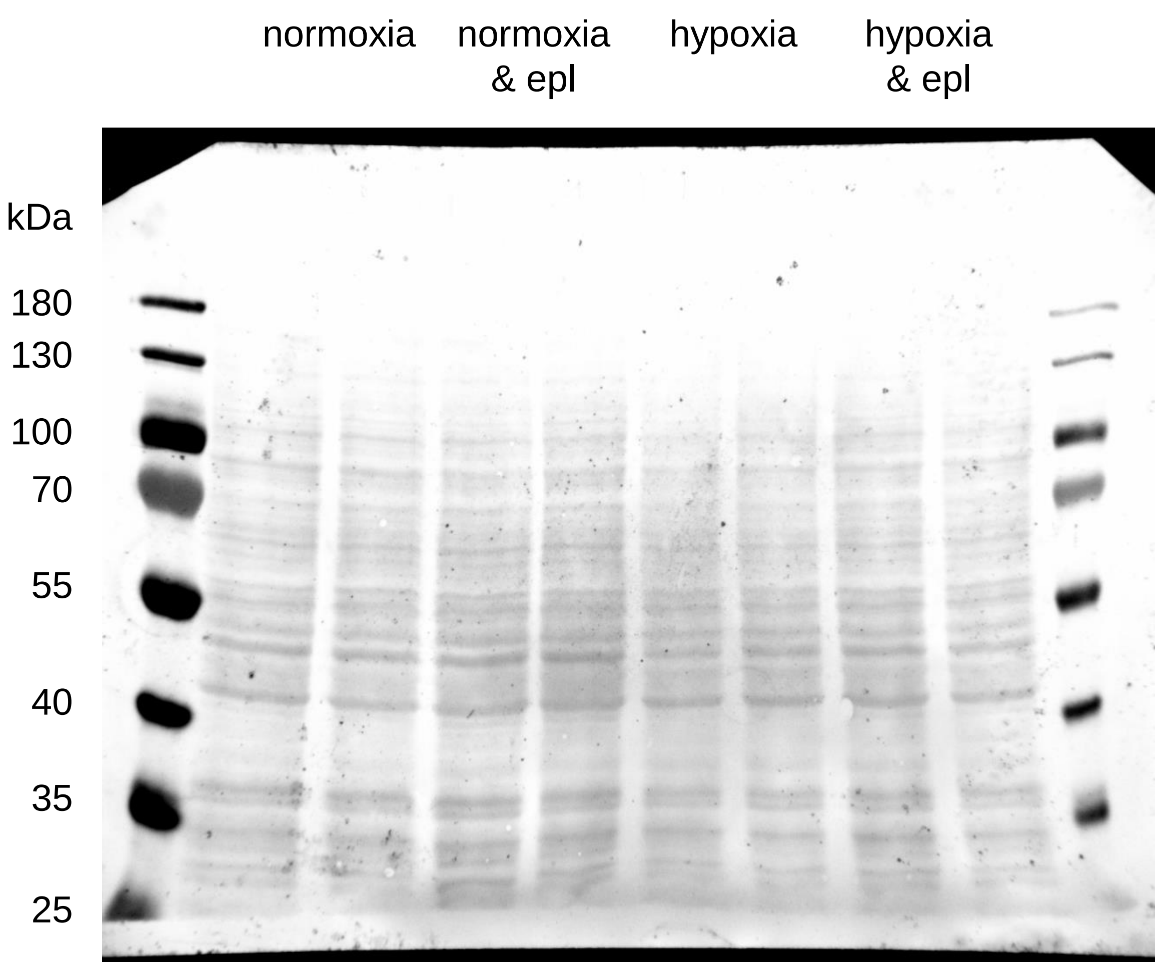

Ponceau

cell type: HepG2, passage x+11
target protein: ABCC2
WB date: 15.03.2023
incubation: normoxia, normoxia & epl, hypoxia, hypoxia & epl

date of experiment: 22.12.2022
MW: ~250 kDa
Marker : PageRuler (Thermo Fisher Scientific)
duration: 24h

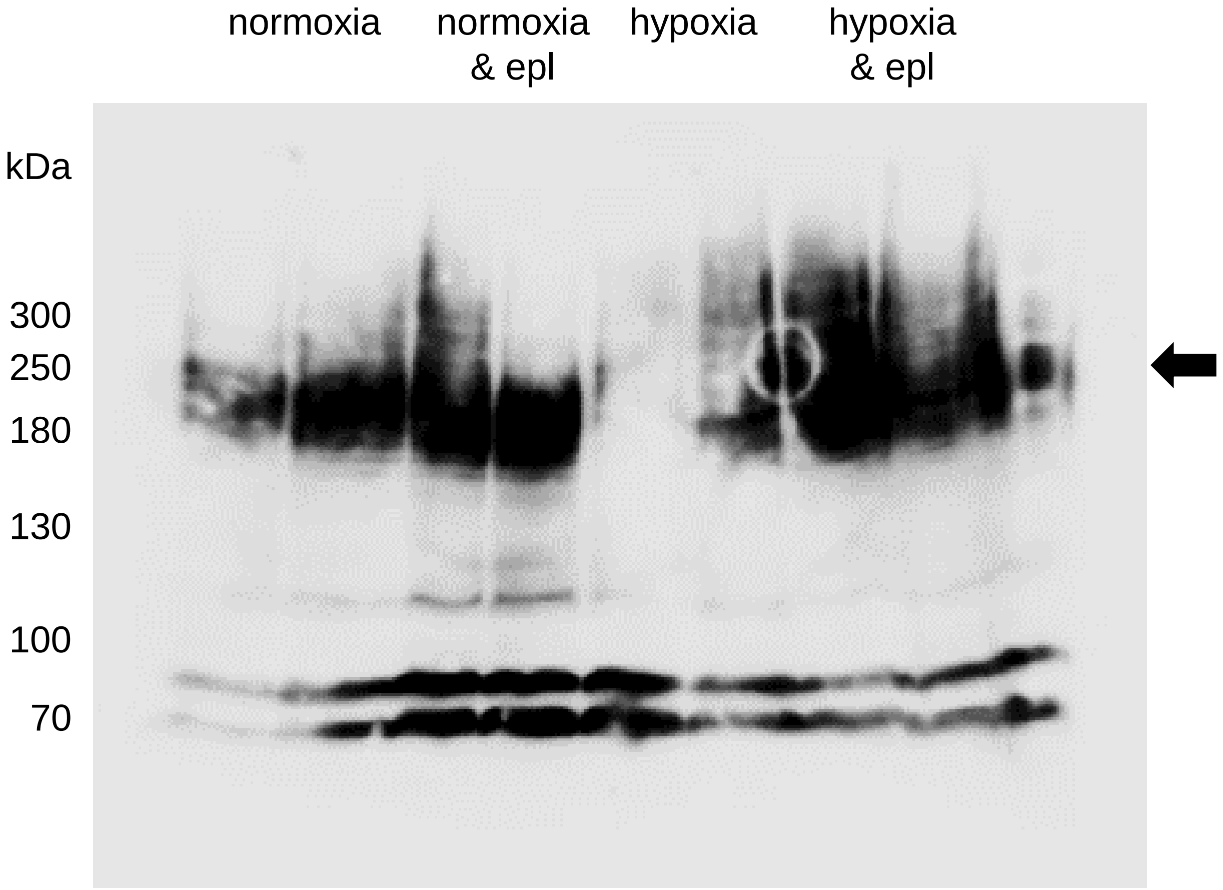

1. AB: ABCC2 (1:1000) #4446; Cell Signaling Technology
2. AB: anti-Rabbit (CellSignaling) 1:2000

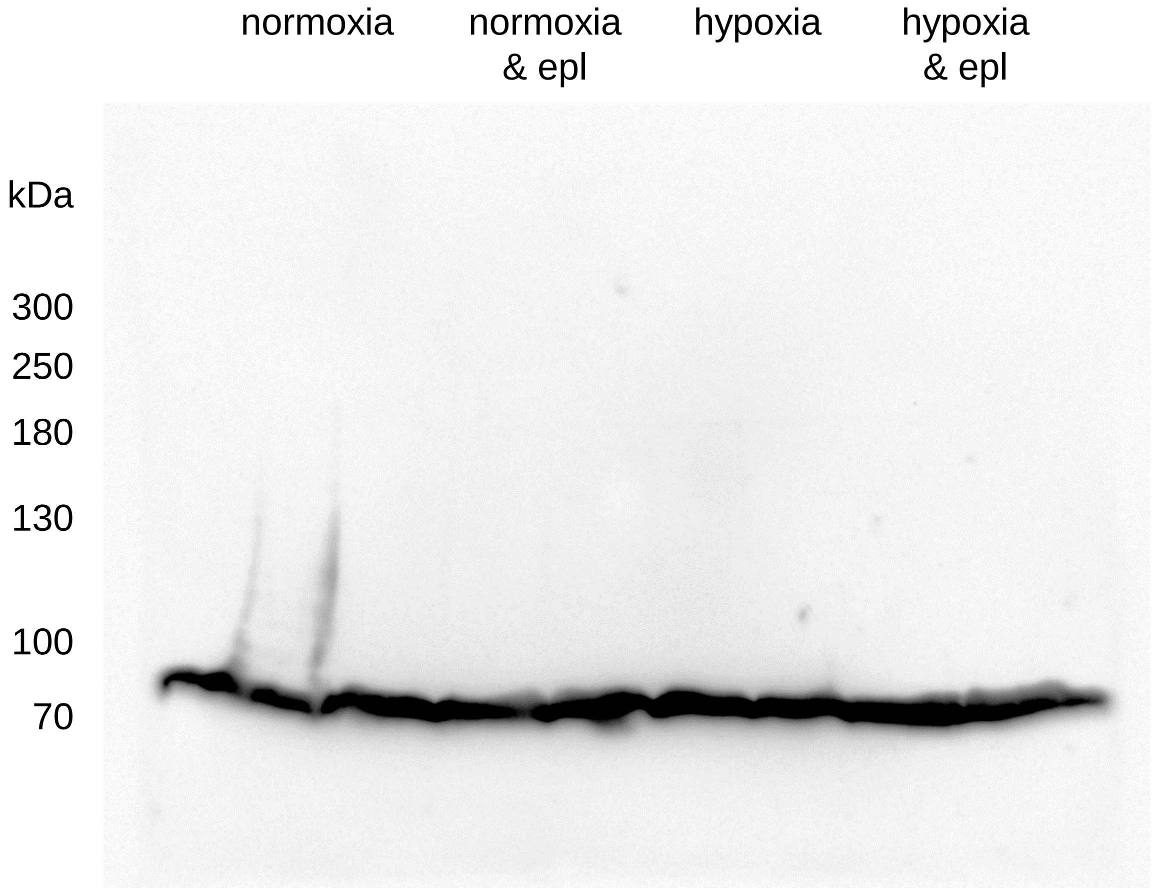

1. AB: HSP90 (1:2000) Cell Signalling # 4874; Cell Signalling
2. AB anti-Rabbit (CellSignaling) 1:2000

Cell type: HepG2, passage x+12 **date of experiment:** 23.12.2022
target protein: ABCC2 **MW:** ~250 kDa
WB date: 15.03.2023 **Marker :** PageRuler (Thermo Fisher Scientific)
Incubation: normoxia, normoxia & epl, hypoxia, hypoxia & epl **duration:** 24h

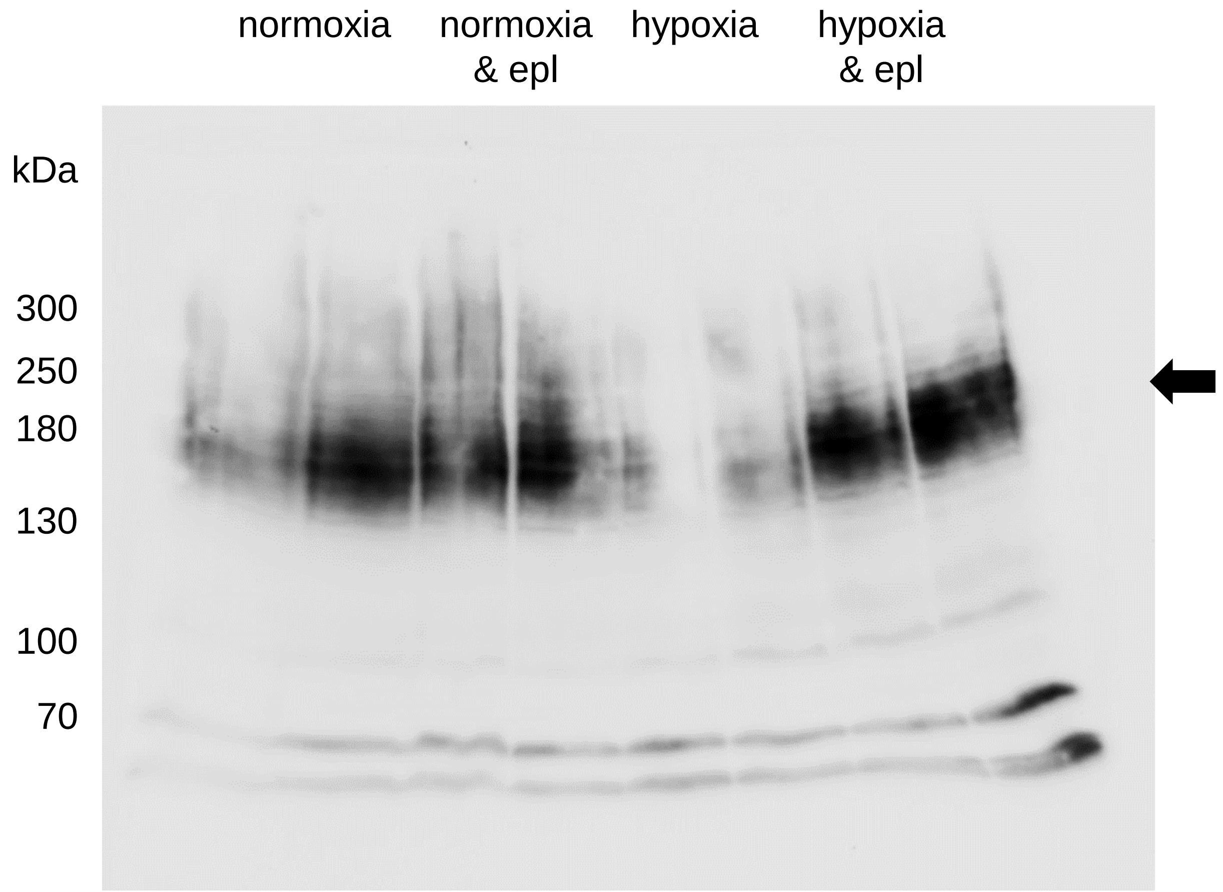

1. AB: ABCC2 (1:1000) #4446; Cell Signaling Technology
2. AB anti-Rabbit (CellSignaling) 1:2000

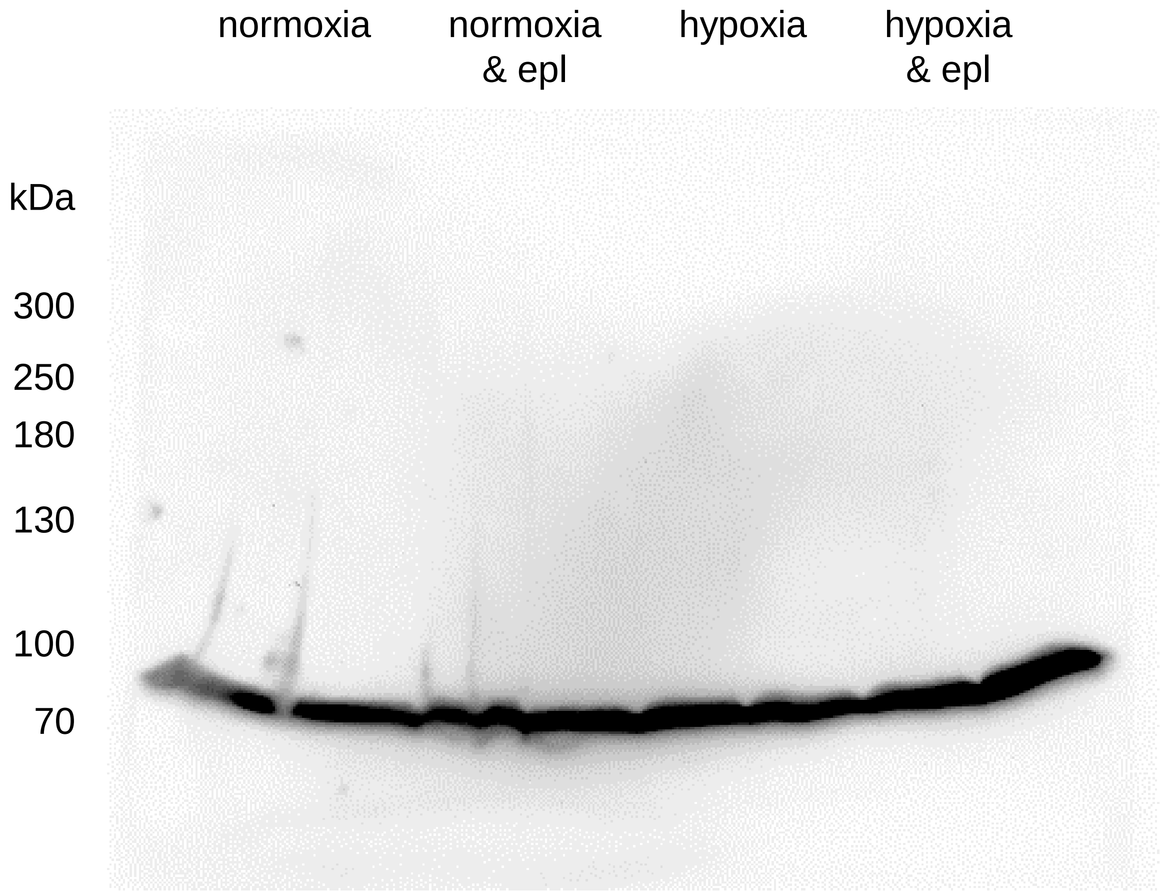

1. AB: HSP90 (1:2000) Cell Signalling # 4874; Cell Signalling
2. AB: anti-Rabbit (CellSignaling) 1:2000

Cell type: HepG2, passage x+9
target protein: ABCC2
WB date: 30.11.2022
Incubation: normoxia, normoxia & epl, hypoxia, hypoxia & epl

date of experiment: 25.11.2022
MW: ~250 kDa
Marker : PageRuler (Thermo Fisher Scientific)
duration: 24h

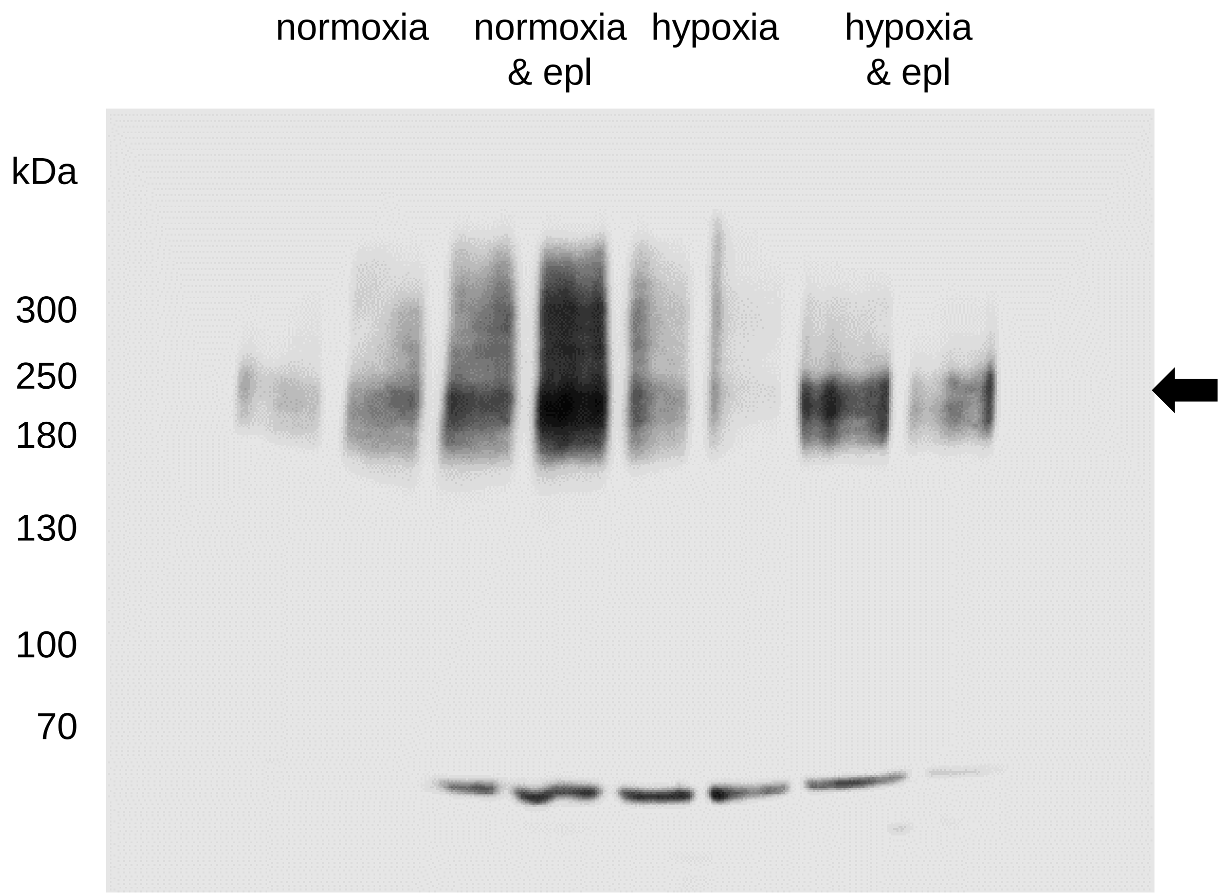

1. AB: ABCC2 (1:1000) #4446; Cell Signaling Technology
2. AB: anti-Rabbit (CellSignaling) 1:2000

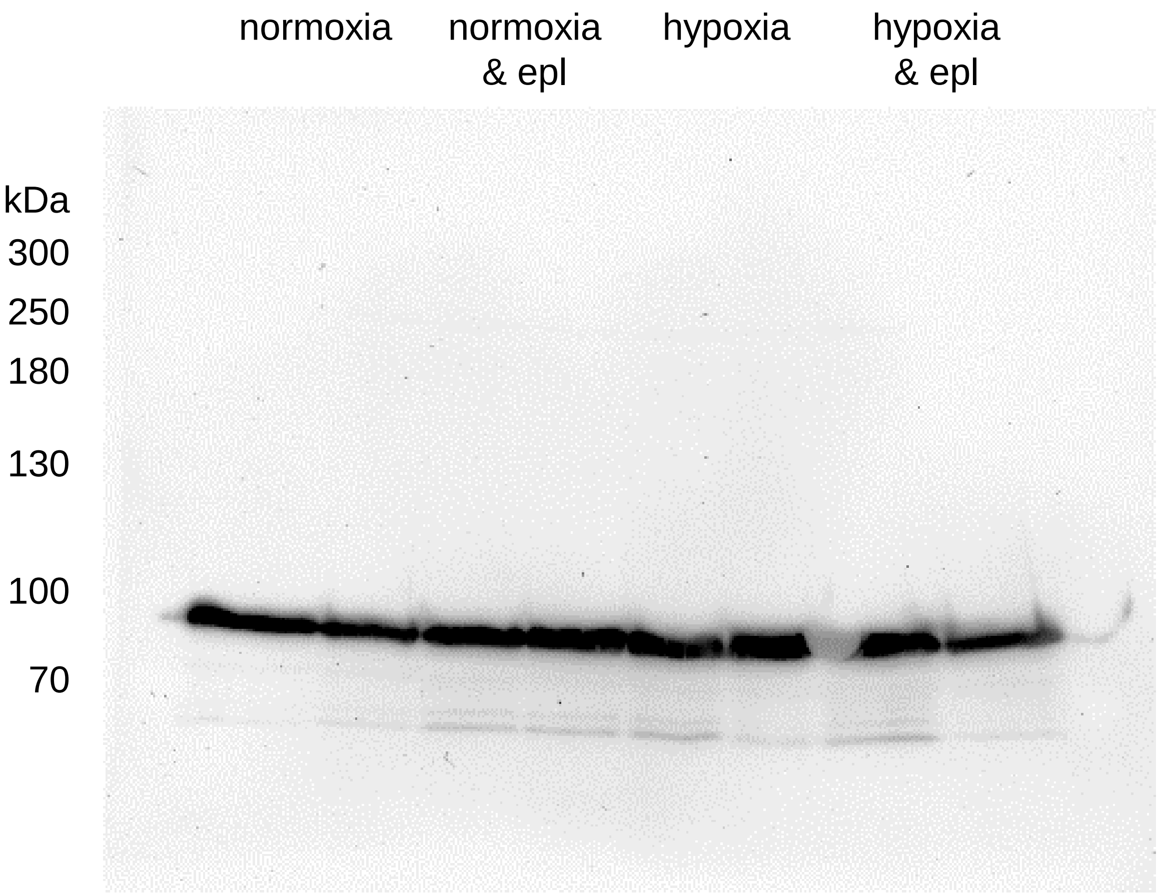

1. AB: HSP90 (1:2000) Cell Signalling # 4874; Cell Signalling
2. AB: anti-Rabbit (CellSignaling) 1:2000

cell type: HepG2, passage x+19
target protein: ABCC2
WB date: 10.11.2022
incubation: normoxia, normoxia & epl, hypoxia, hypoxia & epl

date of experiment: 14.10.2022
MW: ~250 kDa
Marker : PageRuler (Thermo Fisher Scientific)
duration: 24h

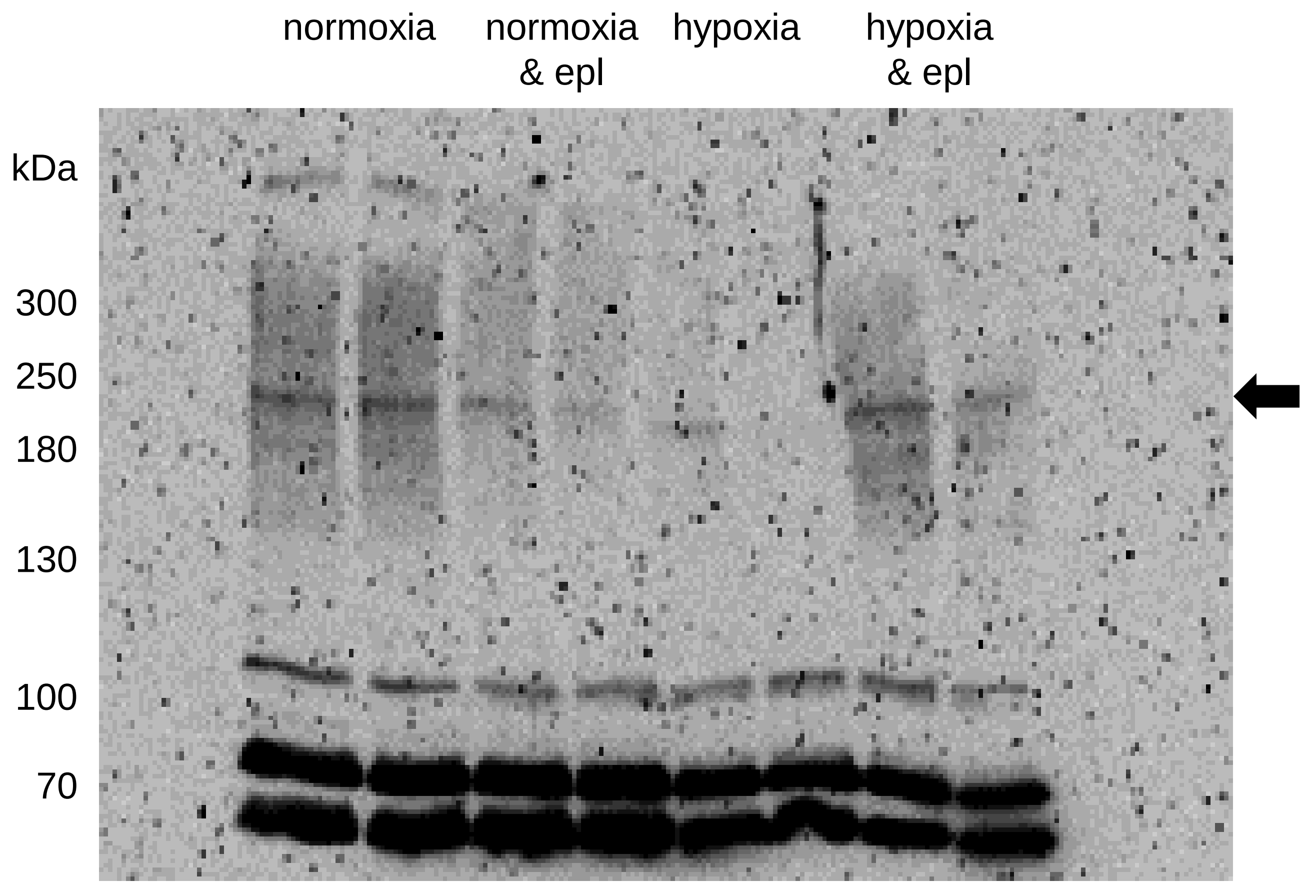

1. AB: ABCC2 (1:1000) #4446; Cell Signaling Technology
2. AB anti-Rabbit (CellSignaling) 1:2000

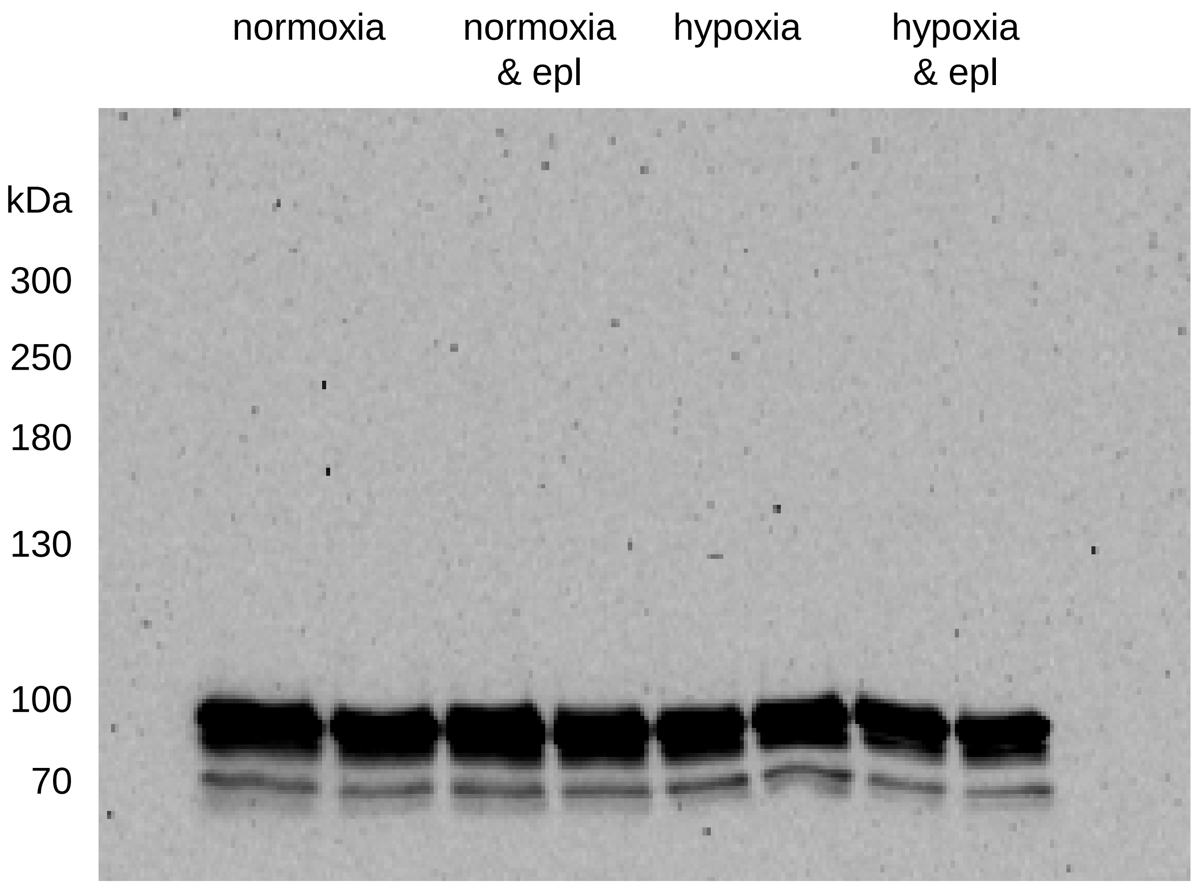

1. AB: HSP90 (1:2000) Cell Signalling # 4874; Cell Signalling
2. AB: anti-Rabbit (CellSignaling) 1:2000

cell type: HepG2, passage x+18
target protein: ABCC2
WB date: 10.11.2022
incubation: normoxia, normoxia & epl, hypoxia, hypoxia & epl

date of experiment: 14.10.2022
MW: ~250 kDa
Marker : PageRuler (Thermo Fisher Scientific)
duration: 24h

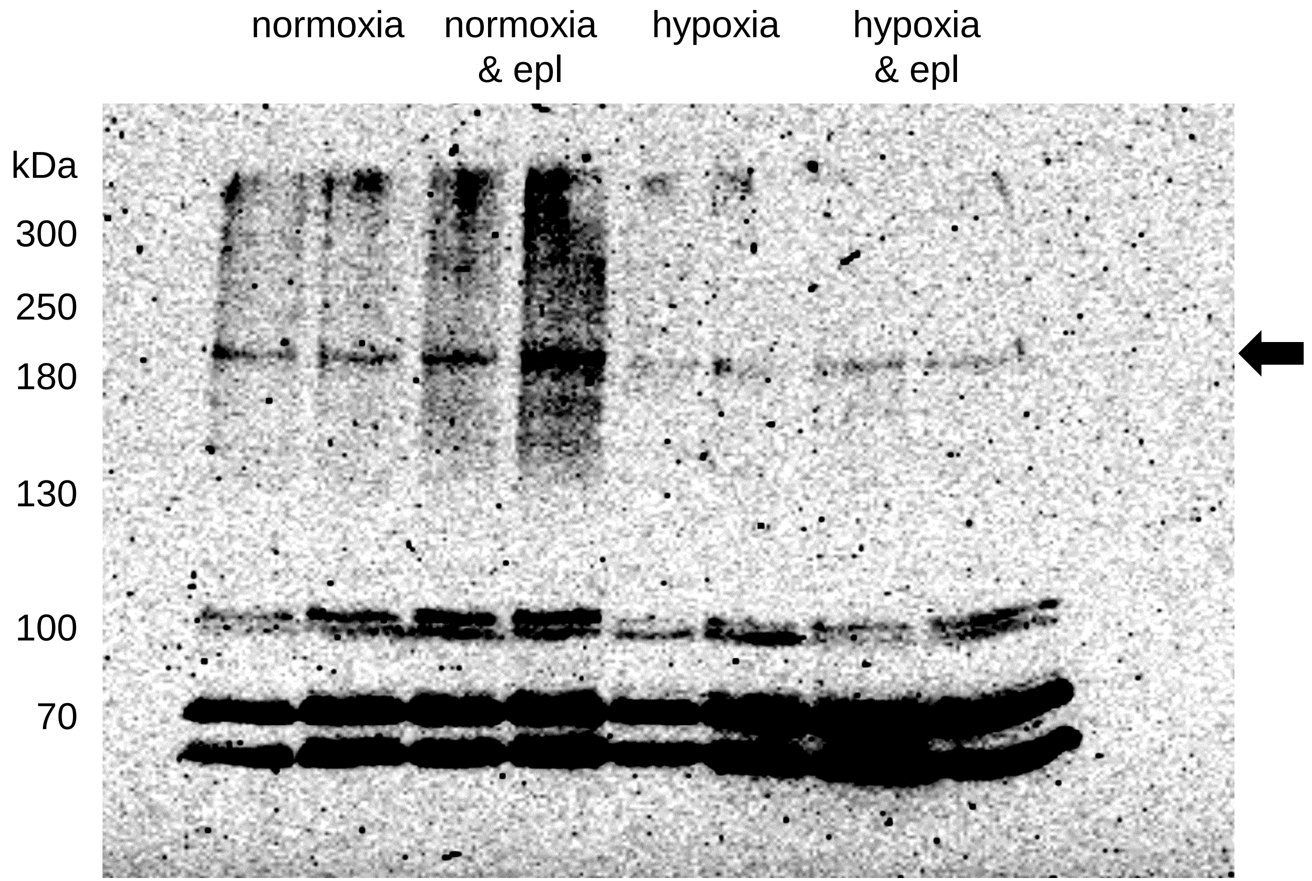

1. AB: ABCC2 (1:1000) #4446; Cell Signaling Technology
2. AB anti-Rabbit (CellSignaling) 1:2000

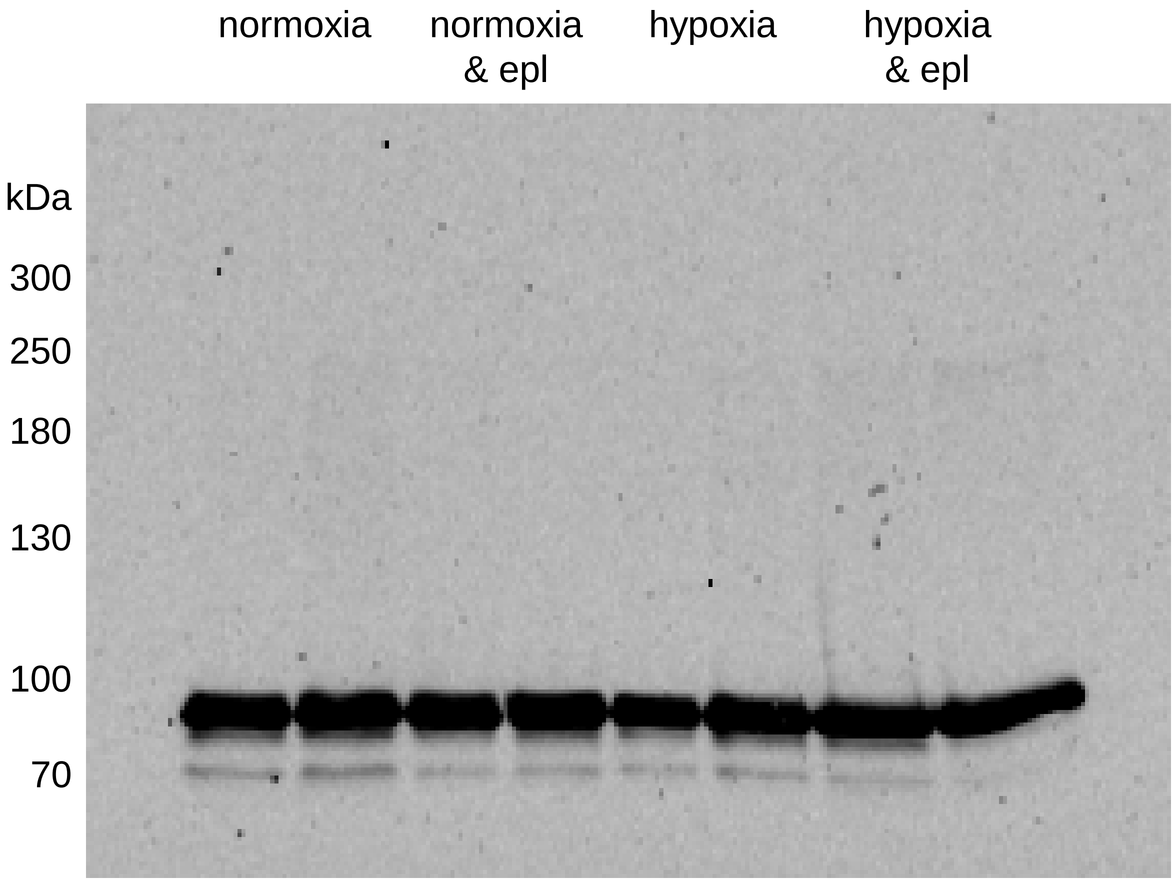

1. AB: HSP90 (1:2000) Cell Signalling # 4874; Cell Signalling
2. AB: anti-Rabbit (CellSignaling) 1:2000

Cell type: HepG2, passage x+7
target protein: AMACR
WB date: 04.01.2023
Incubation: normoxia, normoxia & epl, hypoxia, hypoxia & epl

date of experiment: 16.12.2022
MW: ~42 kDa
Marker : PageRuler (Thermo Fisher Scientific)
duration: 24h

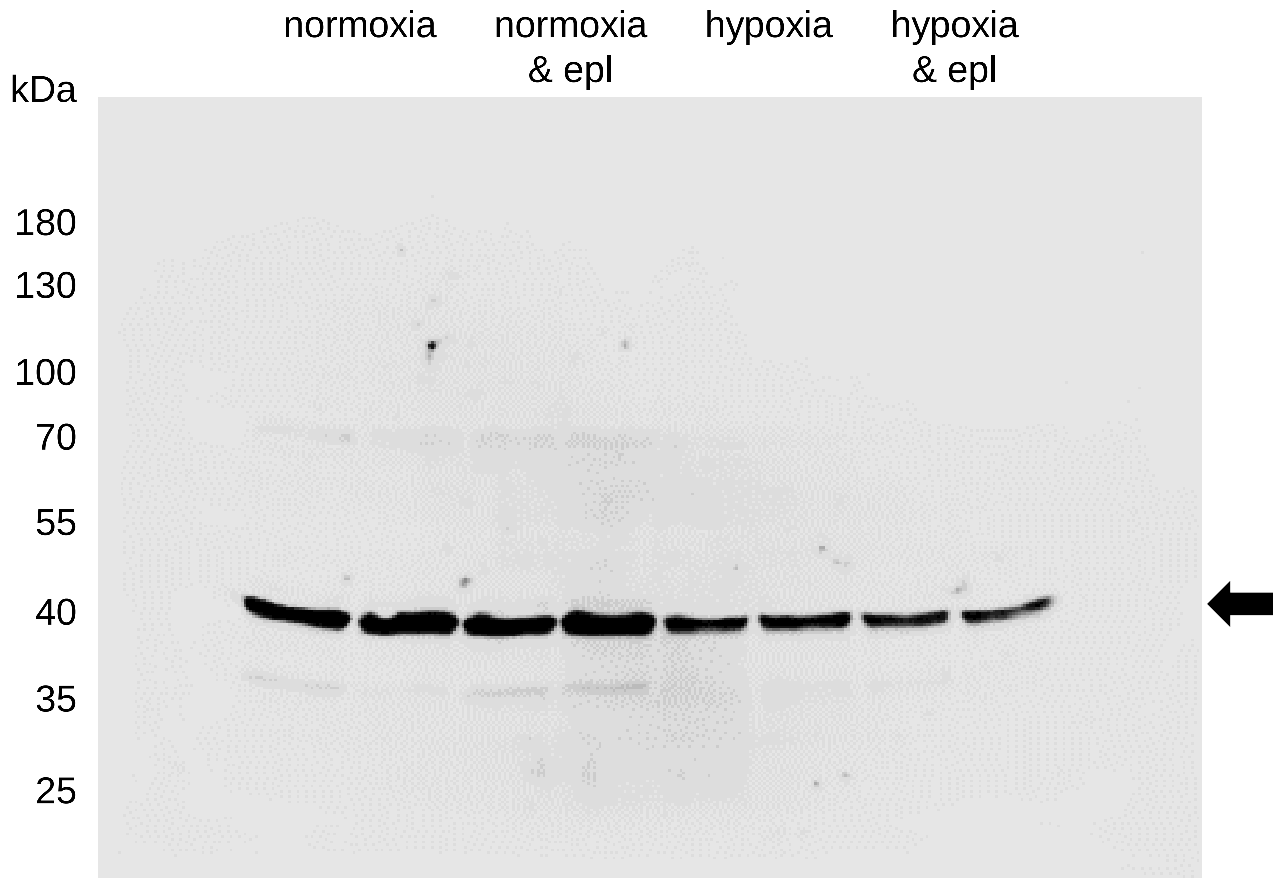

1. AB:AMACR (1:2000) #3207, Cell Signaling Technology
2. AB: anti-Mouse(CellSignaling) 1:2000

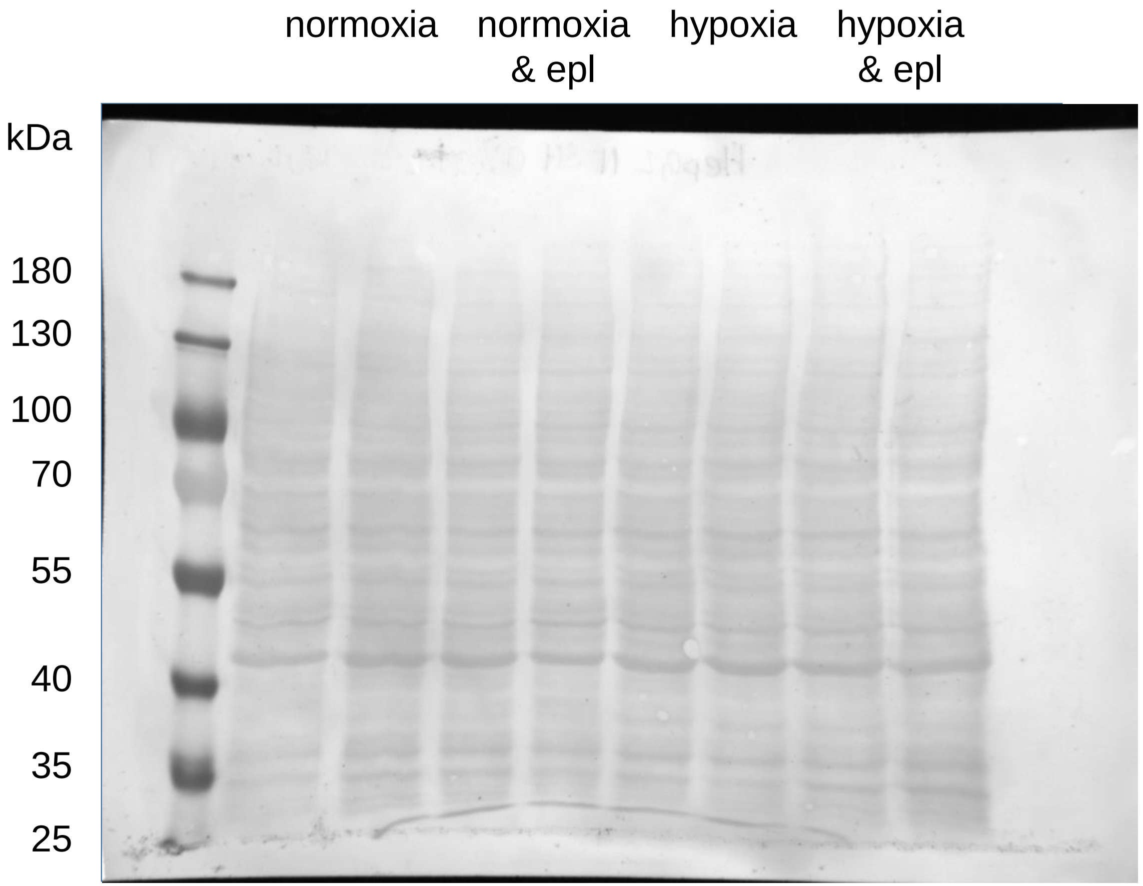

Ponceau

cell type: HepG2, passage x+8

target protein: AMACR

WB date: 05.01.2023

incubation: normoxia, normoxia & epl, hypoxia, hypoxia & epl

date of experiment: 16.12.2022

MW: ~42 kDa

Marker : PageRuler (Thermo Fisher Scientific)

duration: 24h

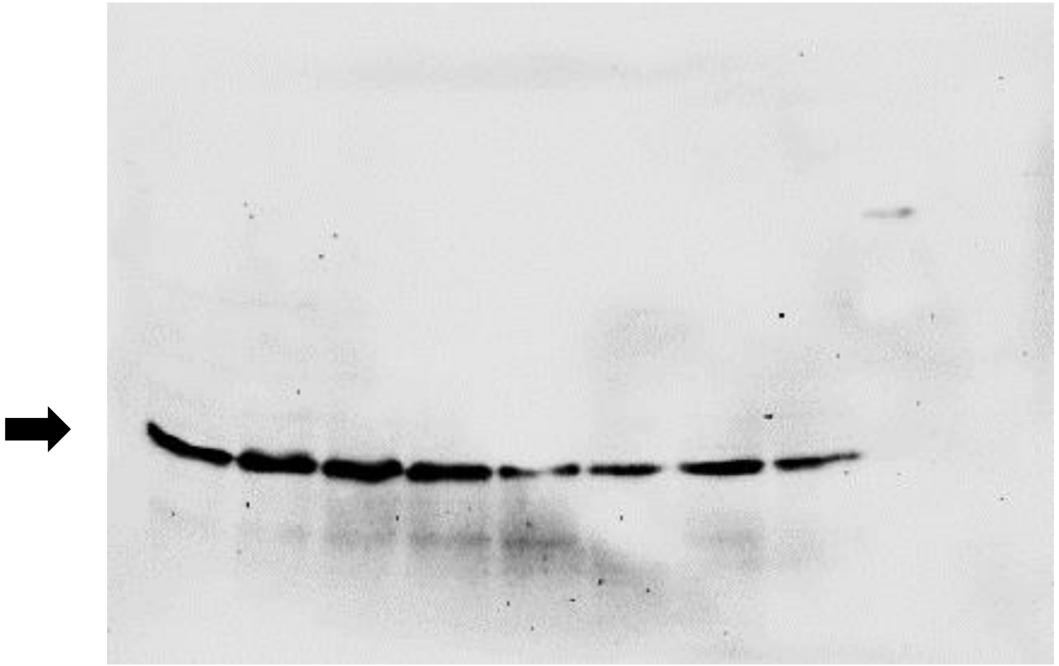

1. AB: AMACR (1:2000) #3207, Cell Signaling Technology
2. AB: anti-Mouse(CellSignaling) 1:2000

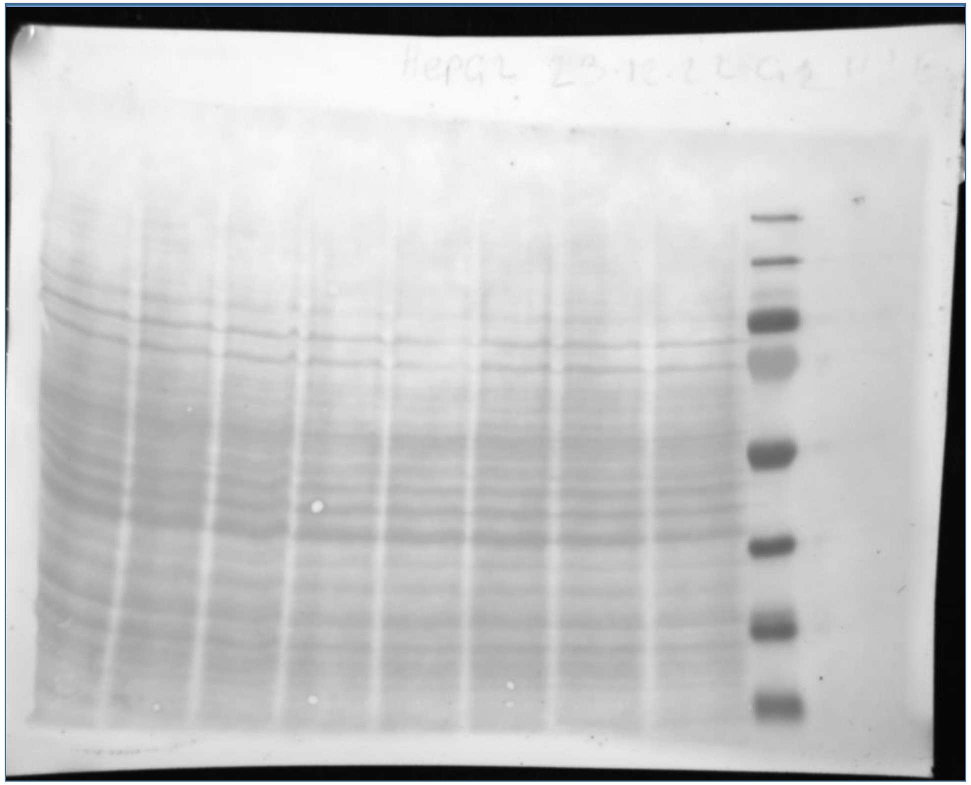

Ponceau

Cell type: HepG2, passage x+11 **date of experiment:** 22.12.2022
target protein: AMACR **MW:** ~42kDa
WB date: 04.01.2023 **Marker :** PageRuler (Thermo Fisher Scientific)
Incubation: normoxia, normoxia & epl, hypoxia, hypoxia & epl **duration:** 24h

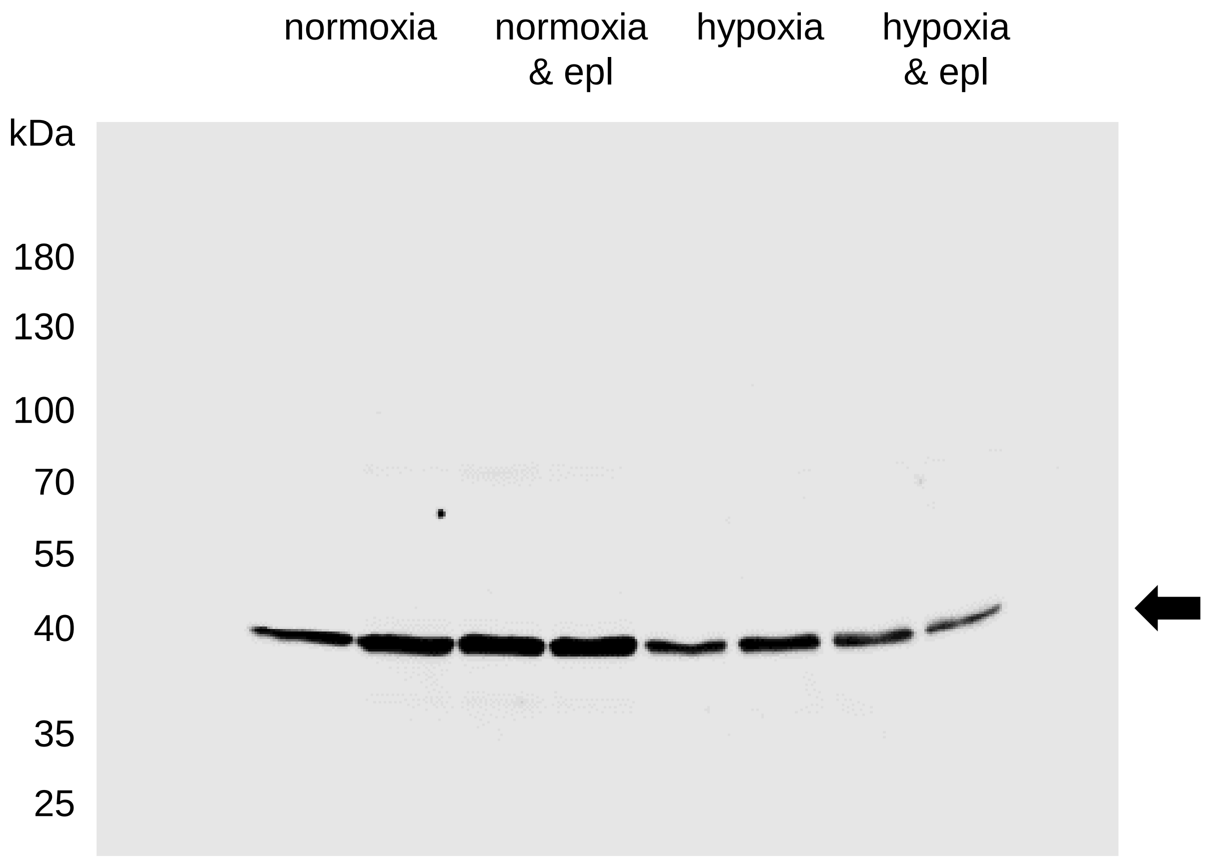

1. AB:AMACR (1:2000) #3207, Cell Signaling Technology
2. AB: anti-Mouse(CellSignaling) 1:2000

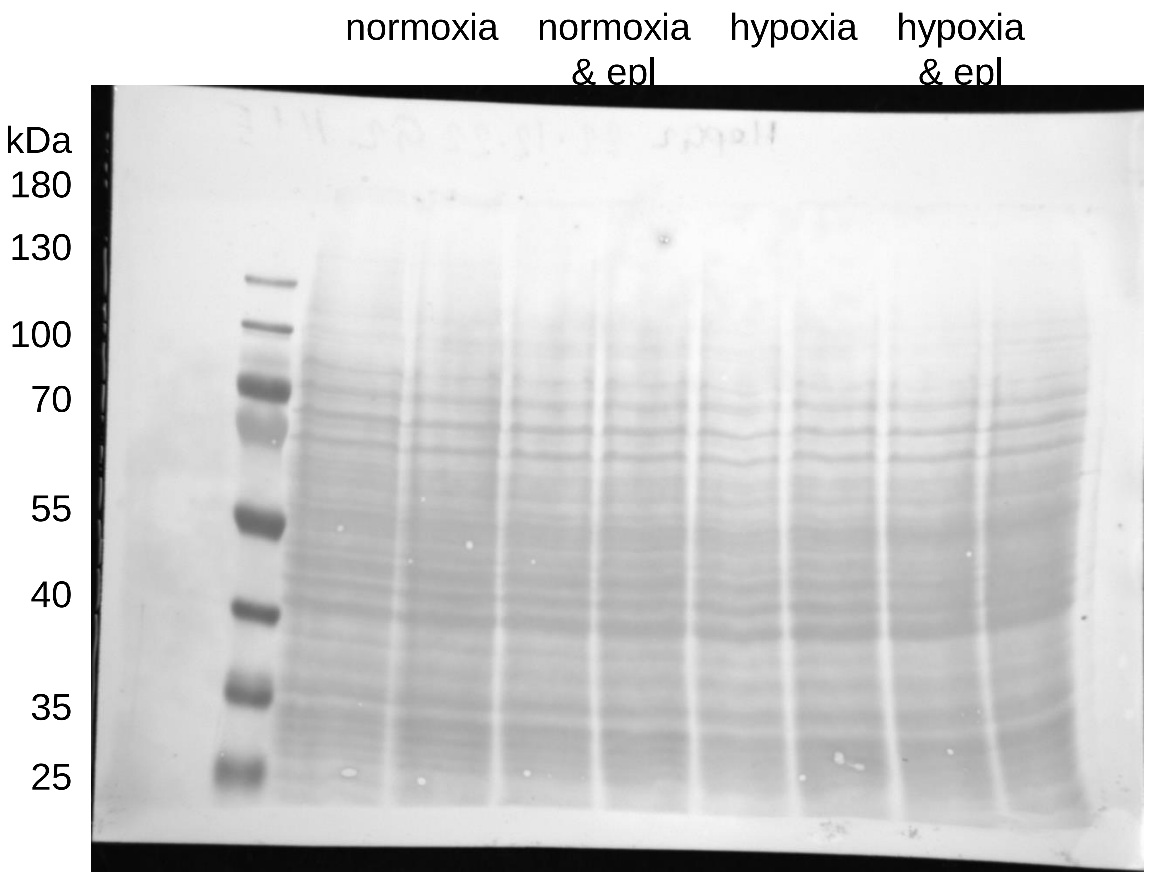

Ponceau

cell type: HepG2, passage x+12
target protein: AMACR
WB date: 05.01.2023
Incubation: normoxia, normoxia & epl, hypoxia, hypoxia & epl

date of experiment: 23.12.2023
MW: ~42 kDa
Marker : PageRuler (Thermo Fisher Scientific)
duration: 24h

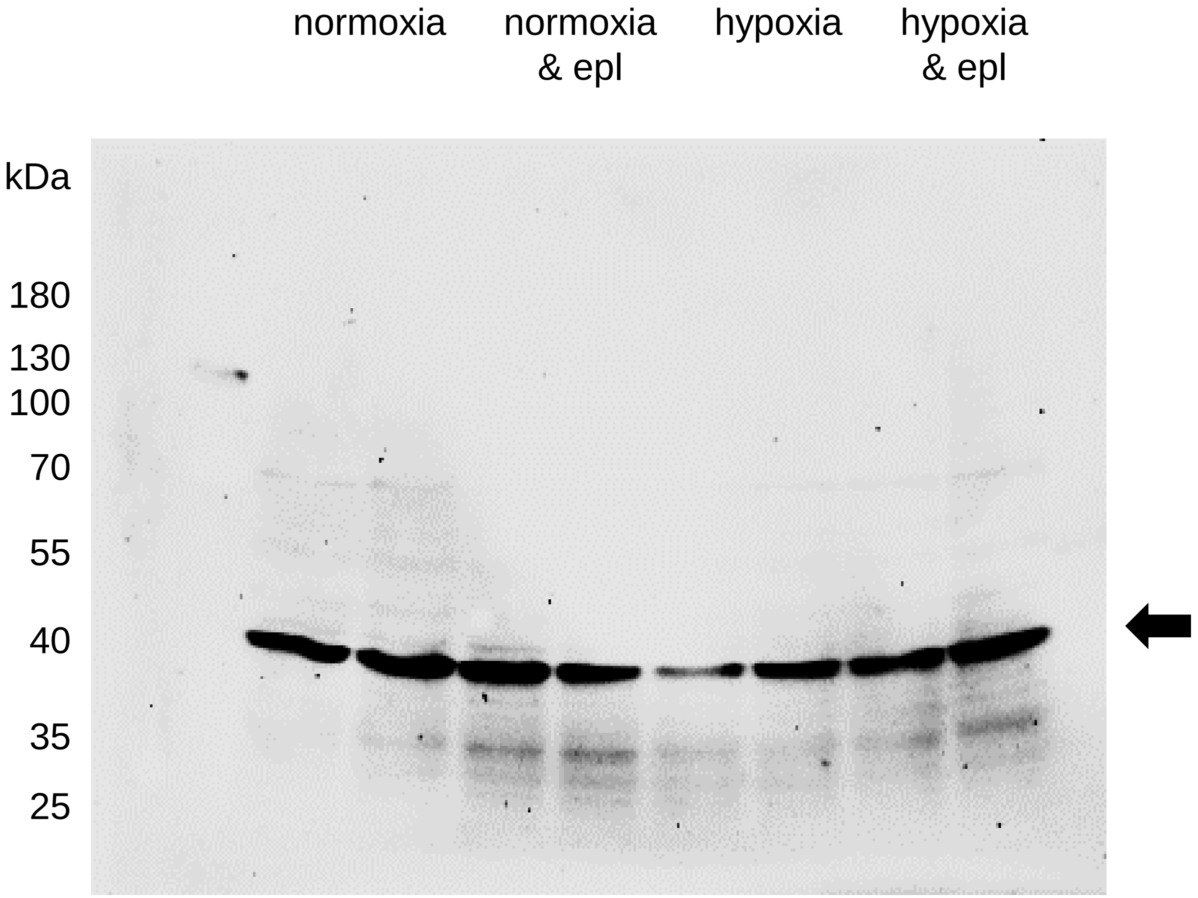

1. AB: AMACR (1:2000) #3207, Cell Signaling Technology
2. AB: anti-Mouse(CellSignaling) 1:2000

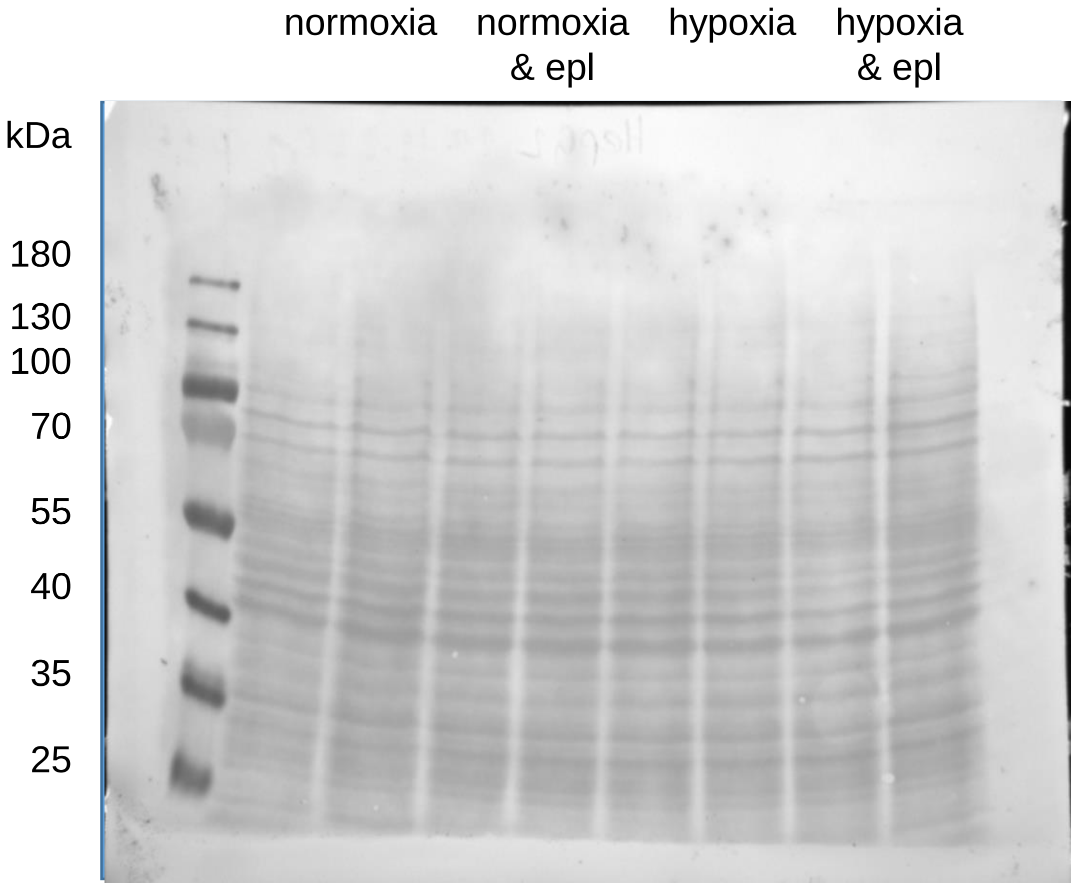

Ponceau

cell type: HepG2, passage x+6 **date of experiment:** 25.01.2023
target protein: AMACR **MW:** ~42kDa
WB date: 06.03.2023 **Marker :** PageRuler (Thermo Fisher Scientific)
incubation: normoxia, normoxia & epl, hypoxia, hypoxia & epl **duration:** 24h

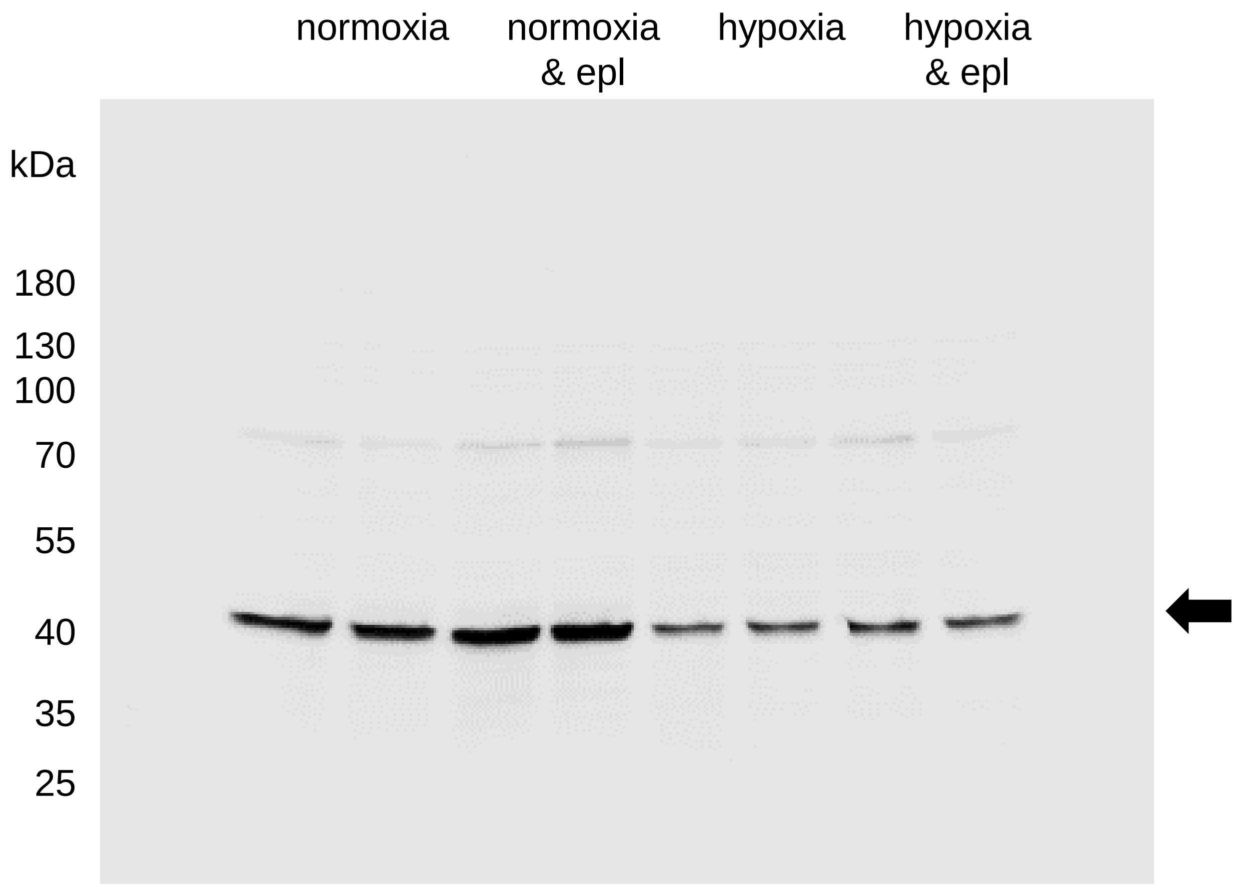

1. AB: AMACR (1:2000) #3207, Cell Signaling Technology
2. AB: anti-Mouse(CellSignaling) 1:2000

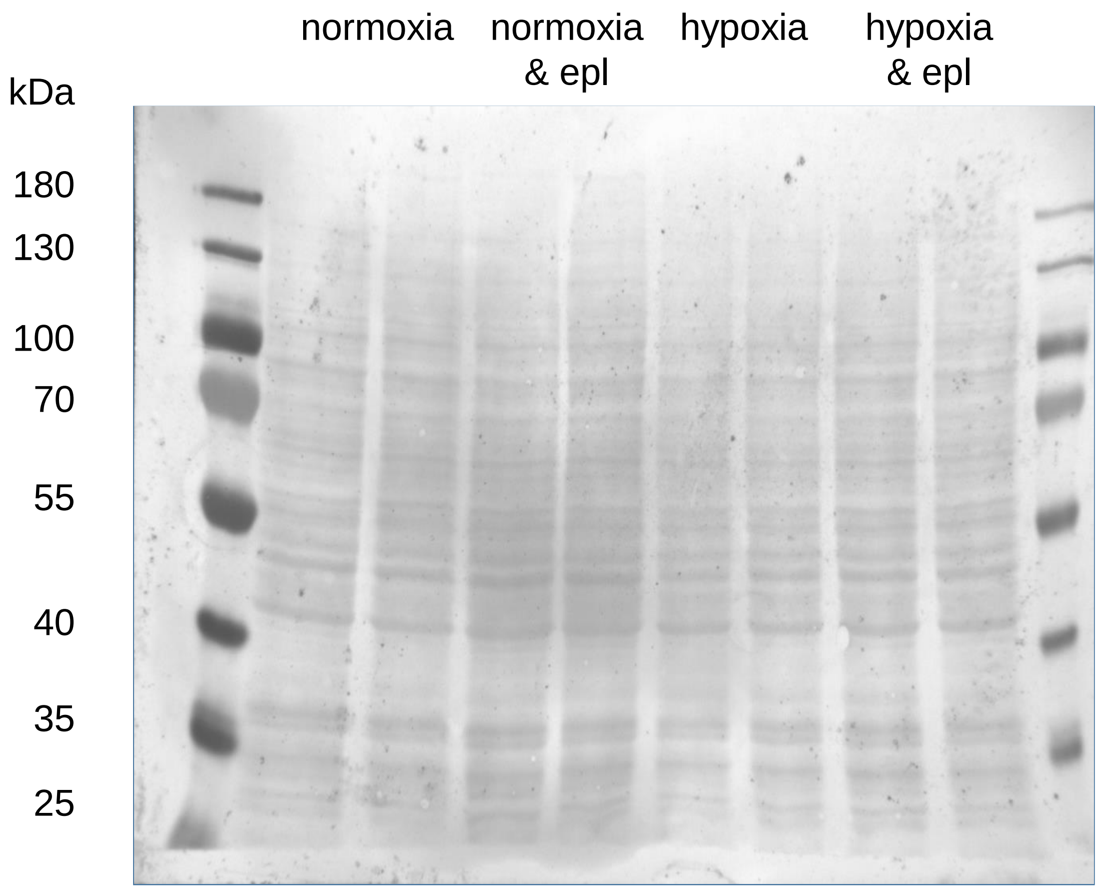

Ponceau

Cell type: HepG2, passage x+9
target protein: Lipin 1
WB date: 13.12.2022
Incubation: normoxia, normoxia & epl, hypoxia, hypoxia & epl

date of experiment: 25.11.2022
MW: ~130 kDa
Marker : PageRuler (Thermo Fisher Scientific)
duration: 24h

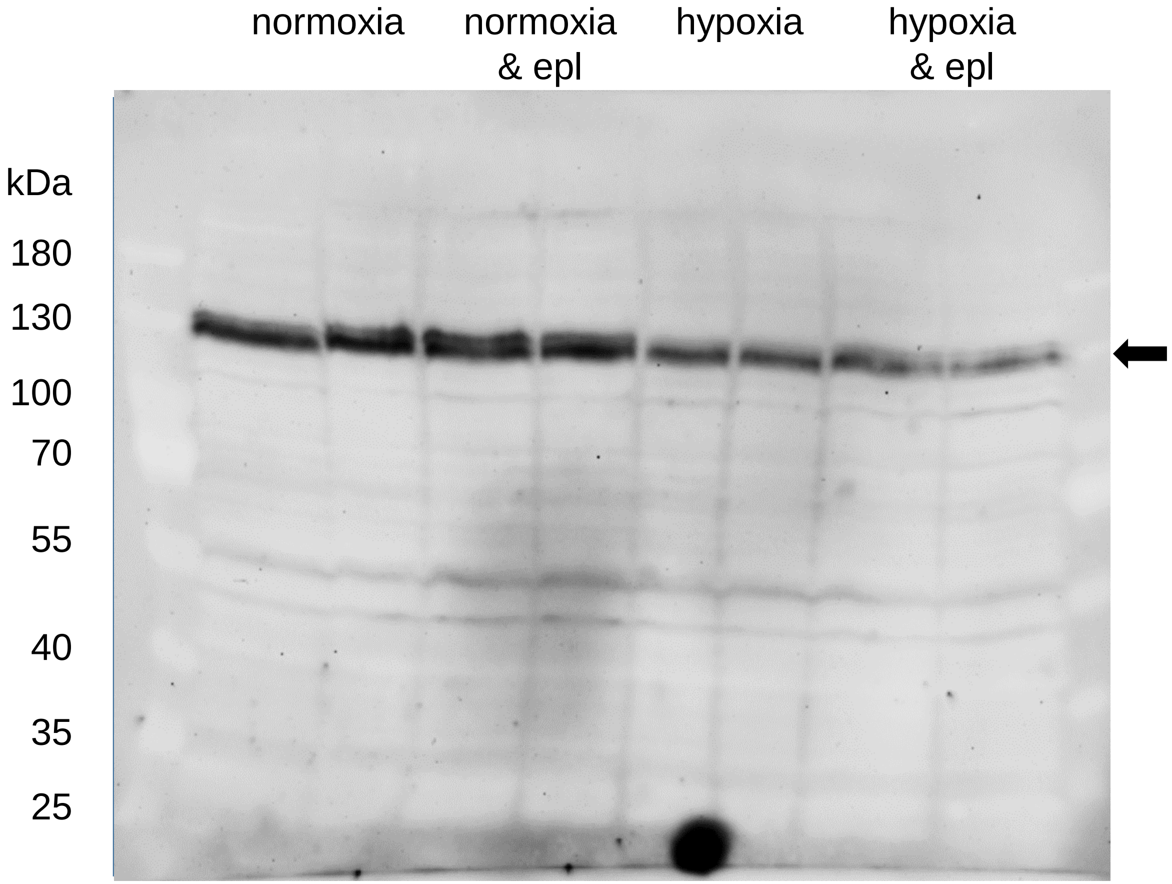

1. AB: Lipin (1:1000) ; #5195; Cell Signaling Technology
2. AB: anti-Rabbit (CellSignaling) 1:2000

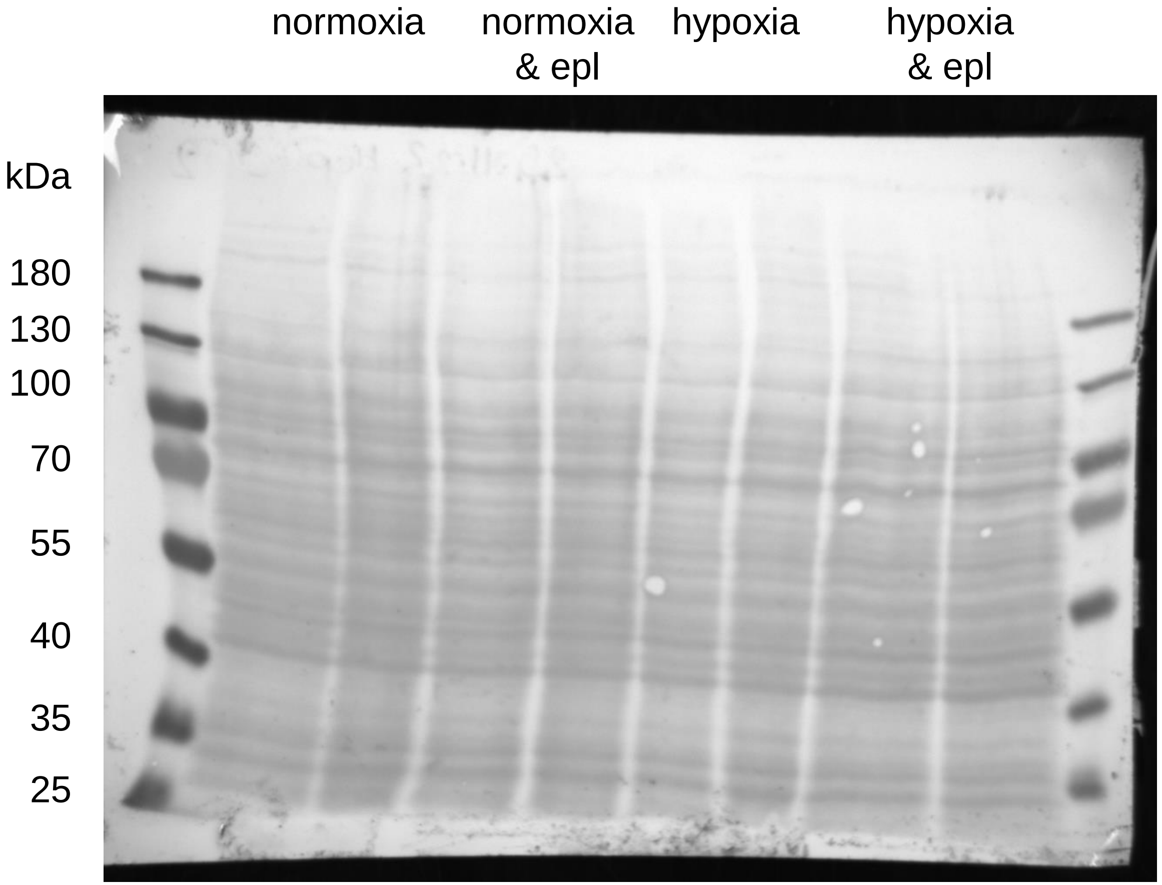

Ponceau

Cell type: HepG2, passage x+11

target protein: Lipin1

WB date: 15.11.2022

Incubation: normoxia, normoxia & epl, hypoxia, hypoxia & epl

date of experiment: 14.10.22

MW: ~130 kDa

Marker : PageRuler (Thermo Fisher Scientific)

duration: 24h

normoxia | normoxia & epl | hypoxia | hypoxia & epl

kDa

180
130
100
70
55
45
35
25

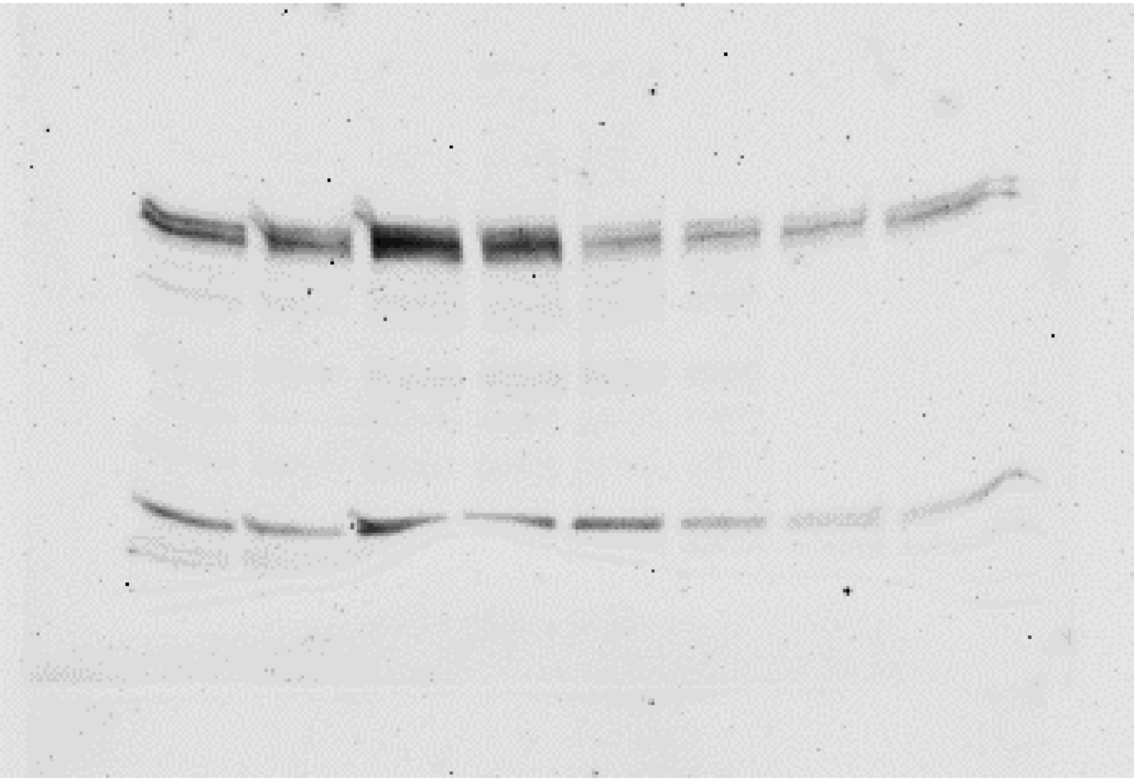

1. AB: Lipin (1:1000) ; #5195; Cell Signaling Technology
2. AB: anti-Rabbit (CellSignaling) 1:2000

normoxia | normoxia & epl | hypoxia | hypoxia & epl

kDa

180
130
100
70
55
40
35
25

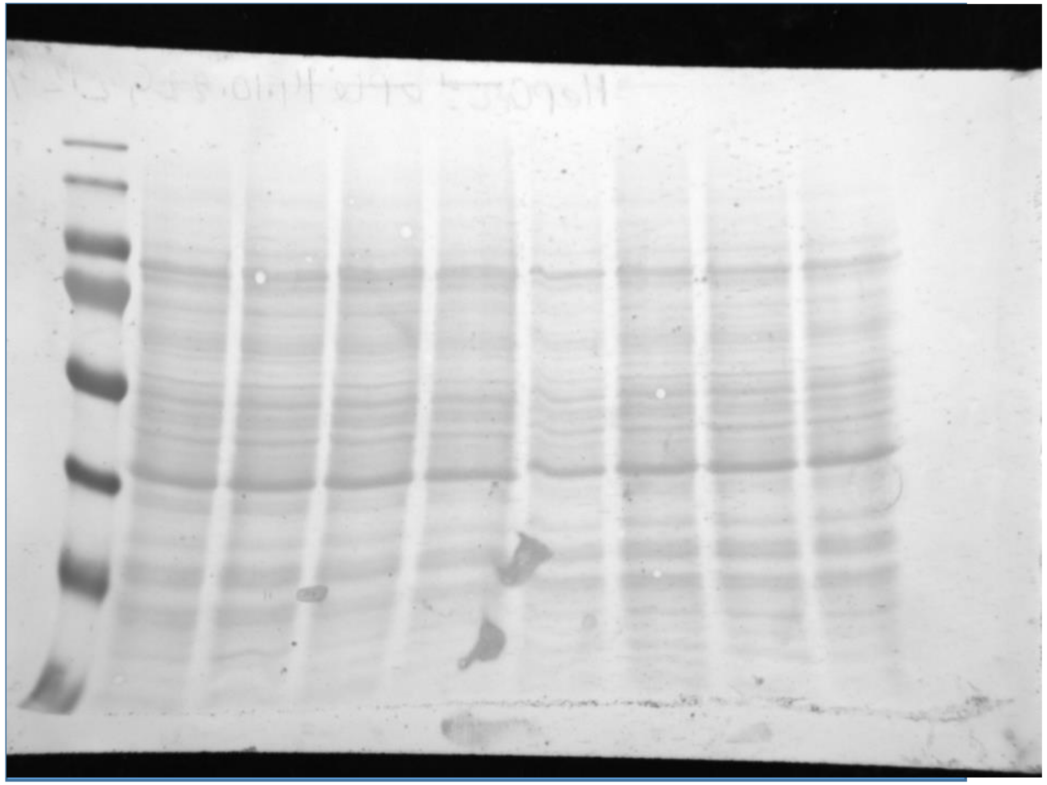

Ponceau

Cell type: HepG2, passage x+10

target protein: Lipin1

WB date: 23.09.2022

Incubation: normoxia, normoxia & epl, hypoxia, hypoxia & epl

date of experiment: 14.09.2020

MW: ~130kDa

Marker : PageRuler (Thermo Fisher Scientific)

duration: 24h

normoxia | normoxia & epl | hypoxia | hypoxia & epl

kDa 180 130 100 70 55 40 35 25

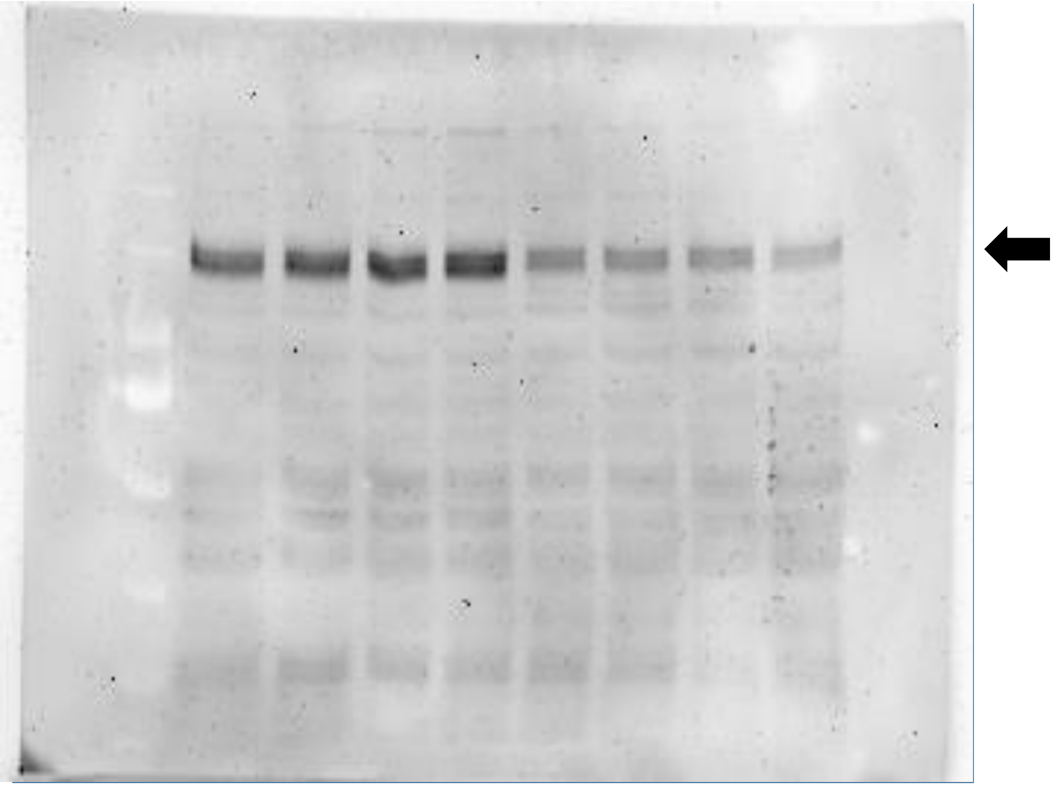

1. AB: Lipin (1:1000) ; #5195; Cell Signaling Technology
2. AB: anti-Rabbit (CellSignaling) 1:2000

normoxia | normoxia & epl | hypoxia | hypoxia & epl

kDa 180 130 100 70 55 40 35 25

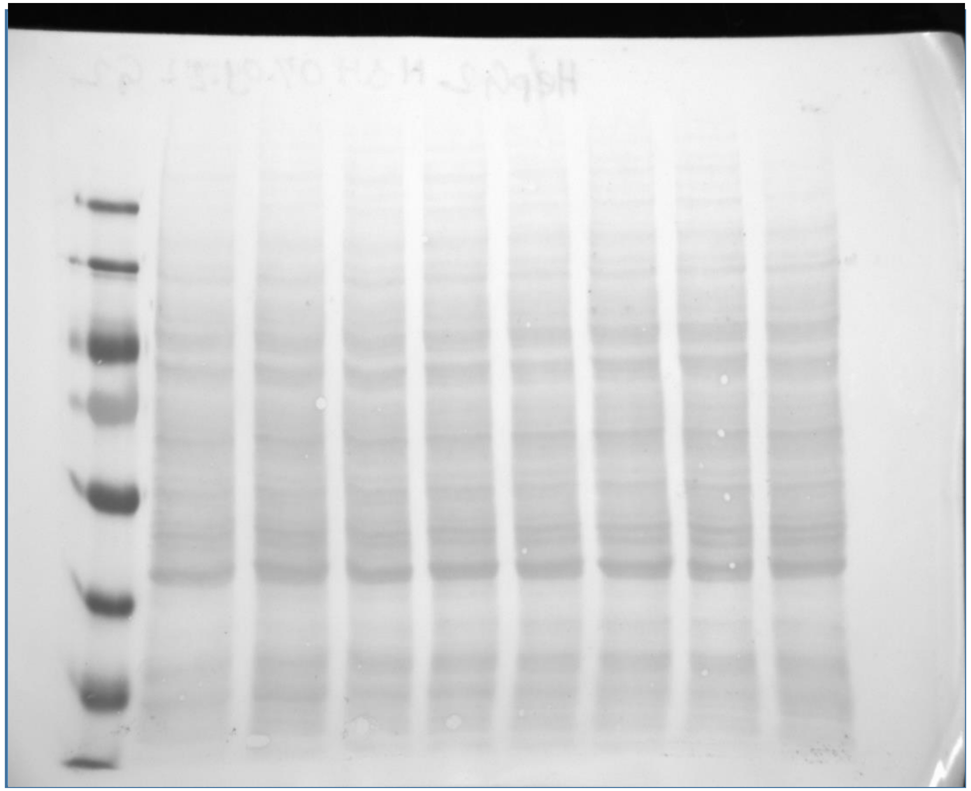

Ponceau

cell type: HepG2, passage x+11
target protein: Lipin1
WB date: 23.09.2022
Incubation: normoxia, normoxia & epl, hypoxia, hypoxia & epl

date of experiment: 07.09.2022
MW: ~107kDa
Marker : PageRuler (Thermo Fisher Scientific)
duration: 24h

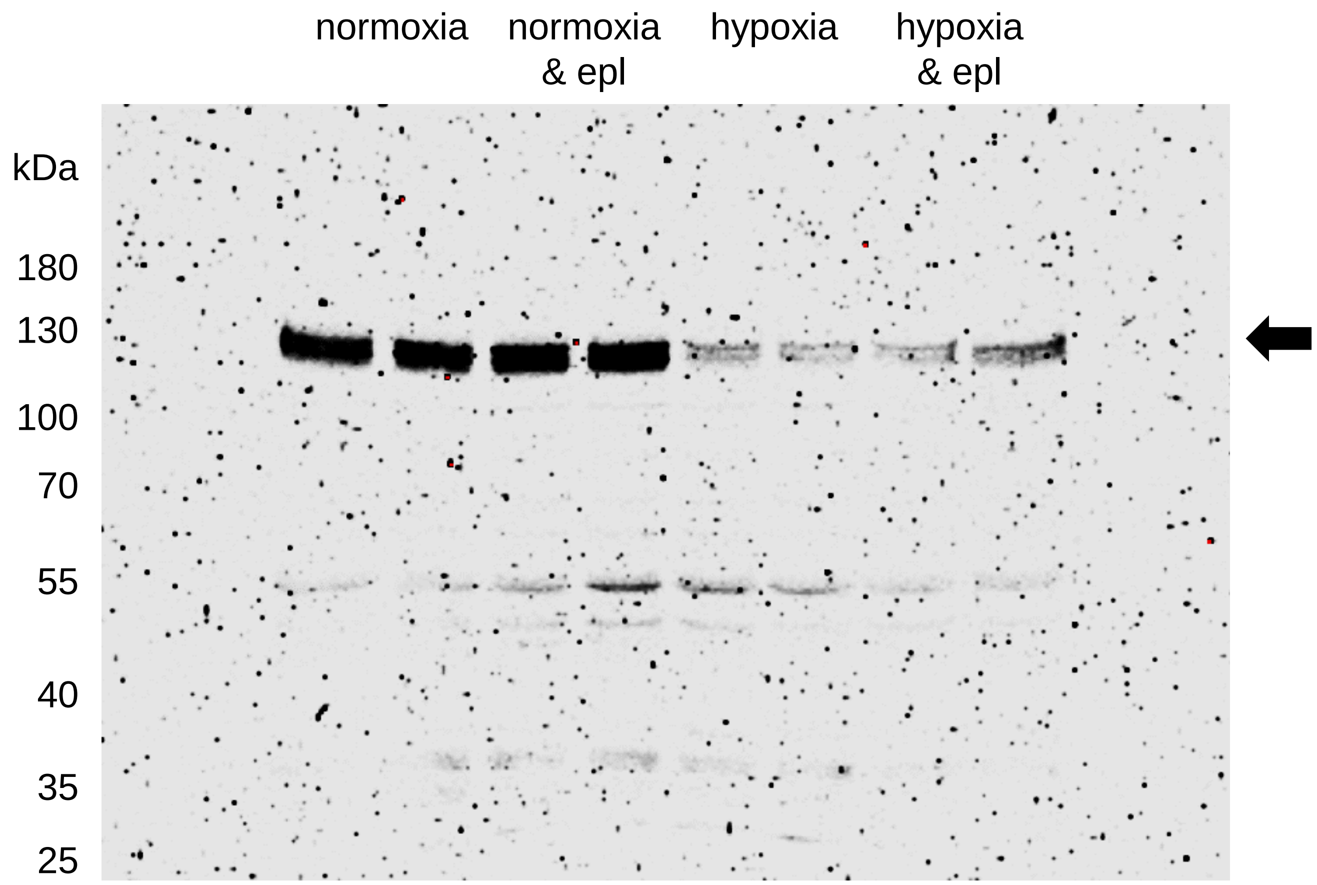

1. AB: Lipin (1:1000) ; #5195; Cell Signaling Technology
2. AB: anti-Rabbit (CellSignaling) 1:2000

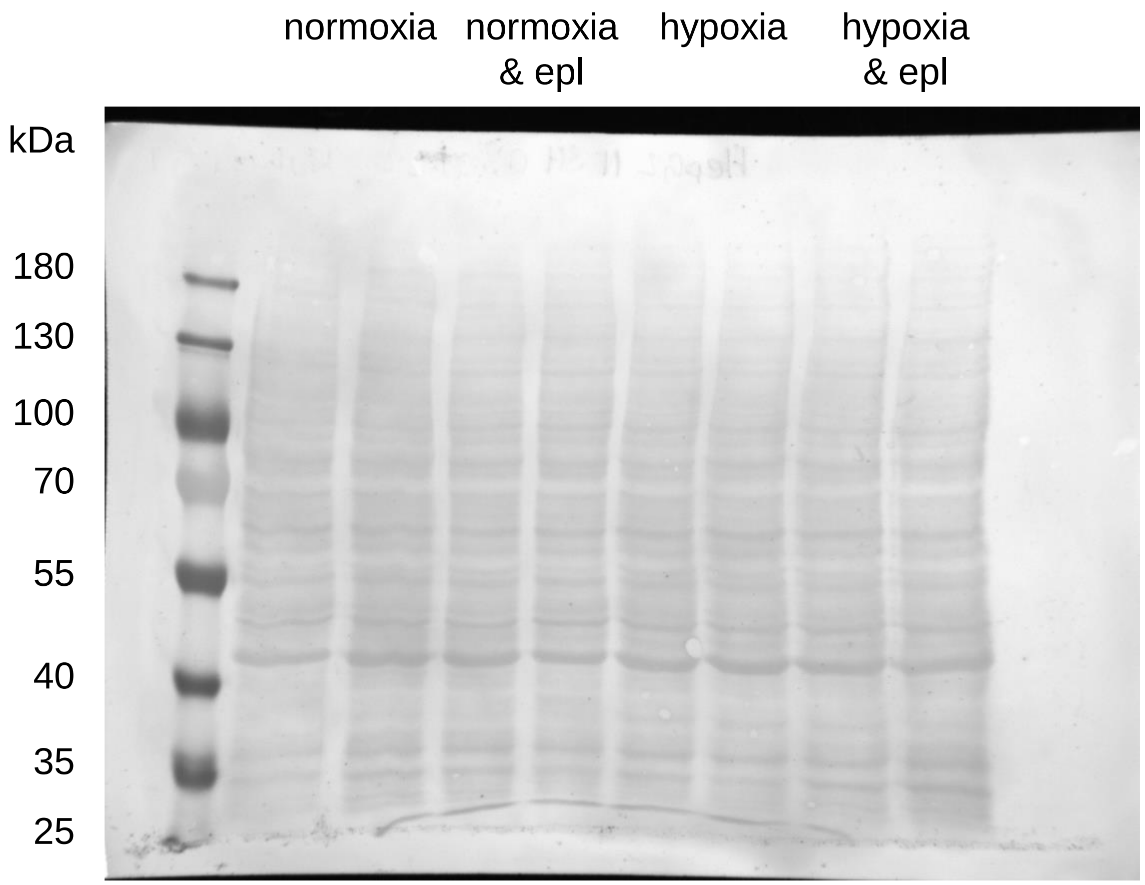

Ponceau

cell type: HepG2, passage x+14

target protein: Lipin1

WB date: 13.12.2022

Incubation: normoxia, normoxia & epl, hypoxia, hypoxia & epl

date of experiment: 14.10.2022

MW: ~130 kDa

Marker : PageRuler (Thermo Fisher Scientific)

duration: 24h

normoxia | normoxia & epl | hypoxia | hypoxia & epl

kDa: 180, 130, 100, 70, 55, 40, 35

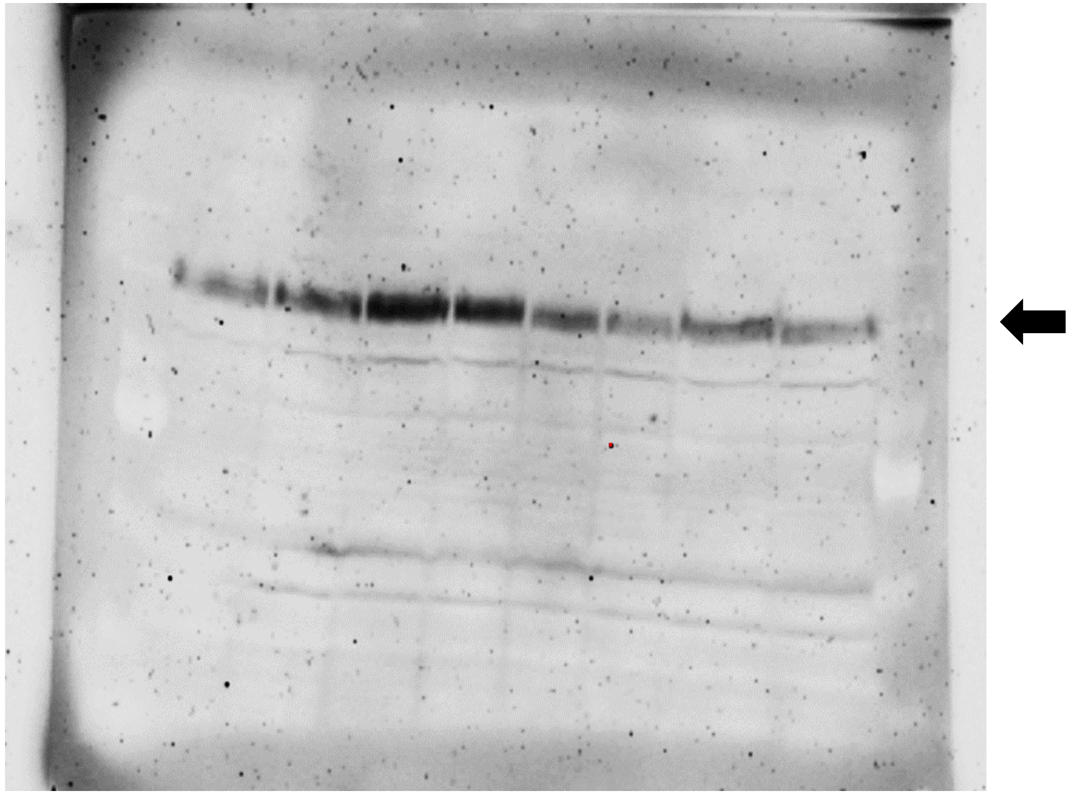

1. AB: Lipin (1:1000) ; #5195; Cell Signaling Technology
2. AB: anti-Rabbit (CellSignaling) 1:2000

normoxia | normoxia & epl | hypoxia | hypoxia & epl

kDa: 180, 130, 100, 70, 55, 40, 35

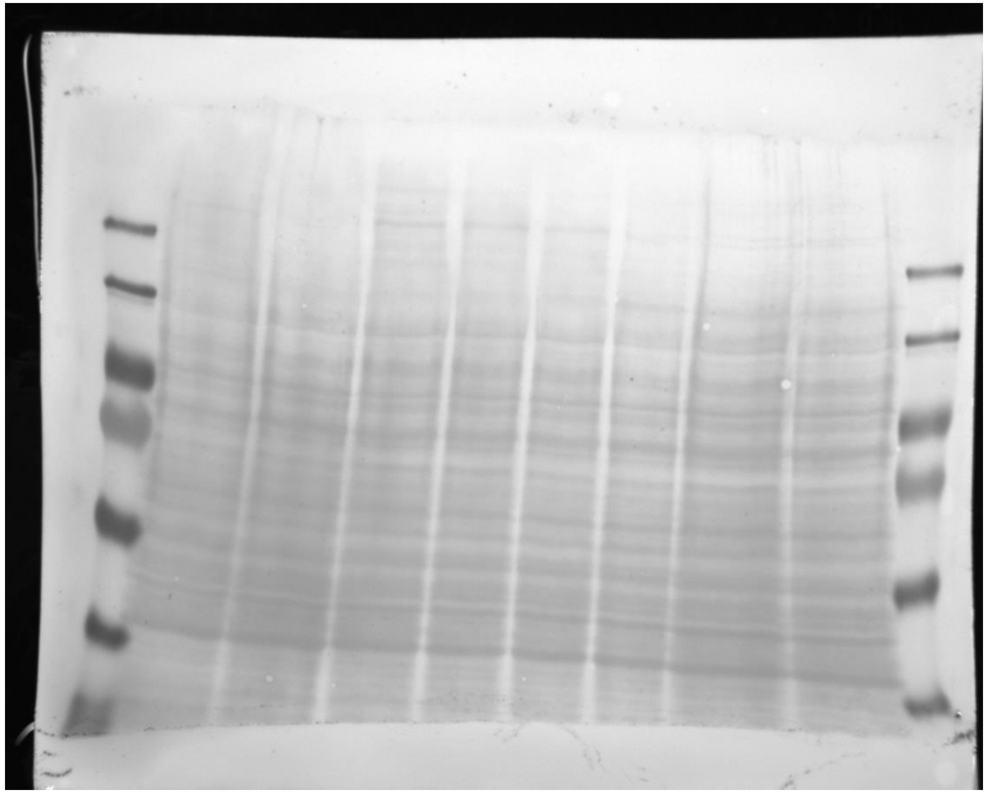

Ponceau
