# Supplementary material for: Eplerenone, a mineralocorticoid receptor inhibitor, reduces cirrhosis associated changes of hepatocyte glucose and lipid metabolism
Source: Cell Commun Signal. 2024 Dec 20;22:614. doi: 10.1186/s12964-024-01991-2 (PMC11660827; doi:10.1186/s12964-024-01991-2)
Supplement: Supplementary file 2 — Supplementary Material 2 [file 12964_2024_1991_MOESM2_ESM.pdf]

SUPPLEMENTARY MATERIAL

EPLERENONE, A MINERALOCORTICOID RECEPTOR INHIBITOR, REDUCES CIRRHOSIS ASSOCIATED CHANGES OF HEPATOCYTE GLUCOSE AND LIPID METABOLISM

Mohammad Mohabbulla Mohib[1], Sindy Rabe[1], Alexander Nolze[1], Michael Rooney[2], Quratul Ain[2], Alexander Zipprich[2], Michael Gekle[1], Barbara Schreier[1]

[1]Julius-Bernstein-Institute of Physiology, Martin Luther University Halle-Wittenberg, Halle, Germany
[2] Department of Internal Medicine IV, Jena University Hospital, Friedrich-Schiller-University Jena, Jena, Germany

Address of correspondence: PD Dr. med. vet. Barbara Schreier, Julius-Bernstein-Institut für Physiologie, Universität Halle-Wittenberg, Magdeburger Strasse 6, 06112 Halle (Saale), Germany, Tel.: ++493455571886; Fax: ++493455574019; E-mail: barbara.schreier@medizin.uni-halle.de

Supplementary figures:

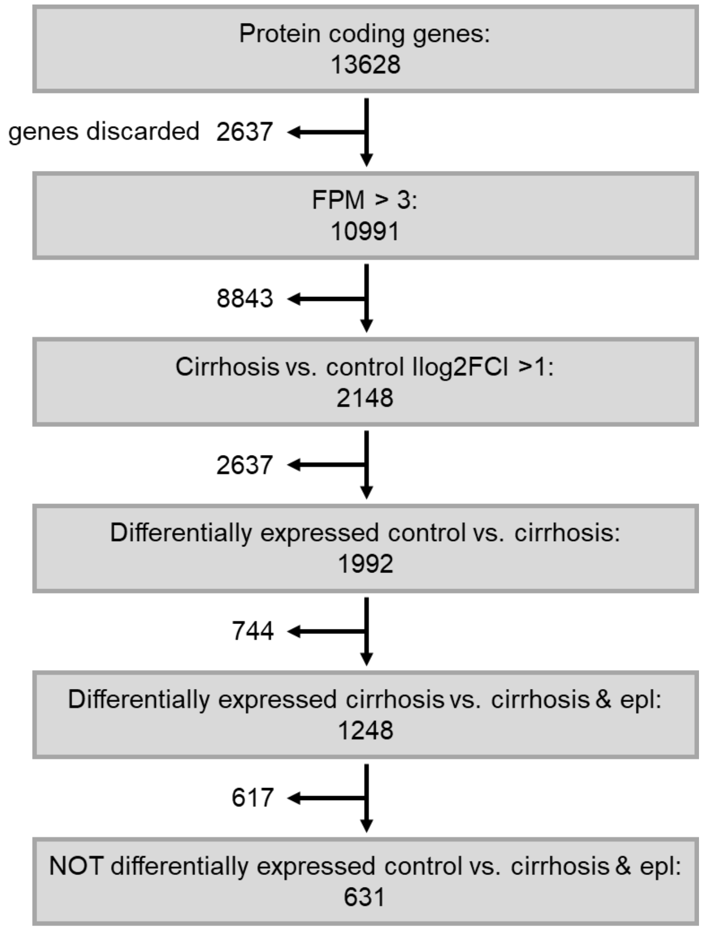

Supplementary figure S1: Flow chart describing the identification of eplerenone-sensitive genes by comparing the confidence intervalls of the different groups.

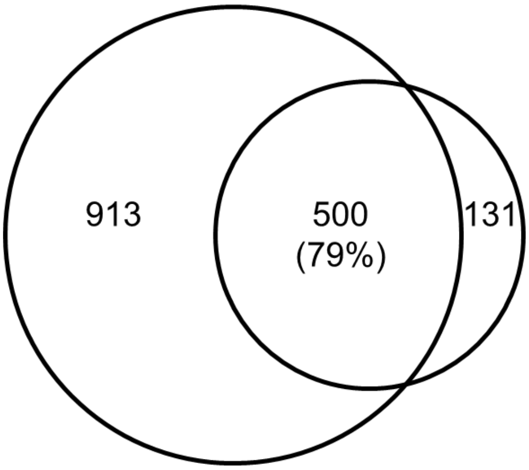

Supplementary figure S2: Venn diagram showing the overlap of the differentially expressed genes identified by either DESeq2 & EdgeR or comparing the confidence intervalls of the three groups.

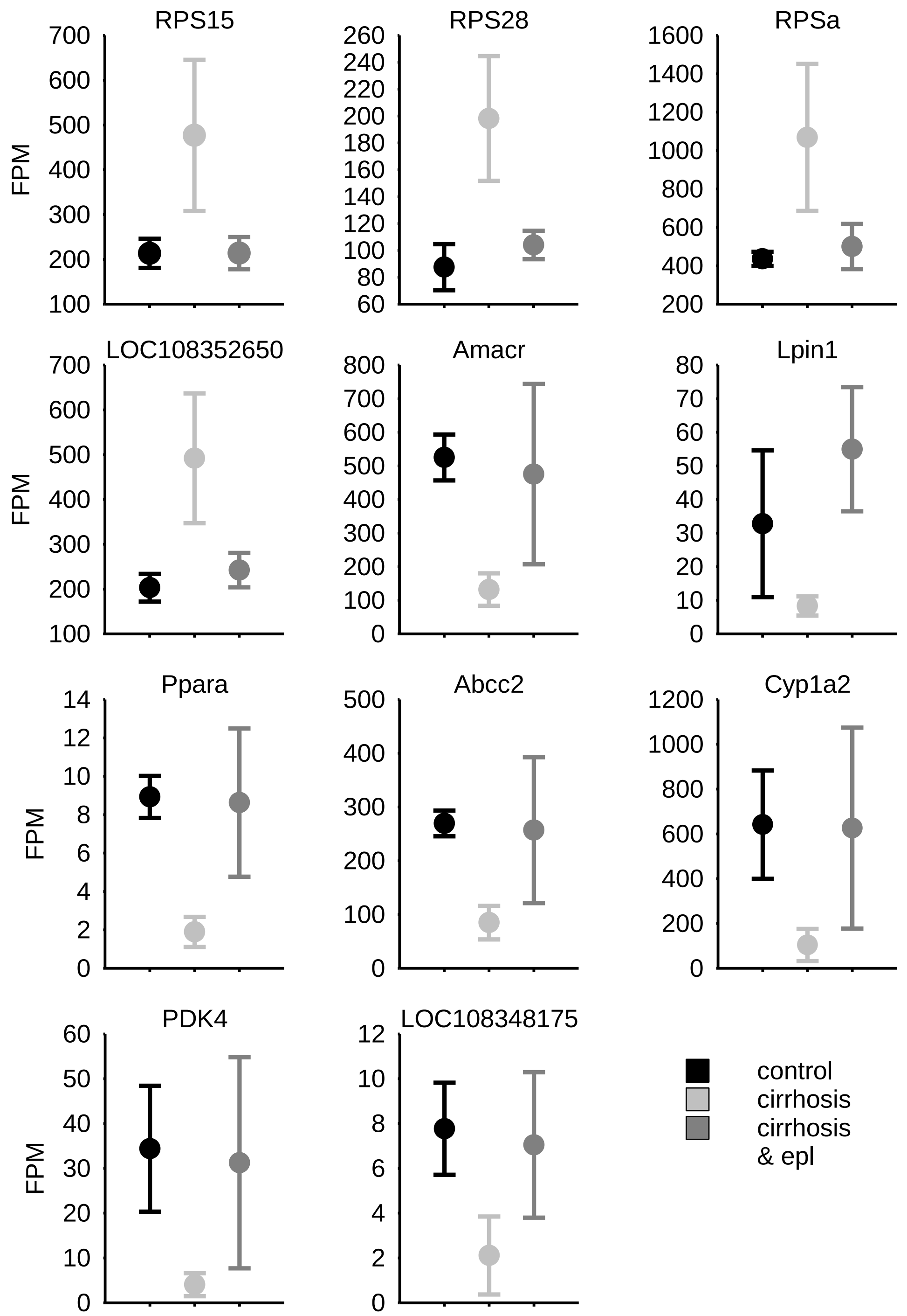

Supplementary figure S3: Mean and confidence intervals for selected genes identified as eplerenone sensitive by RNA sequencing. Black: control group (N =4), light grey: animals treated for 12 weeks with CCl_4 and phenobarbital (cirrhosis, N= 4), dark grey: animals treated for 12 weeks with CCl_4 and phenobarbital and additionally starting after eight weeks with eplerenone (cirrhosis & epl, N = 4).

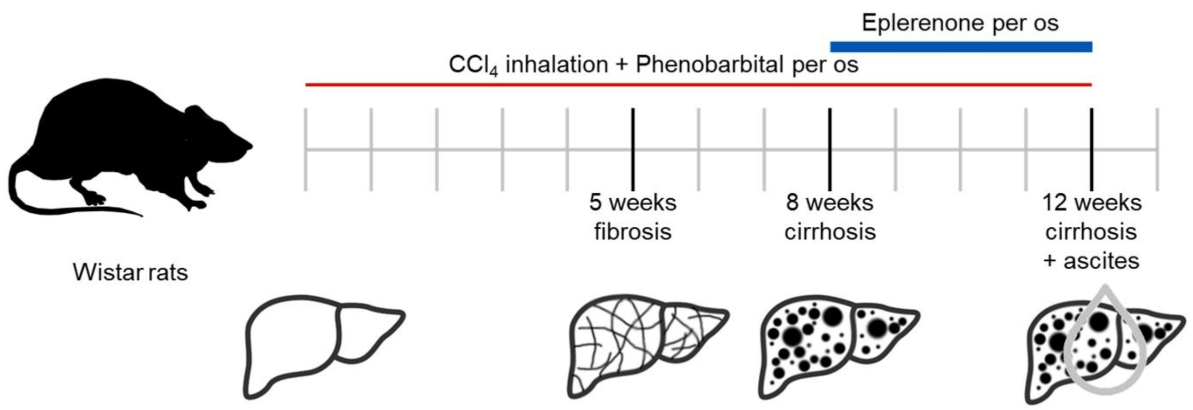

Supplementary Figure S4: Graphical presentation of the treatment protocol for the rats. Rats were treated for 12 weeks with CCl_4 inhalation and phenobarbital per os twice per week. During this time the animals develop decompensated liver cirrhosis. After five weeks usually fibrotic remodeling of the liver can be observed, while after eight weeks cirrhotic nodules without ascites are visible. After eight weeks the animals received a daily dose of eplerenone per os. One week after cessation of treatment the animals were sacrificed and liver tissue was snap frozen for further analysis.

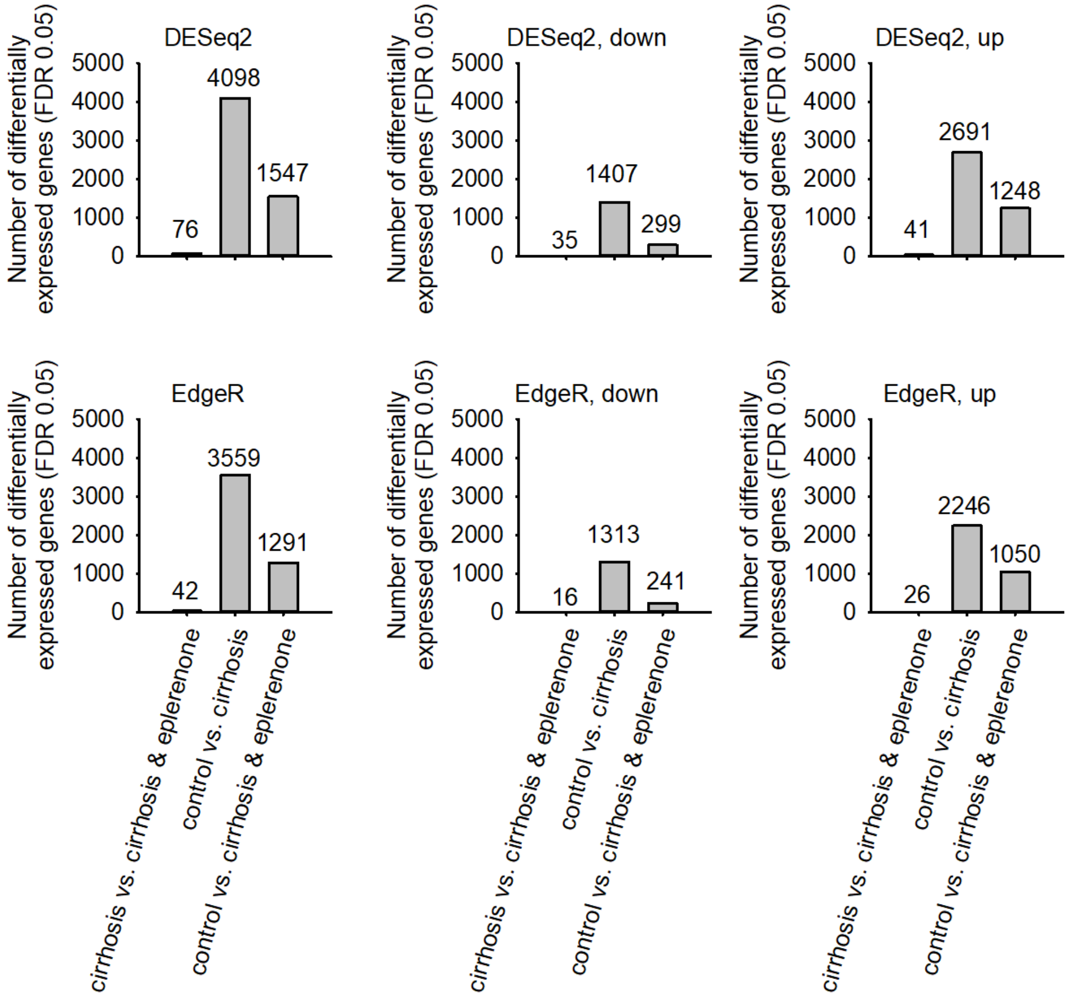

Supplementary Figure S5: Graphical representation of the number of differentially expressed genes obtained by RNA sequencing from rat livers either not treated (control), after 12 weeks of CCl_4 & phenobarbital treatment (cirrhosis) or after 12 weeks of CCl_4 & phenobarbital treatment accompanied by eplerenone treatment for the last four weeks (cirrhosis & eplerenone)

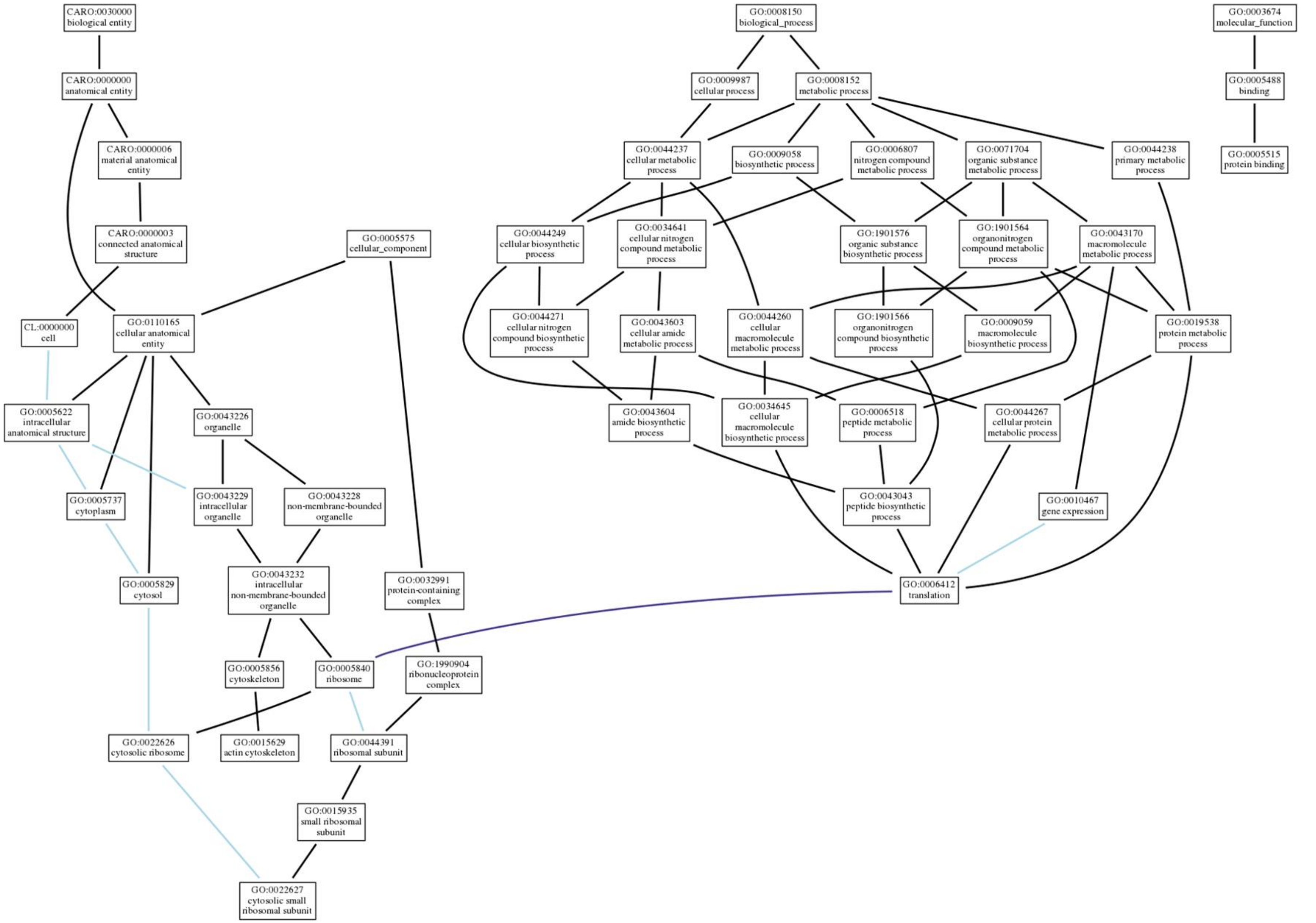

Supplementary figure S6: Ontology tree for GO terms retrieved by GO term enrichment analysis with ep-lerenone sensitive up-regulated genes with an adjusted p-value ≤ 0.05. The ontology tree indicates that the changes observed for the upregulated ep-lerenone sensitive genes cumulate in the GO term “cytosolic small ribosomal subunit”.

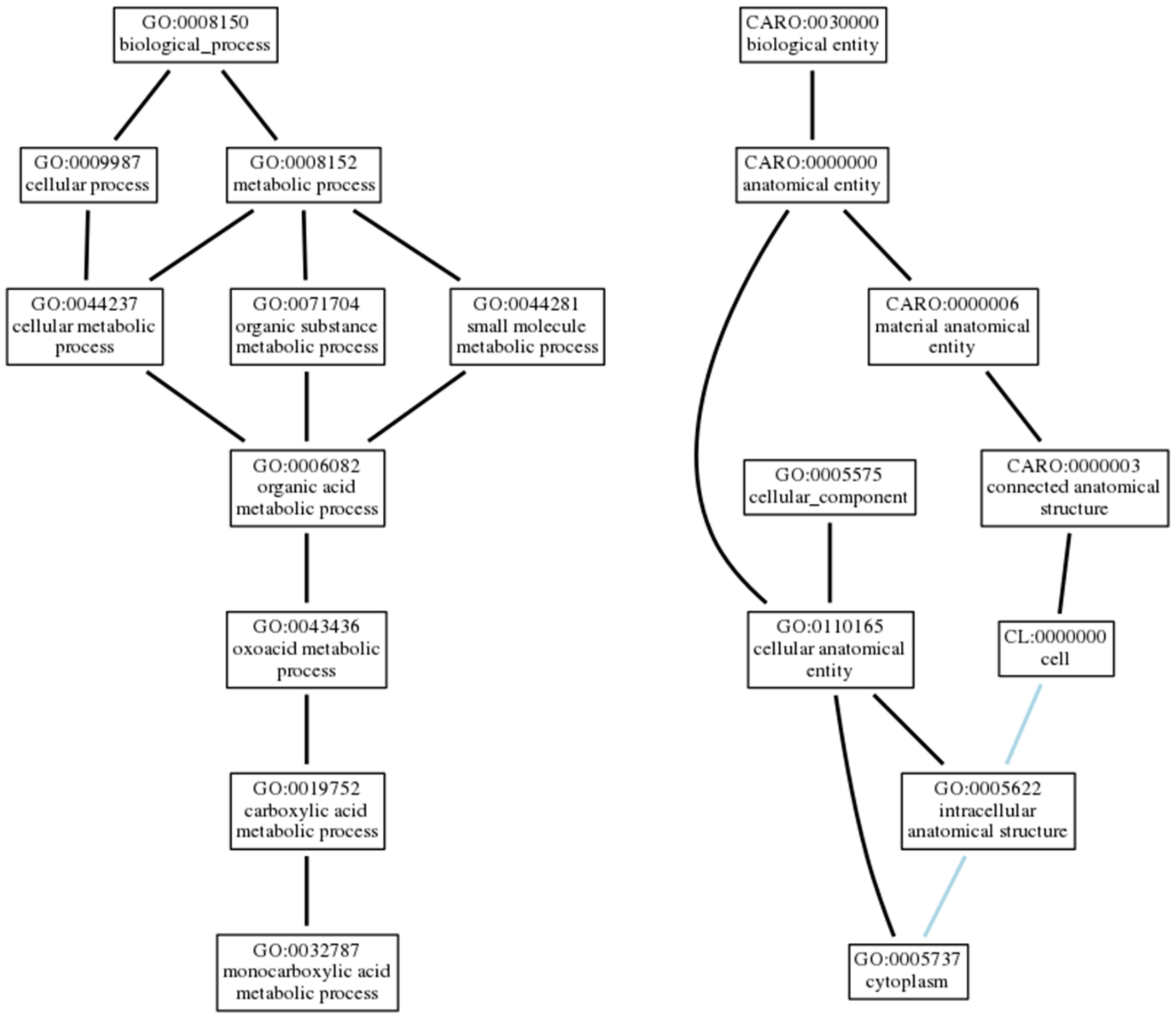

Supplementary figure S7: Ontology tree for GO terms retrieved by GO term enrichment analysis with eplerenone sensitive downregulated genes with an adjusted p-value ≤ 0.05. The ontology tree indicates that the changes observed for the downregulated eplerenone sensitive genes cumulate in either the GO term “monocarboxylic acid metabolic process” or “cytoplasm”. As the enrichment score for the first indicated that a higher portion of the genes annotated to this GO term are altered than for the latter, further analysis was carried out for differentially expressed genes annotated to the GO term “monocarboxylic acid metabolic process”.

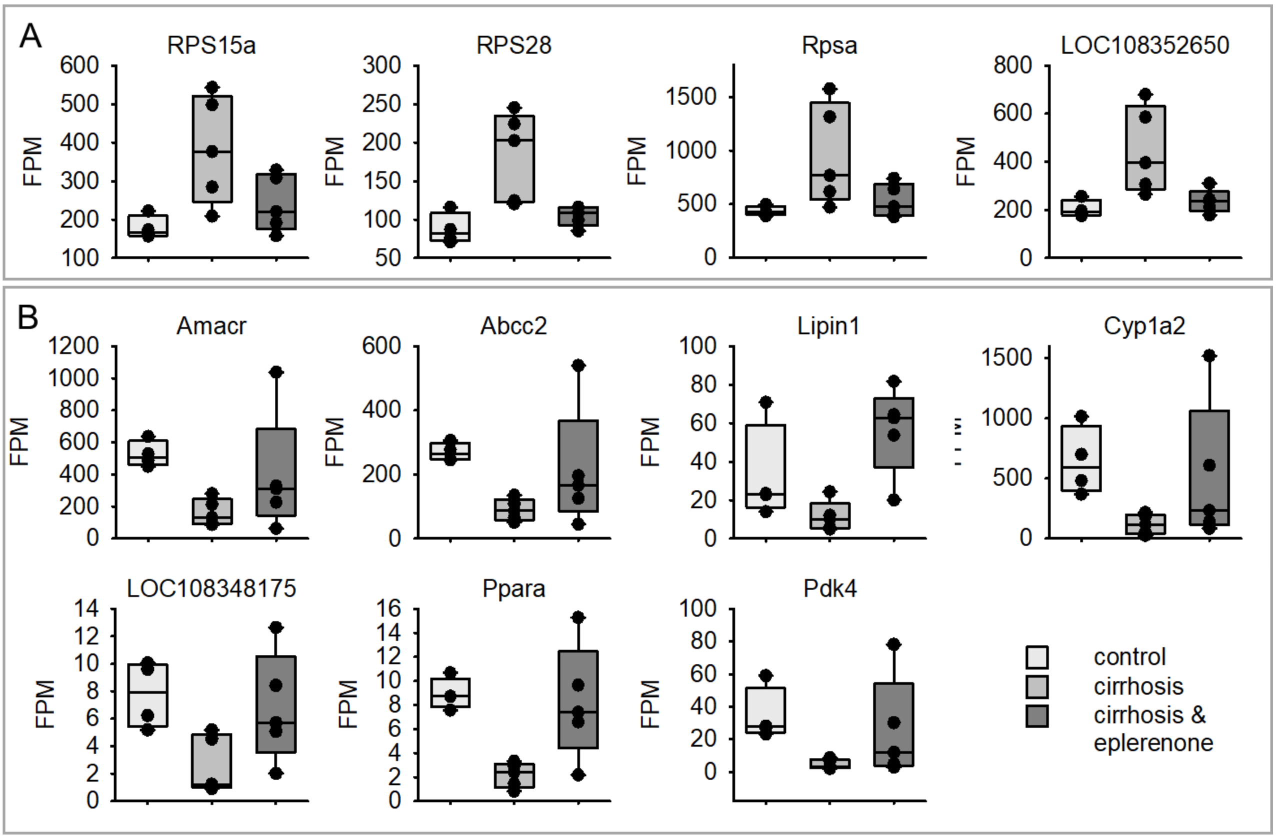

Supplementary figure S8: Representative results from RNA sequencing for differentially expressed eplerenone sensitive genes. A) Upregulated genes annotated to the GO term "cytosolic small ribosomal subunit". B) downregulated genes annotated to the GO term "monocarboxylic acid metabolic process". RNA sequencing was performed on whole liver RNA either from not treated (control) rats or from animals after 12 weeks of CCl_4 & phenobarbital treatment (cirrhosis) or after 12 weeks of CCl_4 & phenobarbital treatment accompanied by eplerenone treatment for the last four weeks (cirrhosis & eplerenone). N = 4-5 animals per group. FPM = fragments per million

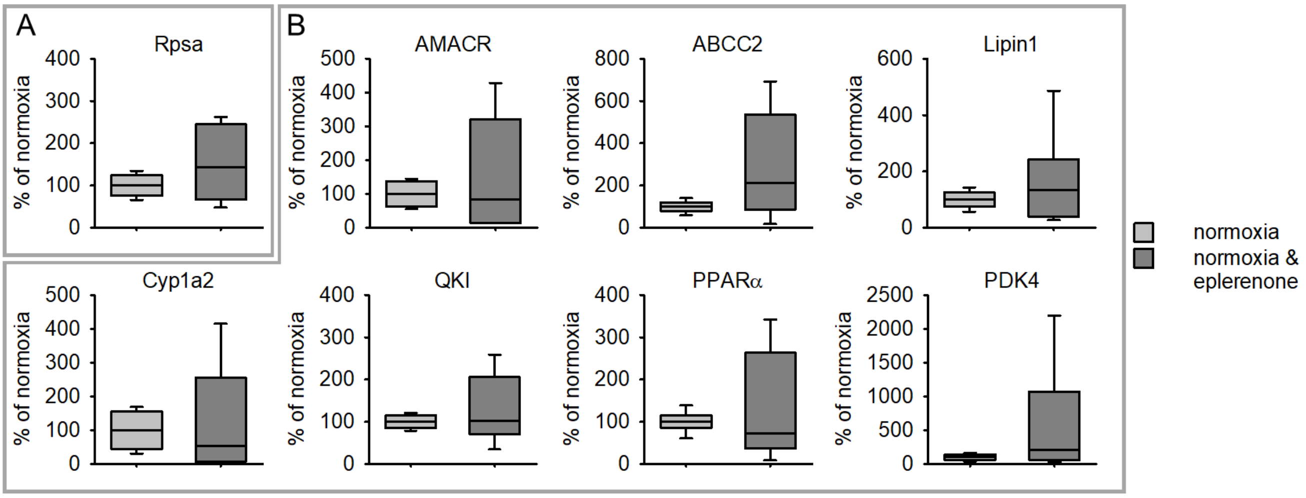

Supplementary figure S9: Change of expression of representative, differentially expressed genes identified by RNA sequencing from cirrhotic rat livers in human HepG2 cell line upon treatment with eplerenone. A) Rpsa belongs to the GO term cytosolic small ribosomal subunit” B) Genes annotated to the GO term “monocarboxylic acid metabolic process”. HepG2 cells were treated for 24h under normoxic (16% oxygen) conditions with or without eplerenone (5µM). N = 5 experiments, n= 9-10 petri dishes/group, p-values are given when below 0.05.

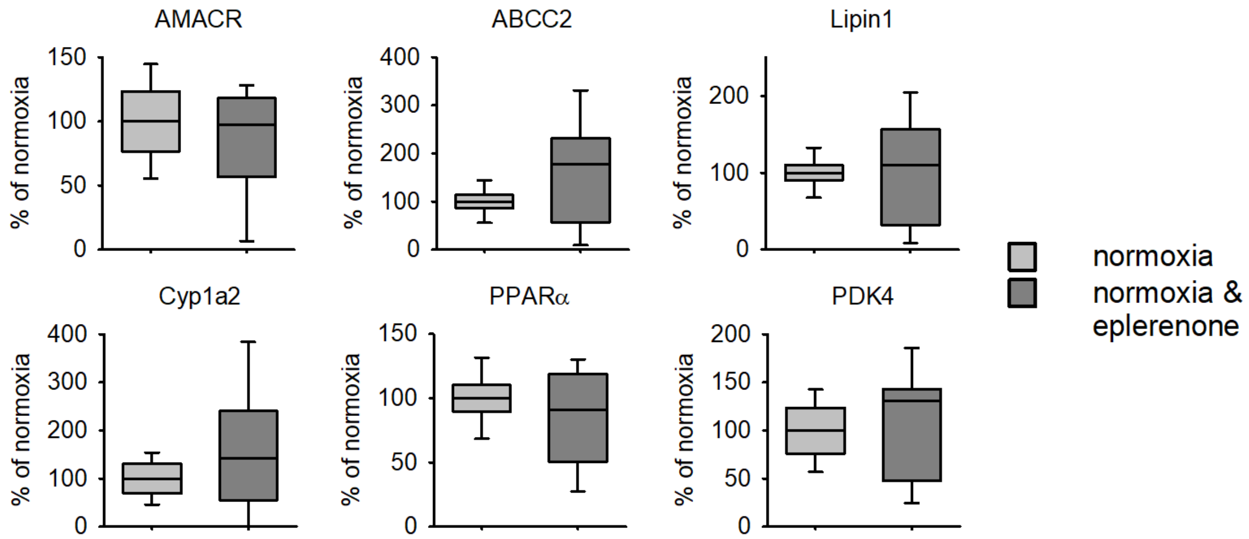

Supplementary figure S10: Change of expression of representative, differentially expressed genes identified by RNA sequencing from cirrhotic rat livers in primary rat hepatocytes upon treatment with eplerenone. pRH were treated for 24h either under normoxic (16% oxygen) conditions with or without eplerenone (5µM). N = 5 experiments, n= 9-10 petri dishes/group, * $p \leq 0.05$ vs. control, p-values are given when below 0.05.

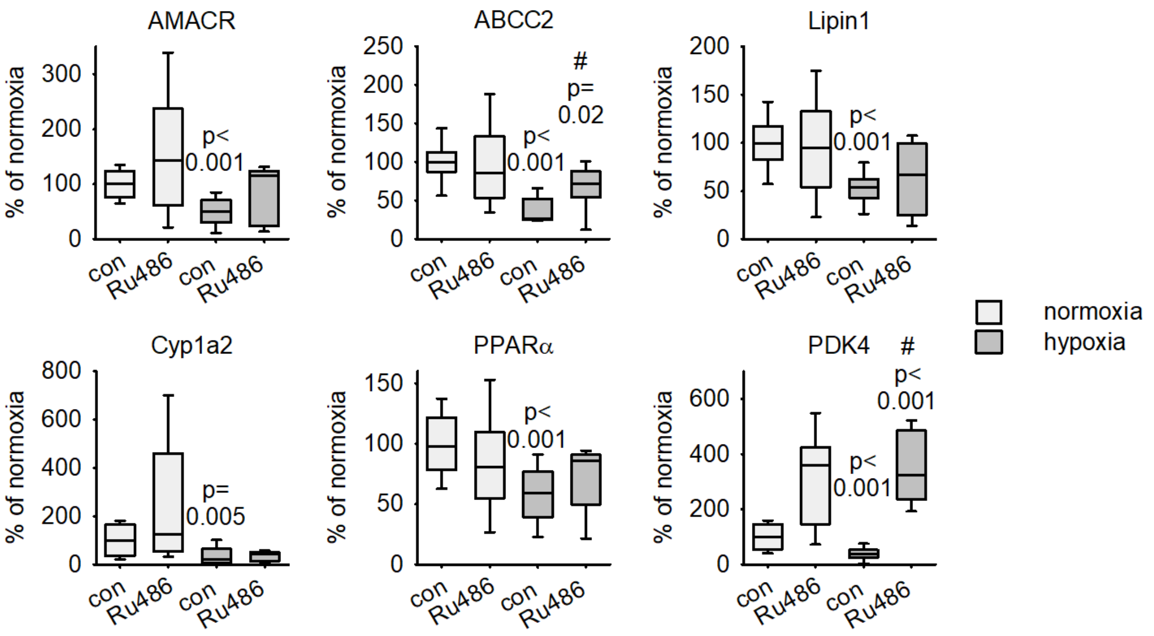

Supplementary figure S11: Impact of RU486 (1µM) and hypoxia (0.2 % O_2) on the expression of representative genes annotated in the GO term "monocarboxylic acid metabolism". N = 3-11 experiments, n= 6-22 petri dishes/group, p-values are given when below 0.05. # indicates the comparison vs. hypoxia, control

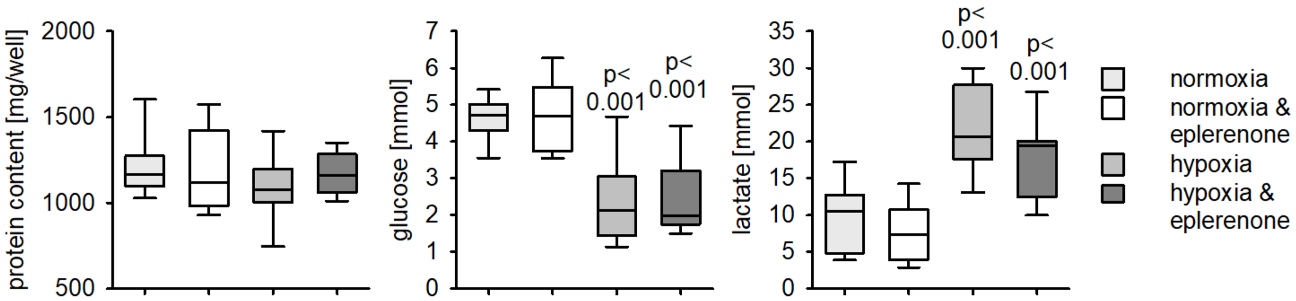

Supplementary figure S12: Protein content, glucose concentration and lactate concentration in cell culture media from HepG2 cells treated for 24h at hypoxic (0.2 % oxygen) or normoxic (16% oxygen) conditions with or without eplerenone (5 µM). N = 7 experiments, n= 13-14 petri dishes/group, p-values are given when below 0.05 for the comparison vs. normoxia, control.

Supplementary tables

Supplementary table S1: Primer for qRT-PCR on cells or tissue from rats

Target	Genebank No.	Sequence sense	Sequence anti	Aneal-ing (°C)	Aneal-ing time (sec)	Frag-ment length	In-tron-span-nimg
SGK1	NM_019232	GGACAACGTCCACCTTCTGT	GGTTTCAGCTGGAGAGGCTT	64	30	199	Yes
PAI-1 (SERPINE1)	NM_012620	GGCCACCAACTTCGGAGTAA	TGTTCCATGACCCCATGAGC	62	30	230	Yes
COL1A1	NM_053304.1	CGGCTCCTGCTCCTCTTAG	GCCATTGTGGCAGATACAGA	58	30	155	Yes
TGFβ	NM_021578	TCCATGACATGAACCGACCC	GAAGTTGGCATGGTAGCCCT	62	30	215	Yes
MCP1	NM_031530.1	CCCAGAAACCAGCCAACT	TGCTGCTGGTGATTCTCTTG	60	20	223	Yes
Fn-1	NM_019143	CACCGAAACCGGGAAGAG	TTGCCTAGGTAGGTCCGTTC	58	30	204	Yes
TNFα	NM_012675.3	CCACCACGCTCTTCTGTCTACTGAACT	CCATTGGCCAGGAGGGCGTT	60	20	226	Yes
Rps15	NM_053982.1	TCATCAGGCCGTGTTCCAAA	CCGTGATGGGAGCAGATTGT	62	30	215	Yes
Rps28	NM_001105730.1	GTGACTCCTCTCCGCTAGAT	TCCTGCCCAGCACTTTAGTTA	60	30	91	Yes
Rps29 (LOC108352650)	NM_012876.1	CGTCTGAAGGCAAGATGGGT	CGTACTGACGGAAGCACTGT	60	30	157	Yes
Rpsa	NM_017138.2	ACTTACACAATGTCCGGAGGC	CCTGAAGGCTGCTTGGATCT	62	30	360	Yes
PPARα	NM_013196.2	CGCTACTTCGAGTCCCCTTG	GTCAGTTCACAGGGAAGGCA	60	30	285	Yes
AMACR	NM_012816.2	AGATGCATGTGTGACCCCAG	TATCCGAGTGCAGCTGATGG	60	30	242	No
ABCC2	NM_012833.2	TGCCCATTATCCGTGCCTTT	GAACAAAGCCCACAACGTCC	60	30	216	Yes
Lipin 1	NM_001012111.1	TAGGAGGTCGGAAGATGGCT	CCATTGTTGGGGACTGGTCA	64	30	90	No
PDK4	NM_053551.2	TCCCGTCTCTACGCCAAGTA	ACTGCCGTAGACCCACTTTG	60	30	280	Yes
Cyp1a2	NM_012541.3	GAGCGAGGAGATGCTCAACC	CTGCCGATCTCTGCCAATCA	59	30	430	Yes
QKI (LOC108348175)	NM_001115021.2	TGCCAAACGGAACTCCTCAC	AGGAGTCAACAGCAGGCACA	64	30	369	No

Suppelementary table S2: Primer for qRT-PCR on HepG2 cells

Target	Genebank No.	Sequence sense	Sequence anti	Aneal-ing (°C)	Anealing time (sec)	Frag-ment length	Intron-span-nimg
SGK1	NM_005627.4	GAGCGGAATGTTCTGTT-GAAGA	CTGGAGAGGCTTGTTCAGAAT	60	30	510	Yes
PAI-1 (SER-PINE1)	NM_000602.3	CAGACCAAGAGCCTCTCC	ATCACTTGGCCCATGAAAAG	56	20	202	Yes
COL1A1	NM_000088.3	TTCTGTACGCAGGTGATTGG	CATGTTCAGCTTTGTGGACC	60	45	129	Yes
TGFβ	NM_000660.6	AAGTTGGCATGGTAGCCCTT	CCCTGGACACCAACTATTGC	60	45	127	Yes
α-SMA	NM_001141945.1	CGAGATCTCACTGACTAC-CTCATGA	AGAGCTACATAACACAG-TTTCTCCTTGA	59	30	116	Yes
CTGF	NM_001901.3	GAAATGCTGCGAGGAGTGG	CGGATGCACTTTTTGCCCTTC	60	30	300	Yes
Rpsa	NM_001304288.2	GGCCAGGTTTGTGGAACAGT	GCGCAGAGGAGAATCTGTGT	62	30	234	Yes
PPARα	NM_005036.6	CATCGGCGAGGATAGTTCTG	GACCACAGGATAAGTCACCG	60	20	139	Yes
AMACR	NM_014324.6	GGCTCTTTTTGACCGCACA	AAATTCACTTGAGCCGTGGG	64	45	680	Yes
ABCC2	NM_000392.5	TGCATCTAGGCAAGGTTAACGA	AGGAGCCATAGGTAGCCCAA	64	30	300	Yes
Lipin 1	NM_001261427.3	GGCTAGGAGTTGGGTGCATT	GCCAAAAACCGTGCACATCT	65	30	225	No
PDK4	NM_002612.4	TCAAGGAGATCTGAATCTC-TACTC	GCTCATCTGATAATGTTTGAAGGC	60	30	136	Yes
Cyp1a2	NM_000761.5	CTTCGGACAGCACTTCCCTG	CTCTAGGCCCCTTCTTGCTG	62	30	278	Yes
QKI	NM_206855.3	AGCTGGCCCTACCATAATGC	TTCGGTCTTCCCTAATGCAAAC	59	30	320	Yes

Supplementary table S3: Effect of aldosterone and eplerenone under normoxic conditions as well as under hypoxic conditions on the expression of mineralocorticoid receptor target genes in primary rat hepatocytes (pRH). pRH were incubated for 24h either with aldosterone (10 nM), eplerenone (5 µM) or both under normoxic or hypoxic (1% oxygen) conditions. N= 3 experiments, n = 4-8 petri dishes/group; p-values vs. respective control are given. a: vs. aldosterone, b: vs. eplerenone, n.d. – not determined, due to the fact that the impact of the different factors was not great enough to exclude the possibility that the difference is just due to random sampling variability.

**% of normoxia control**	**Normoxia**							**Hypoxia**					
**gene of interest**	**control**	**aldosterone**		**eplerenone**		**aldosterone & ep-lerenone**		**control**		**aldosterone**		**eplerenone**	
	**mean ± SD**	**mean ± SD**	**p-value**	**mean ± SD**	**p-value**	**mean ± SD**	**p-value**	**mean ± SD**	**p-value**	**mean ± SD**	**p-value**	**mean ± SD**	**p-value**
SGK-1	100.0 ± 18.1	169.1 ± 57.8	0.06	142.0 ± 77.4	0.36	81.3 ± 56.9	a:0.02 b:0.09	189.5 ± 43.4	0.002	249.2 ± 190.6	n.d.	125.3 ± 85.0	n.d.
PAI-1	100.0 ± 27.5	173.8 ± 56.2	0.003	88.8 ± 11.4	0.63	71.1 ± 24.1	a:<0.001 b:0.44	259.2 ± 143.2	0.151	230.5 ± 59.9	0.9	364.8 ± 361.9	0.62
MCP-1	100.0 ± 5.0	158.5 ± 23.7	0.01	82.9 ± 30.0	0.4	87.8 ± 40.5	a:0.001 b:0.8	85.5 ± 84.3	0.75	58.5 ± 3.8	n.d.	44.9 ± 40.1	n.d.
FN-1	100.0 ± 40.3	306.6 ± 190.2	0.01	148.9 ± 85.4	0.51	127.7 ± 68.6	a:0.02 b:0.76	161.3 ± 45.7	0.04	111.5 ± 56.3	n.d.	148.2 ± 80.2	n.d.
TGF-β	100.0 ± 23.9	99.1 ± 46.4	0.98	161.4 ± 82.2	0.06	72.2 ± 18.0	a:0.35 b:0.01	62.0 ± 15.6	0.01	61.4 ± 18.8	n.d.	45.7 ± 15.7	n.d.
α-SMA	100.0 ± 23.3	237.4 ± 140.8	0.01	160.9 ± 47.0	0.34	106.5 ± 54.9	a:0.01 b:0.3	159.4 ± 31.7	0.01	143.6 ± 43.3	0.6	110.2 ± 26.6	0.03

Supplementary table S4: Effect of aldosterone and eplerenone under normoxic conditions as well as under hypoxic conditions on the expression of mineralocorticoid receptor target genes in a human hepatocyte cell line (HepG2). HepG2 cells were incubated for 24h either with aldosterone (10 nM), eplerenone (5 µM) or both under normoxic or hypoxic (0.2% oxygen) conditions. P-values vs. respective control are given. a: vs. aldosterone, b: vs. eplerenone, n.d. – not determined, due to the fact that the impact of the different factors was not great enough to exclude the possibility that the difference is just due to random sampling variability.

**% of normoxia control**	**Normoxia**							**Hypoxia**					
**Gene of interest**	**control**	**aldosterone**		**eplerenone**		**aldosterone & ep-lerenone**		**control**		**aldosterone**		**eplerenone**	
	**mean ± SD**	**mean ± SD**	**p-value**	**mean ± SD**	**p-value**	**mean ± SD**	**p-value**	**mean ± SD**	**p-value**	**mean ± SD**	**p-value**	**mean ± SD**	**p-value**
**SGK-1**	100 ± 51.3	170.9 ± 100.4	< 0.001	120.0 ± 64.1	0.31	79.1 ± 55.6	a:<0.001 b:0.05	47.6 ± 29.5	0.005	24.8 ± 15.5	0.03	17.3 ± 10.7	0.007
**PAI-1**	100 ± 25.7	133.7 ± 42.2	0.004	93.9 ± 40.7	0.60	98.2 ± 48.7	a:0.002 b:0.71	7376.2 ± 2285.7	< 0.001	5712.8 ± 3032.0	0.13	4744.2 ± 1837.7	0.02
**TGF-β**	100.0 ± 30.4	157.6 ± 91.9	0.001	116.3 ± 56.1.	0.36	89.7 ± 50.1	a:<0.001 b:0.13	376.8 ± 290.6	< 0.001	218.4 ± 91.0	n.d.	188.9 ± 89.0	n.d.

Supplementary table S5: Effect of dexamethasone (100 nM) under normoxic conditions on the expression of eplerenone sensitve genes in a human hepatocyte cell line (HepG2; N= 3-11 experiments, n = 5-24 petri dishes per group)

**% of normoxia control**	**Normoxia**		
**Gene of interest**	**control (mean ± SD)**	**dexamethasone (mean ± SD)**	**p-value**
AMACR	100.0 ± 28.5	167.2 ± 198.4	1.0
ABCC2	100.0 ± 25.5	203.1 ± 178.0	0.33
Lipin1	100.0 ± 25.8	138.2 ± 89.9	0.50
Cyp1a2	100.0 ± 58.1	380.1 ± 439.1	0.14
PPARα	100.0 ± 26.4	110.6 ± 33.6	0.42
PDK4	100.0 ± 45.0	134.1 ± 91.2	0.45

References

1. McGrath JC, Drummond GB, McLachlan EM, et al. Guidelines for reporting experiments involving animals: the ARRIVE guidelines. Br J Pharmacol 2010;160:1573–1576.
2. Schreier B, Wolf A, Hammer S, et al. The selective mineralocorticoid receptor antagonist eplerenone prevents decompensation of the liver in cirrhosis. Br J Pharmacol 2018;175:2956–2967.
3. Zipprich A, Loureiro-Silva MR, Jain D, et al. Nitric oxide and vascular remodeling modulate hepatic arterial vascular resistance in the isolated perfused cirrhotic rat liver. J Hepatol 2008;49:739–745.
4. Friedman SL, Roll FJ. Isolation and culture of hepatic lipocytes, Kupffer cells, and sinusoidal endothelial cells by density gradient centrifugation with Stractan. Anal Biochem 1987;161:207–218.
5. Graupera M, March S, Engel P, et al. Sinusoidal endothelial COX-1-derived prostanoids modulate the hepatic vascular tone of cirrhotic rat livers. Am J Physiol Gastrointest Liver Physiol 2005;288:G763-70.
6. Aurich H, Koenig S, Schneider C, et al. Functional characterization of serum-free cultured rat hepatocytes for downstream transplantation applications. Cell Transplant 2005;14:497–506.
7. Dubourg V, Schreier B, Schwerdt G, et al. The Functional Interaction of EGFR with AT1R or TP in Primary Vascular Smooth Muscle Cells Triggers a Synergistic Regulation of Gene Expression. Cells 2022;11.
8. Kim D, Paggi JM, Park C, et al. Graph-based genome alignment and genotyping with HISAT2 and HISAT-genotype. Nat Biotechnol 2019;37:907–915.
9. Liao Y, Smyth GK, Shi W. featureCounts: an efficient general purpose program for assigning sequence reads to genomic features. Bioinformatics 2014;30:923–930.
10. Durinck S, Spellman PT, Birney E, et al. Mapping identifiers for the integration of genomic datasets with the R/Bioconductor package biomaRt. Nat Protoc 2009;4:1184–1191.
11. Robinson MD, McCarthy DJ, Smyth GK. edgeR: a Bioconductor package for differential expression analysis of digital gene expression data. Bioinformatics 2010;26:139–140.
12. Love MI, Huber W, Anders S. Moderated estimation of fold change and dispersion for RNA-seq data with DESeq2. Genome Biol 2014;15:550.

13. Reimand J, Arak T, Adler P, et al. g:Profiler-a web server for functional interpretation of gene lists (2016 update). Nucleic Acids Res 2016;44:W83-9.

14. Carbon S, Ireland A, Mungall CJ, et al. AmiGO: online access to ontology and annotation data. Bioinformatics 2009;25:288–289.

15. Gomez-Sanchez CE, Warden M, Gomez-Sanchez MT, et al. Diverse immunostaining patterns of Mineralocorticoid Receptor monoclonal antibodies. Steroids 2011;76:1541–1545.

16. Schwerdt G, Kopf M, Gekle M. The nephrotoxin ochratoxin a impairs resilience of energy homeostasis of human proximal tubule cells. Mycotoxin Research 2023.

17. Kraus NA, Ehebauer F, Zapp B, et al. Quantitative assessment of adipocyte differentiation in cell culture. Adipocyte 2016;5:351–358.

18. Sadick JS, Boutin ME, Hoffman-Kim D, et al. Protein characterization of intracellular target-sorted, formalin-fixed cell subpopulations. Sci Rep 2016;6:33999.

19. Addis MF, Tanca A, Pagnozzi D, et al. Generation of high-quality protein extracts from formalin-fixed, paraffin-embedded tissues. Proteomics 2009;9:3815–3823.

20. Ikeda K, Monden T, Kanoh T, et al. Extraction and analysis of diagnostically useful proteins from formalin-fixed, paraffin-embedded tissue sections. J Histochem Cytochem 1998;46:397–403.

21. Jiang X, Jiang X, Feng S, et al. Development of efficient protein extraction methods for shotgun proteome analysis of formalin-fixed tissues. J Proteome Res 2007;6:1038–1047.
